# Eculizumab withdrawal and monitoring in atypical haemolytic uraemic syndrome (SETS aHUS): a multicentre, open label, prospective, single arm trial

**DOI:** 10.1016/j.lanepe.2025.101392

**Published:** 2025-08-07

**Authors:** Andrew Bryant, Jan Lecouturier, Giovany Orozco-Leal, Victoria Brocklebank, Sonya Carnell, Thomas Chadwick, Sarah Dunn, Sally Johnson, David Kavanagh, Ciara Kennedy, Michael Malina, Emma Montgomery, Colin Muirhead, Yemi Oluboyede, Luke Vale, Chris Weetman, Edwin Wong, Neil S. Sheerin, SETS aHUS Consortium, Coralie Bingham, Paramit Chowdhury, Tina Dutt, Eric Finlay, Daniel Gale, Amrit Kaur, Michael Kelly, Stephen Marks, Mordi Muorah, Ravindra Rajakariar, Nicholas Torpey, Mark Uniake, David Walbaum, Pallavi Yadav

**Affiliations:** a Population and Health Sciences Institute, Newcastle University, Newcastle upon Tyne, UK; b Newcastle Clinical Trials Unit, Newcastle University, Newcastle upon Tyne, UK; c Department of Health Services Research and Policy, London School of Hygiene and Tropical Medicine, UK; d National Renal Complement Therapeutic Centre, Newcastle Upon Tyne Hospitals NHS Foundation Trust, Newcastle upon Tyne, UK; e Newcastle University Translational and Clinical Research Institute, Newcastle University, Newcastle upon Tyne, UK

**Keywords:** Atypical haemolytic uraemic syndrome, Eculizumab, Treatment withdrawal, Complement, Relapse prediction

## Abstract

**Background:**

Atypical Haemolytic Uraemic Syndrome is a rare disease, associated with high morbidity and mortality. Eculizumab, a monoclonal complement inhibitor, is an effective treatment but the optimal way to use this high-cost medication has not been determined. The SETS aHUS trial aimed to establish the safety of eculizumab withdrawal and the effectiveness of a monitoring protocol to detect disease relapse and reintroduction of treatment if relapse occurs.

**Methods:**

The SETS aHUS multicentre, open label, prospective, single-arm trial enrolled participants from 15 UK hospitals. Patients over two years of age with aHUS who were receiving eculizumab therapy for at least six months were eligible to withdraw from treatment, replacing it with monitoring to assess disease activity and treatment re-introduction if relapse occurred. The primary outcome measure was harm to a participant as a consequence of eculizumab withdrawal. Participants met a primary outcome if there was a permanent reduction in estimated glomerular filtration rate, requirement for kidney replacement therapy or significant extra-renal manifestation of disease. The Bayes factor single arm binary model was used to monitor and analyse the trial data, applying pre-trial stopping rules. The trial is registered with the European Union Drug Regulating Authority (EudraCT 2017-003916-37) and is closed for recruitment.

**Findings:**

One of 28 participants (3.6%) who withdrew from treatment met a primary outcome. Four of the 28 participants (14.3%) relapsed. Only participants with an identified cause of complement dysregulation relapsed. It was possible, by monitoring and rapid participant access, to reintroduce eculizumab treatment. Based on the pre-trial analysis plan, withdrawal from treatment may represent no greater risk to patients.

**Interpretation:**

In this single arm study, for patients fulfilling trial entry criteria, which excluded some high-risk patients, withdrawal of eculizumab treatment with monitoring of disease activity was not associated with an increased risk of harm compared to continuation of eculizumab.

**Funding:**

10.13039/501100000664National Institute for Health and Care Research Health Technology Assessment programme.

**Research in context**

**Evidence before this study**

Eculizumab was approved for the treatment of atypical haemolytic uraemic syndrome (aHUS) based on the results of single arm studies with a follow up period of 26 weeks. The regulatory approval for eculizumab was for indefinite treatment unless there was a medical indication to stop, reflecting the high morbidity and mortality previously associated with aHUS. Subsequent publications reported that patients maintained on eculizumab treatment remained free of disease. The National Institute of Clinical and Health Care Excellence approved eculizumab for aHUS provided there was a research programme with robust methods to evaluate when stopping treatment or dose adjustment might occur. This was in response to both the high cost of eculizumab (estimated to be £340,000 for the first year of treatment for an adult) and the lack of evidence that long-term treatment was required. A Pubmed search prior to the start of trial (2018) using the terms “eculizumab” and “atypical haemolytic syndrome” identified case series and case reports of eculizumab withdrawal. No prospective trial evaluating eculizumab withdrawal was identified.

**Added value of this study**

STES aHUS is the first trial to apply statistical methods to evaluate whether withdrawal of eculizumab treatment in patients with aHUS and replacement with a monitoring protocol is feasible. The primary outcome was harm to patients with continuous monitoring during the trial for aHUS-related adverse events. This trial demonstrated that in eligible patients eculizumab treatment can be replaced with a schedule of monitoring for disease activity and provides evidence for alternative treatment strategy for aHUS. Atypical HUS is a rare disease with an incidence of 0.4/million/year which makes randomised trials very difficult and no randomised trial has been yet been published in this disease. Although not providing the same level of confidence as a two-arm trial, the application of Bayesian statistical techniques in this single-arm trial allows evaluation of an intervention in rare disease when historical data on outcomes is available.

**Implications of all the available evidence**

The results of the SETS aHUS trial, along with other published observational studies, support the use of eculizumab for a time-limited period to treat aHUS during periods of disease activity in the majority of patients. Treatment can then be replaced by monitoring of disease activity, reducing the risk and burden of treatment on patients and cost to health care providers. Combining data from all studies allows a more accurate prediction of the risk of disease relapse, improves informed decision making, permits stratification of monitoring intensity and is likely to lead to more widespread implementation of eculizumab treatment for a limited period to treat active disease.

## Introduction

Atypical haemolytic uraemic syndrome (aHUS) is an extremely rare, severe disease with an incidence of 0.41/per million/year in the UK,^1^ and a prevalence of approximately 4.9 people per million population in Europe.^2^ Atypical HUS causes a thrombotic microangiopathy (TMA) which is clinically characterised by thrombocytopaenia, microangiopathic haemolytic anaemia and Acute Kidney Injury (AKI), although other organ involvement can occur. The most common cause of aHUS is uncontrolled complement activation, which damages the endothelium of the microvasculature leading to thrombus formation.^3^^,^^4^ This excessive complement activation can be caused at a genetic level by loss of function mutations in key complement regulatory proteins or gain of function mutations in complement activating proteins. Autoantibodies that interfere with the function of Factor H can also cause aHUS.^5^ Mutations or autoantibodies affecting complement are identified in approximately 60% of aHUS cases.^3^^,^^6^ Complement inhibition with an anti-C5 monoclonal antibody is a very effective treatment for aHUS and its introduction has significantly improved patient outcomes.^1^^,^^7^ However, the optimal duration of treatment is unknown and there is increasing evidence that indefinite treatment, as initially proposed, is not required.

Eculizumab is a humanised monoclonal antibody that inhibits the function of C5, a key protein involved in complement activation. A pivotal clinical trial published in 2013 reported that eculizumab treatment of patients with aHUS was associated with significant time-dependent improvement in renal function over a 26-week follow up period.^7^ This benefit was confirmed in further clinical trials in both children and adults,^8^^,^^9^ and persisted during a two year extension study.^10^ In the UK, eculizumab treatment in aHUS was subject to an evaluation by the National Institutes for Health and Care Excellence (NICE).^11^ In this evaluation, eculizumab was approved for use in aHUS conditional on the implementation of a “research programme with robust methods to evaluate when stopping treatment or dose adjustment might occur”.^11^

In aHUS, lifelong treatment with eculizumab was recommended due to the high risk of relapse based on historical observational data.^6^ As an intrinsic part of the innate immune system, the complement pathway is essential for the prevention of *Neisseria meningitidis* infection and eculizumab treatment is associated with a 500–1000 fold increased risk of infection despite vaccination and prophylactic antibiotics.^1^^,^^12^^,^^13^ Consequently, along with the burden of treatment on patients and the financial impact on health-care providers, many physicians and healthcare systems are questioning the recommendation of indefinite treatment.^14^, ^15^, ^16^

The primary aim of the SETS aHUS trial was to establish an evidence base for an alternative management strategy for patients with aHUS that includes the use of eculizumab to establish remission, withdrawal of treatment with monitoring of disease activity and the reintroduction of eculizumab in those patients who relapse. Uniquely this trial applies Bayesian statistical methodology to test this alternative treatment strategy in contrast to the existing observational cohort reports.

## Methods

### Trial design and participants

This is a single arm, open label trial of eculizumab withdrawal in patients with aHUS, which was conducted in 15 UK National Health Service Hospitals. The trial was conducted in accordance with the Declaration of Helsinki and a favourable ethical opinion and approval was obtained from the North East - Tyne & Wear South Research Ethics Committee in April 2018 (18/NE/0078). Consent was obtained from participants or from the parents/legal guardian on behalf of participants under the age of 16 years. Trial oversight was provided by independent trial steering and data monitoring committees (Appendix p33). As described in the published protocol the trial included both qualitative and health economic components that are reported elsewhere.^17^

**Fig. 1:**
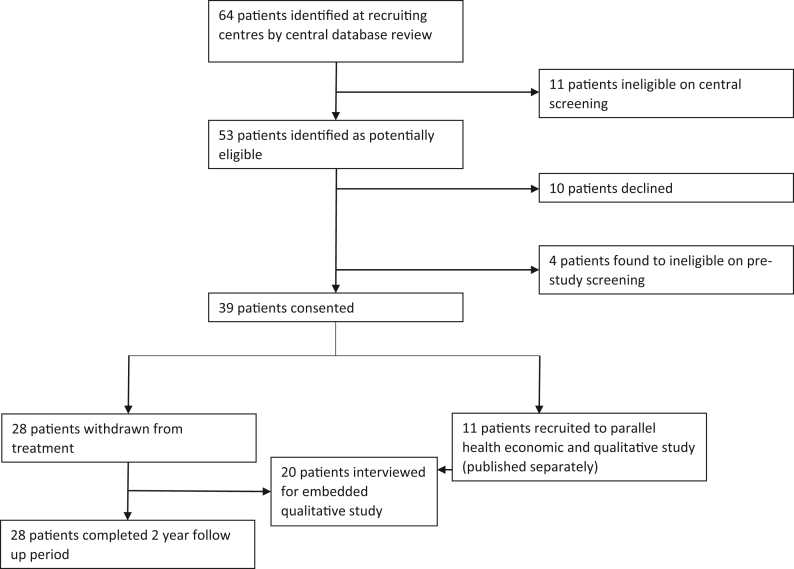
**Trial profile.** Participants were consented to withdraw from eculizumab treatment or to enter a parallel health economic and qualitative study (reported elsewhere) and remained on eculizumab treatment.

Eligible patients were identified from the database held by the NHS England commissioned national aHUS centre in Newcastle upon Tyne.^18^ Patients aged over two years who had been on eculizumab for at least six months were eligible. Disease had to be in remission with no evidence of ongoing microangiopathic haemolytic anaemia (MAHA), defined by a Lactate Dehydrogenase (LDH) <×2 upper limit of activity, and a normal platelet count at screening. Participants had normal renal function or chronic kidney disease (CKD) stages 1–3 (estimated Glomerular Filtration Rate (eGFR) >30 ml/min/1.73 m^2^) which had been stable for six months prior to recruitment. Patients were excluded if they had poorly controlled blood pressure (systolic blood pressure >160 mmHg), if pregnant or planning pregnancy, or had severe disease manifestations at presentation which in the opinion of the investigators made the risk of withdrawal unacceptable. The presence of haematuria (3+) at screening did not allow self-monitoring and therefore, if present, patients were excluded from the trial. In addition, kidney transplant recipients were excluded if they had lost a previous transplant to recurrent aHUS or had a pathogenic variant in *C3*, *CFH* or *CFB* (Fig. 1). A full list of inclusion and exclusion criteria is in the Appendix (p5–7).

### Procedures

After consent, participants received their last dose of eculizumab on day −14 with day 0 being the day that the participants would usually receive their next dose of eculizumab (Appendix p5–32). Meningococcal prophylactic antibiotics were continued for a further two weeks after day 0. At day 0 and at pre-defined time points (Appendix p7–10) bloods samples were taken to assess renal function (serum creatinine and eGFR) and haematological parameters of TMA activity (haemoglobin, platelet count, blood film, LDH, and haptoglobin). Renal function and TMA activity was also assessed at unscheduled visits if the participant developed symptoms that could suggest disease activity. Estimated GFR was calculated using the CKD-EPI without correction for ethnicity and modified Schwartz equations for adults and children respectively.^19^^,^^20^ Measurement of urinary protein to creatinine ratio and urinalysis for the level of haematuria was performed.

Participants, or their parent/legal guardian, were trained to perform home urinalysis (Siemens Healthcare Diagnostics Ltd). Urinalysis, for the presence of haematuria or haemoglobinuria as an indicator of intravascular haemolysis and disease activity, was performed daily by the participant or parent/legal guardian for the first month and then three times per week for the remainder of the trial period. The results were recorded in a participant diary, which was reviewed at each visit. Participants or their parent/legal guardian were asked to report any significant change in urinalysis from the screening result, not related to menstruation (increase of ≤2+ on one test or ≤1+ on two consecutive tests). If this occurred, participants attended for blood tests to assess renal function and TMA activity.

Relapse diagnosis was based on one of the following criteria: 1. Haematological relapse defined as thrombocytopaenia (platelet count of <150 × 10^9^/l or a fall in platelet count by >50% from screening) or MAHA (increase in LDH by >50% above screening or haemoglobin < lower limit of normal for age and gender), 2. Renal involvement defined as Acute Kidney Injury Network stage 1 AKI (confirmed on repeat testing after 6 h) that is not explained by another pathology, 3. The presence of histological features of an active TMA on tissue biopsy.

When a relapse was diagnosed, participants restarted eculizumab treatment with the aim that this should occur within 24 h of relapse confirmation provided there was no evidence of a severe, active infection that would be a contraindication to treatment. Eculizumab was restarted at the recommended dose of 900 mg weekly for the first four weeks then 1200 mg on week five, then every two weeks thereafter (or age adjusted dose and regime in children). Renal function and TMA activity was monitored as recommended by attending clinician until haematological remission was achieved. Participants who relapsed and required re-introduction of eculizumab treatment remained on treatment and under follow up for the full two years of the trial. Home urinalysis was not required after re-introduction of eculizumab.

### Outcomes

The primary outcome was harm to the participant due to relapse of aHUS following withdrawal of eculizumab (Appendix p3–4). This was defined as; irreversible (>3 months) reduction of greater than 20% in eGFR not attributable to another cause, an episode of AKI attributed to a TMA that requires renal replacement therapy, or a non-renal manifestation of a TMA that requires hospitalisation, causes irreversible organ damage or death. Whether a participant met the primary outcome was adjudicated by the independent data monitoring committee.

Secondary outcomes included assessment of the effectiveness of the monitoring protocol to detect disease relapse following withdrawal of eculizumab determined by the proportion of participants who relapse and restart eculizumab without reaching a primary outcome and the time from the first clinical feature (symptom, positive urinalysis or laboratory result) of a relapse of TMA and the re-introduction of eculizumab. Other secondary outcomes include; describing the relapse rate after withdrawal of eculizumab as determined by the proportion of participants who relapsed after eculizumab was withdrawn and relationship to defects in complement regulation, the period from withdrawal to relapse in those participants who restarted treatment, the change in estimated GFR over the course of the trial from baseline (day 0) to end of the trial and predictors of relapse.

### Statistical analysis

A recruitment maximum of 30 participants (28 recruited) was planned based on the analysis methodology. The Bayes factor single arm binary model was used to monitor and analyse the primary outcome data.^21^ From historical data the event rate for the standard of care is 0.06, based on six serious adverse events (SAE) judged to be either definitely or probably related to eculizumab treatment in the first 100 patients treated by the national aHUS Service (unpublished data). This was assumed to be the rate under the null hypothesis. Treatment withdrawal was expected to give a withdrawal-related event (primary outcome) rate of 0.12, assumed to be the rate under the alternative hypothesis. The acceptability of this was determined by patient and carer consultation during the trial development phase (Appendix p33). We assumed that the sample distribution of number of responses followed a binomial distribution and used an inverse moment prior for response under the alternative hypothesis. The following rules were then used to generate stopping boundaries: the trial was to be stopped for superiority (there being fewer serious events on the intervention than would be expected under standard of care) if the posterior probability of the alternative hypothesis was less than 0.05 or it was to be stopped for inferiority if the posterior probability of the alternative hypothesis was greater than 0.80. The trial was to be stopped for inferiority with two events in the first cohort of five participants. Subsequently, the trial would stop if three or more events were observed in the first 15 participants, four or more in the first 20 participants, and five or more in the whole trial population (Appendix p25–26). With the planned sample size and parameter choices a decision was made not to stop the trial for superiority.

The operating characteristics and stopping boundaries were produced using the M. D. Anderson Cancer Center Department of Biostatistics software BayesFactorBinary, version 1.0. If the true rate is 0.06 (Scenario 1, null hypothesis), the trial would stop with probabilities of 0.096 and 0 in favour of the alternative and null hypotheses, respectively. The average number of participants (10%, 90% percentiles) is 28.44 (30, 30). If the true rate was 0.12 (Scenario 2, alternative hypothesis), the trial would stop with probabilities of 0.443 and 0 in favour of the alternative and null hypotheses, respectively. The average number of participants (10%, 90%) was 23.48. 1000 repetitions were used in the software simulation. Calculations with different numbers of repetitions resulted in unchanged stopping boundaries with only marginal changes to the operating characteristics.

In addition to presenting the analysis above, data is also reported descriptively, together with the number of participants recruited. Due to the sample size, no comparative statistical methods are applied. There is no imputation of missing data.

### Role of the funding source

The funder of the trial had no role in trial design, data collection, data analysis, data interpretation, or writing of the report.

## Results

Of the 64 patients on eculizumab treatment at 15 participating sites 49 patients (77%) were suitable for treatment withdrawal based on the inclusion and exclusion criteria. During the trial 28 participants withdrew from treatment between 28/11/2018 and 12/01/2022 (Fig. 1). The baseline characteristics of participants are shown in Table 1. Fifteen (54%) of withdrawal participants were male, 64% of white ethnicity and 18 (64%) were children (<18 years). One participant had a working kidney transplant. 41% of participants required dialysis and 37% had extrarenal disease manifestations at the time of initial presentation. Seventeen withdrawal participants (61%) had either a genetic or acquired cause of complement dysregulation identified (Appendix p33). Participants had been on eculizumab for a median of 22.5 months (range 6–56 months) prior to withdrawal of treatment (Appendix p33).

**Table 1:** Baseline participant characteristics.

	N (%)
Sex	
Male	15 (54)
Female	13 (46)
Ethnicity	
White	18 (64)
Non-white	10 (36)
Dialysis dependent at presentation^a^	11 (41)
Neurological involvement at presentation^a^	4 (15)
Any other organ involvement at presentation^a^	6 (22)
Plasma exchange at presentation^a^	10 (37)
Renal transplant^a^	1 (4)
Complement genetic variant	
Complement Factor H	4 (14)
Complement C3	3 (11)
Complement Factor I	0
Complement Factor B	0
CD46	5 (18)
No pathogenic variant identified	11 (39)
FH autoantibodies	5 (18)
Haematuria	
Neg/trace	25 (89)
1+	3 (11)
Age in years at start of study^b^	
Mean (SD)	17.3 (15.2)
Median (IQR)	10.9 (6.2–29.0)
Range	2–59.3
Blood pressure:	
Systolic (mmHg)^a^	
Mean (SD)	111.6 (21.1)
Median (IQR)	109 (55–103)
Diastolic (mmHg)^a^	
Mean (SD)	69.3 (15.1)
Median (IQR)	70 (79–106)

a Missing data from one participant.

b 18/28 (64%) participants in trial age under 18 years (Children).

One participant experienced a primary outcome event (3.6%). This participant experienced a gradual decline in eGFR in the absence of microangiopathic haemolysis or thrombocytopaenia. A renal biopsy was not performed. In the absence of an alternative explanation for the decline in renal function, renal TMA was assumed and eculizumab restarted 74 weeks after treatment withdrawal (Table 2). Despite this, the participant's renal function did not recover with a persistent loss of eGFR >20% of baseline. Based on the decision rules described in the methods section, the trial demonstrates that the withdrawal of eculizumab is not inferior to staying on treatment in terms of the primary outcome.

**Table 2:** Summary of participants with disease relapse.

Participant id	Age (years)^a^	Gender	Time from withdrawal (weeks)	Complement gene variant	Details of relapse	Outcome
7	35	Male	19	*CFH* c.3570T>G p.(Tyr1190∗) Heterozygous	Relapse diagnosed on the basis of a fall in Hb and rise in Cr at a scheduled visit. Platelet count remained within the normal range (Appendix p69). No precipitant identified.	Eculizumab restarted, full recovery
8	37	Male	74	*CD46* c.97+2_97+12del Homozygous	Initially stable after withdrawal of eculizumab with an eGFR of >60 ml/min/1.73 m^2^. Gradual decline in eGFR to <50 ml/min/1.73 m^2^, proteinuria and hypertension in the absence of features of MAHA or thrombocytopaenia (Appendix p70). Kidney biopsy was not performed. Eculizumab was restarted but eGFR did not recover.	Primary outcome event
16	9	Male	26	CFH c.3643C>G (p.Arg1215Gly) Heterozygous	Participant had recently made a full recovery from streptococcal tonsillitis though with significant proteinuria, blood tests were suggestive of relapse (raised LDH and falling platelet count). Participant restarted eculizumab within 24 h at an unscheduled visit.	Eculizumab restarted, full recovery
19	6	Female	21	*C3* c.3470T>C (p.Ile1157Thr) Heterozygous	Presentation with presumed portacath infection and haematological features of TMA. Patient had eculizumab treatment restarted at an unscheduled visit within 24 h.	Eculizumab restarted, full recovery

a At time of eculizumab withdrawal.

A diagnosis of relapse was made in three other participants (one adult and two children) between 19 and 26 weeks following treatment withdrawal (Table 2). The adult participant also presented with a gradual decline in eGFR. Both children presented acutely with characteristic features of aHUS in the context of infection. The overall relapse rate during the 2-year follow up period was 14.3% (4/28, 95% CI 1–9). Relapses occurred in 2/18 children (11%) and 2/10 (20%) of adults. One relapse occurred every 14 years off treatment for the whole withdrawal cohort.

The four relapses occurred in participants with pathogenic variants in complement genes, two in *CFH*, one in *CD46*, and one in *C3*. The relapse rate was 66% (2/3), 20% (1/5), and 50% (1/2) in participants with *CFH*, *CD46*, and *C3* pathogenic variants respectively. Two participants had variants of uncertain significance (*CFH* and *C3*), neither of whom relapsed. No relapse occurred in participants in the absence of a genetic variant. No relapse occurred in the five participants whose initial disease was attributed to anti-FH autoantibodies despite significant autoantibody titres in four participants at the time of and after treatment withdrawal. No participant had received immunosuppression to reduce antibody titres (Appendix p33). Overall, the relapse rate was 24% (4/17) in participants with an abnormality in complement regulation; pathogenic variants (4/10), variants of uncertain significance (0/2) or anti-FH autoantibodies (0/5), corresponding to one relapse for every 8.5 years follow up. One participant had a documented increase from baseline in the level of non-visible haematuria at the scheduled visit immediately prior to restarting eculizumab treatment. During the trial 20 SAEs were reported (Supplementary Table S4).

The median participant eGFR remained stable throughout the two years follow up period. There was no increase in proteinuria based on measurement of urinary protein to creatinine ratio (uPCR). There was no evidence of an increase in microangiopathic haemolytic activity (based on fall in Hb or increase in LDH) or fall in platelet count (Table 3). A more detailed summary of renal function and all haematology and biochemical parameters are provided in Appendix p35–67.

**Table 3:** Biochemical and haematological parameters following withdrawal of eculizumab.

	Baseline	3 months	12 months	24 months
Cr (μmol/l)				
Mean (SD)	55.8 (34.5)	62.1 (50.3)	56.8 (27.7)	55.6 (23.6)
Median (IQR)	42 (29–76)	51 (32–81)	54 (37–66)	53 (35.5–77)
eGFR (ml/min)				
Mean (SD)	116.0 (37.2)	108.9 (33.3)	113.3 (34.2)	110.3 (29.2)
Median (IQR)	117.7 (90.9–128.5)	105.7 (84.1–129.3)	106.7 (91.7–125.9)	105.5 (86.0–123.0)
Hb (g/l)				
Mean (SD)	127.3 (15.3)	125.8 (14.5)	126.3 (11.1)	129.9 (13.4)
Median (IQR)	127 (115–140)	128 (118–138)	126 (116–135)	131.5 (118–136.5)
LDH (u/l)				
Mean (SD)	340.2 (144.2)	298 (112.0)	255.3 (118.0)	247.4 (66.9)
Median (IQR)	301 (217–494)	260 (205–377)	225 (178.5–276)	246 (215–268)
Platelets (×10^9^/l)				
Mean (SD)	282.7 (86.6)	301.8 (98.7)	279.8 (61.9)	283.4 (105.3)
Median (IQR)	267 (228–324)	297 (232–338)	271 (237–325)	269 (233–294)
uPCR (mg/mmol)				
Mean (SD)	35.1 (50.9)	30.5 (33.2)	38.6 (65.7)	30.6 (52.5)
Median (IQR)	17.2 (9.5–34)	15.2 (10.3–38.5)	15.1 (10.4–34)	16.3 (7.5–24)

As primary outcome data was only available on the 28 participants recruited and not the 30 participants originally planned, a sensitivity analysis was performed exploring the effect on trial conclusions assuming that the remaining two participants experienced a primary outcome event. If this had occurred, the above conclusion would remain unchanged based on three primary outcome events in 30 participants in the sensitivity analysis.

## Discussion

Using Bayes statistical methodology we show that, in terms of the trial primary outcome and the pre-specified analysis plan, withdrawal of eculizumab can be achieved without exposing patients to additional harm compared with continued treatment. This is the first report to use this type of methodology to statistically assess whether it is feasible and safe to withdraw eculizumab therapy in patients with aHUS and replace treatment with a monitoring schedule. Only one participant met the criteria for a primary outcome event with a fall in eGFR >20% which did not recover following the reintroduction of eculizumab. There were no haematological features of a TMA and biopsy was not performed. Although outside of the trial follow up period, monitoring of renal function of this participant showed that eGFR subsequently improved. This alternative strategy reduces the burden of treatment, risks associated with treatment, in particular meningococcal infection, and leads to a significant reduction in healthcare costs. These results, and other published experience, are likely to result in adoption of alternative strategies for treatment of aHUS.

This trial did not use relapse as the primary outcome measure, as the natural history of disease predicts that some patients will relapse following withdrawal of eculizumab. Critically, we were able to re-introduce treatment when a relapse was diagnosed for three participants with no evidence of harm as a consequence of the relapse. Therefore, we propose that for patients fulfilling the trial entry criteria, the treatment strategy can be changed from lifelong treatment to a limited duration of treatment (minimum 6 months, as defined in the trial protocol), with a monitoring of disease activity replacing treatment. The trial included patients on dialysis or with extra-renal manifestations of disease at presentation, but excluded some higher risk groups (transplant patients with higher risk genetic variants and patients with CKD stage 4–5, uncontrolled hypertension or unstable renal function). This current trial did not consider withdrawal again after treatment reintroduction and more data is required to inform how this patient group is managed in the future. In addition, the trial was conducted through a national co-ordinating centre with complete genotyping and autoantibody screening performed on all patients with immediate access to eculizumab if required. Therefore, implementation of trial results will need to consider the resources available and this will apply only to patients fulfilling the entry criteria for trial entry. Based on these criteria, approximately 75% of patients on eculizumab treatment in the UK would be eligible to withdraw from treatment. Although we do not know the patient population at non-participating sites is similar, we have no reason to think otherwise.

The risk of relapse can be stratified by the presence of a defect in complement regulation.^14^ Four out of 17 participants (24%) with a defect in complement regulation relapsed whereas no relapse was seen in participants with no complement regulatory defect. This is consistent with other published reports, with relapse in the absence of an identifiable cause of complement dysregulation being very uncommon occurring in only two of 71 patients in published series.^14^, ^15^, ^16^^,^^22^, ^23^, ^24^ A pathogenic or likely pathogenic variant in a complement regulatory gene is associated with the highest risk with a relapse occurring in 44% of patients (Table 4). The nature of the complement regulatory defect may also be important in predicting the likelihood of relapse. In the SETS aHUS trial two out of three participants with pathogenic variants in *CFH* and one of two participants with a pathogenic variant in *C3* experienced a relapse. Combining all published reports, pathogenic or likely pathogenic variants in *CFH* confer the highest risk of relapse (60%). For pathogenic variants in other complement regulatory genes the risk of relapse is 25–43%.^14^, ^15^, ^16^^,^^22^, ^23^, ^24^ The number of patients reported with a variant of uncertain significance is low, but evidently these variants do confer a risk of relapse.^14^^,^^22^ As these results are used to develop withdrawal protocols data on relapse risk will have implications for monitoring after withdrawal. It may be that the intensity of monitoring can be modified according to the risk of relapse, with patients with pathogenic variants in *CFH* requiring more intensive monitoring similar to that used in the trial, whilst other groups can have less frequent monitoring. This is also relevant to counselling of patients prior to treatment withdrawal. However, even combining the published data, patient numbers are small and collection of outcome data following withdrawal, ideally through national registries, will be critical.

**Table 4:** Summary of relapse rates in published series.

	Median duration of follow up (months)	Patients with genotyping/antibody status	Relapse (%)	P/LP variant	Relapse (%)	VUS	Relapse (%)	Anti-FH antibodies	Relapse (%)	No variant or antibody	Relapse (%)
SETS aHUS trial	24	28	4 (14)	10	4 (40)	2	0	5	0	11	0
Bouwmeester et al., 2022^24^	18.8^a^	18	4 (22)	13	4 (31)	2	0	2	0	4	0
Fakhouri et al., 2021^23^	19.8	55	13 (24)	26	10 (38)	1	1	4	0	23	1^b^ (4)
Merrill et al., 2017^16^	11	13	3 (23)	2	2 (100%)	3	0	0	0	8	1^c^ (13)
Wijnsma et al., 2018^22^	Not reported	18	5 (28)	9	3 (33)	4	2	1	0	4	0
Fakhouri et al., 2017^14^	22	38	12 (32)	18	10 (56)	3	2	1	0	16	0
Ardissino et al., 2015^15^^,^^25^	13.1	16	3 (19)	5	3 (60)	3	0	3^d^	1	5	0
Baskin et al., 2022^26^	50	18	4	0	0	0	0	0	0	18	4
Total		204	48 (24)	83	36 (44)	16	5 (31)	16	1 (6)	89	6 (7)

P/LP—pathogenic/likely pathogenic; VUS—variant of uncertain significance.

a Excluding patients who did not recover kidney function.

b Subsequently found to have hereditary ADAMTS13 deficiency.

c Liver transplant recipient with treatment non-adherence.

d Excluding a patient with anti-FH autoantibodies who also had a variant in *CFH*.

Previous reports have suggested that self-monitoring for non-visible haematuria (or haemoglobinuria) is useful to detect relapse.^15^^,^^25^ In this trial we did not reproduce this finding. Only one participant had a change in haematuria/haemoglobinuria in the scheduled visit prior to relapse and, in the two participants in whom it was recorded, neither had a change in haematuria/haemoglobinuria at the unscheduled visit when eculizumab was restarted. Although the development of haematuria/haemoglobinuria may associate with relapse in some patients this may not be the case in renal-limited TMA recurrence when haematological features are mild or absent. Therefore, haematuria/haemoglobinuria does not appear to be a reliable biomarker of relapse and needs to be used with caution. A negative result may result in false reassurance and delay in re-initiation of treatment.

It was also evident that relapse does not necessarily follow the characteristic, acute course with AKI, thrombocytopaenia and microangiopathic haemolysis. This was the presentation in the two children in whom an acute relapse occurred, diagnosed at an unscheduled visit. However, in the two adults, relapse presented as a gradual decline in renal function, initially without haematological features of a TMA. This highlights the potential challenges in applying pre-defined objective criteria to diagnose relapse and has implications for future monitoring. It will be vital to inform patients to self-present in the event of any acute, unexplained illness and to have infrastructure to support this. It will also be important to have long-term, routine monitoring in place to detect any gradual loss of renal function, however, the frequency of this may be less than in the trial. In addition to the forthcoming economic evaluation based upon the data collected within this study, longer-term follow up studies are required to determine whether patients at low risk of relapse, particularly those with no identifiable cause for complement dysregulation, can be safely discharged from routine follow up.

Unlike other descriptive cohort studies, this trial used statistical methodology to investigate the safety of stopping eculizumab treatment in aHUS. This trial has the limitation that it only has a single arm with only 28 participants in total and 4 with pathogenic variants in *CFH* withdrawing from treatment.

However, it would not be possible to undertake a randomised control trial in this patient cohort due to the rarity of aHUS. Indeed, no randomised trial has been conducted in this disease, this includes the single arm, open label trials that led to approval of eculizumab and subsequently ravulizumab for the treatment of aHUS.^7^^,^^27^ This trial demonstrates the application of Bayesian methodology to interventional trials in rare diseases and could be an exemplar to inform the design of future studies of interventions in rare diseases when a two-arm trial is not feasible. This is particularly the case as cohorts of rare disease patients, with granular data on disease phenotype and outcome required for this type of analysis, become available.^28^

## Contributors

NSS, VB, SJ, DK, MM, EM, CM, YO, JL, TC, and EW contributed to design of the trial. All authors contributed to data collection. NSS, AB, TC, JL, YO, GO, and LV were responsible for data analysis. CW, AB, TC, CK, SD, SC, and NSS had access to and verified the underlying data reported in the manuscript. All authors all had full access to all the data in the trial and had final responsibility for the decision to submit for publication. NSS prepared the first draft and all authors contributed to revision of the manuscript and approved the final version.

## Data sharing statement

De-identified individual participant data that underlie the reported results will be made available for approved use by the trial authors. Proposals for access should be sent to neil.sheerin@newcastle.ac.uk. Data will also be submitted to the NIHR library.

## Declaration of interests

NSS has contributed to advisory boards and committees for Alexion pharmaceuticals, Novartis, Roche, and Astra Zeneca and received honoraria from Alexion Pharmaceuticals. DK has received consultancy fees from Gyroscope Therapeutics, received support to attend meetings from Alexion pharmaceuticals and contributed to committees for Novartis, Alexion Pharmaceuticals, and Silence Therapeutics. EM has received support to attend meetings from Alexion Pharmaceuticals. SJ has received honoraria from Novartis and Alexion Pharmaceuticals and contributed to advisory boards for Alexion Pharmaceuticals. EW has received consultancy fees from Apellis, Biocryst, Novartis, Arrowhead, and Sobi, received honoraria from Novartis and Alexion Pharmaceuticals and participated in advisory boards for Novartis. MM has received consultancy fees from Alexion Pharmaceuticals and Novartis and received honoraria and travel support from Alexion Pharmaceuticals. VB was funded by a Medical Research Council/Kidney Research UK training fellowship. LV, GO, SD, JL, TC, AB, SC, SD, CK, YO, CW, VB, and CM have no conflicting interests.

## Appendix A Supplementary data

SETS aHUS - Protocol

Appendix

SETS aHUS Investigator Group
