# Supplementary material for: Eculizumab withdrawal and monitoring in atypical haemolytic uraemic syndrome (SETS aHUS): a multicentre, open label, prospective, single arm trial
Source: Lancet Reg Health Eur. 2025 Aug 7;56:101392. doi: 10.1016/j.lanepe.2025.101392 (PMC12811780; doi:10.1016/j.lanepe.2025.101392)
Supplement: SETS aHUS - Protocol [file mmc1.pdf]

# SETS aHUS

| | |
|---|---|
| **Full Title:** | Multicentre, open label, prospective, single arm study of the safety and impact of eculizumab withdrawal in patients with atypical haemolytic uraemic syndrome |
| **Short Title/Acronym:** | **SETS aHUS**: **S**topping **E**culizumab **T**reatment **S**afely in **aHUS** |
| **Protocol Version Number & Date:** | **10.0 21 November 2023** |

Statement:

This protocol has regard for the HRA guidance.

# RESEARCH REFERENCE NUMBERS

| | |
|---|---|
| **IRAS Number:** | **235552** |
| **EudraCT Number:** | **2017-003916-37** |
| **NHS REC Reference:** | **18/NE/0078** |
| **UKCRN Portfolio Reference:** | **CPMS 36901** |
| **ISRCTN Number:** | **ISRCTN17503205** |

# RESEARCH SPONSOR

| | |
|---|---|
| **Sponsor Name:** | **Newcastle Upon Tyne Hospitals NHS Foundation Trust** |
| **Sponsor Reference:** | **08448** |

# RESEARCH FUNDER(S)

| | |
|---|---|
| **Funder Name:** | **NIHR HTA** |
| **Funder Reference:** | **15/130/94** |

# SIGNATURE PAGE

The undersigned confirm that the following protocol has been agreed and accepted. The Chief Investigator agrees to conduct the trial in compliance with the approved protocol and will adhere to the principles outlined in the Medicines for Human Use (Clinical Trials) Regulations 2004 (SI 2004/1031), amended regulations (SI 2006/1928) and any subsequent amendments of the clinical trial regulations, Good Clinical Practice (GCP) guidelines, the relevant Standard Operating Procedures and other regulatory requirements as amended.

I agree to ensure that the confidential information contained in this document will not be used for any other purpose other than the evaluation or conduct of the clinical investigation without the prior written consent of the Sponsor.

I also confirm that I will make the findings of the trial publicly available through publication or other dissemination tools without any unnecessary delay and that an honest accurate and transparent account of the trial will be given; and that any discrepancies and serious breaches of GCP from the trial as planned in this protocol will be explained.

**Representative of the Research Sponsor**

**Name:** **Sean Scott**

**(print)**

**Position:** **Regulatory Compliance Manager**

**Signature:** Sean Scott **Date:**

Sean Scott (Nov 7, 2024 17:16 GMT)

**Chief Investigator**

**Name:** **Professor Neil Sheerin**

**(print)**

**Signature:** Neil Sheerin **Date:**

Neil Sheerin (Sep 23, 2024 13:20 GMT+1)

**Senior Statistician**

**Name:** **Thomas Chadwick**

**(print)**

**Position:** **Senior Trial Statistician**

**Signature:** T Chadwick
T Chadwick (Sep 23, 2024 16:46 GMT+1)

**Date:** 23/09/2024

---

**Newcastle Clinical Trials Unit (NCTU)**

**Name:** Ciara A Kennedy
**(print)**
**Position:** Trial Manager

**Signature:** Ciara Kennedy
Ciara Kennedy (Sep 20, 2024 10:33 GMT+1)

**Date:** 20/09/2024

# PROTOCOL ACCEPTANCE SIGNATURE PAGE

**Short Trial Title: SETS aHUS**: **S**topping **E**culizumab **T**reatment **S**afely in **aHUS**

**Principal Investigator**

I have carefully read and understood protocol version 10.0. dated 21 Nov 2023 I agree to conduct the trial in compliance with Good Clinical Practice and all required regulatory requirements.

Name:

Position:

Signature: Date:

# KEY TRIAL CONTACTS

| | |
|---|---|
| **Chief Investigator** | Professor Neil Sheerin<br>Professor Of Nephrology<br>4th Floor William Leech Building<br>Medical School<br>Newcastle University<br>Newcastle upon Tyne<br>NE2 4HH<br>0191 222 7146<br>Neil.Sheerin@newcastle.ac.uk |
| **Trial Manager** | Dr Ciara Kennedy<br>Trial Manager<br>Newcastle Clinical Trials Unit<br>1-4 Claremont Terrace<br>Newcastle upon Tyne<br>NE2 4AE<br>Tel: 0191 208 2514<br>Ciara.Kennedy1@newcastle.ac.uk |
| **Senior Trial Manager** | Ms Sarah Dunn<br>Senior Trial Manager<br>Newcastle Clinical Trials Unit<br>1-4 Claremont Terrace<br>Newcastle upon Tyne<br>NE2 4AE<br>Tel: 0191 208 2521<br>Sarah.Dunn2@newcastle.ac.uk |
| **Sponsor** | The Newcastle upon Tyne Hospitals NHS Foundation Trust<br>Ms Laura Frisby<br>Level 1, Regent Point<br>Regent Farm Road<br>Newcastle upon Tyne<br>NE3 3HD<br>Tel: 0191 282 5959<br>tnu-tr.sponsormanagement@nhs.net |
| **Funder(s)** | NIHR Health Technology Assessment Programme<br>National Institute for Health Research<br>Evaluation, Trials and Studies Coordinating Centre<br>University of Southampton<br>Alpha House, Enterprise Road<br>Southampton<br>SO16 7NS |

**Collaborator/Co-Investigators**

Professor David Kavanagh
National Renal Complement Therapeutics Centre
aHUS service
Building 26, Royal Victoria Infirmary, Queen Victoria Road
Newcastle upon Tyne, NE1 4LP
Tel +44 (0) 191 282 5094
david.kavanagh@ncl.ac.uk

Dr Sally Johnson
Institute of Cellular Medicine,
Newcastle University,
4th Floor, William Leech Building,
Medical School, Framlington Place,
Newcastle upon Tyne, NE2 4HH
Tel: +44 (0) 191 208 5851
sally.johnson2@ncl.ac.uk

Mr Len Woodward
8 Meadow Drive
Knutsford
Cheshire, WA16 0DT
Telephone 01565 651693
len.woodward@ntlworld.com

Ms Jan Lecouturier
Senior Research Associate
Institute of Health & Society
Baddiley-Clark Building
Richardson Road
Newcastle upon Tyne, NE2 4AX
Tel: +44 (0) 191 208 562 9
jan.lecouturier@ncl.ac.uk

Dr Thomas Chadwick
Clinical Trials Statistician
Institute of Health and Society
Baddiley Clark Building
Richardson Road
Newcastle University
Newcastle upon Tyne, NE2 4AX
Tel: +44 (0) 191 208 6039
thomas.chadwick@ncl.ac.uk

Dr Yemi Oluboyede
Senior Research Associate
Baddiley-Clark Building
Richardson Road
Newcastle upon Tyne
NE2 4AA
Tel: +44 (0) 191 208 3741
yemi.oluboyede@ncl.ac.uk

| | |
|---|---|
| **Committees** | Chair of DMC<br>Professor Phil Kalra<br>Nephrologist<br>Dept Renal Medicine<br>Salford Royal Hospital<br>Stott Lane<br>Salford<br>M6 8HD<br>0161 206 0509<br>philip.kalra@srft.nhs.uk<br><br>Chair of TSC<br>Professor Jeremy Hughes<br>Nephrologist<br>MRC Centre for Inflammation Research<br>The University of Edinburgh<br>The Queen's Medical Research Institute<br>47 Little France Crescent<br>Edinburgh<br>EH16 4TJ<br>0131 242 9167<br>Jeremy.Hughes@ed.ac.uk |
| **Trial Website** | http://www.atypicalhus.co.uk/ |

# TRIAL SUMMARY

| | |
|---|---|
| **Trial Title** | Stopping Eculizumab Treatment Safely in atypical Haemolytic Uraemic Syndrome (aHUS) |
| **Acronym** | SETS aHUS |
| **Clinical Phase** | Phase IIb |
| **Summary of Trial Design** | Single arm, non-blinded assessment of the safety of Eculizumab withdrawal in patients with aHUS with continuous monitoring of adverse events using the Bayes factor single arm design. |
| **Summary of Participant Population** | Patients with atypical Haemolytic Uraemic Syndrome who are receiving Eculizumab therapy |
| **Planned Sample Size** | 30 patients who withdraw from Eculizumab<br><br>20 patients who will remain on treatment (completing Health Economic assessments only) |
| **Planned Number of Sites** | 20 |
| **Treatment withdrawal Duration** | 24 months |
| **Follow Up Duration** | 24 months |
| **Planned Trial Period** | 48 months |
| | **Objectives** |
| **Primary** | To determine the safety of Eculizumab withdrawal in patients with aHUS |
| **Secondary** | 1. Measure the effectiveness of a monitoring protocol to detect disease relapse following withdrawal of Eculizumab.<br>2. Describe the relapse rate after withdrawal of Eculizumab.<br>3. Estimate the proportion of patients, currently on long-term treatment with Eculizumab, who can be maintained off treatment.<br>4. Describe the period from withdrawal to relapse in those patients who restart treatment.<br>5. Measure the change in estimated Glomerular Filtration rate (GFR) over the course of the study.<br>6. Identify important clinical and laboratory indicators of imminent relapse.<br>7. To assess the costs and health outcomes (measured in terms of adverse events and quality-adjusted life years (QALYs)) for patients on standard care (not withdrawing from Eculizumab treatment) over the two-year trial duration. |

| | |
|---|---|
| | 8. To assess the costs and health outcomes for patients fully, or partially, withdrawing from Eculizumab treatment, and on a policy of protocolised monitoring, over the two-year trial duration.<br>*9.* To model the costs and health consequences (measured in terms of QALYs) associated with Eculizumab withdrawal, and a policy of protocolised monitoring following withdrawal (and treatment re-introduction if necessary), compared with standard care, beyond the two-year timeframe of the trial. |
| **Withdrawn Medicinal Product** | Eculizumab (Trade name - Solaris) |

# Contents


RESEARCH REFERENCE NUMBERS ..... 2
SIGNATURE PAGE ..... 3
PROTOCOL ACCEPTANCE SIGNATURE PAGE ..... 5
KEY TRIAL CONTACTS ..... 6
TRIAL SUMMARY ..... 9
GLOSSARY OF ABREVIATIONS ..... 14
1. BACKGROUND ..... 16
2. RATIONALE ..... 18
2.1. Risk Assessment ..... 19
3. OBJECTIVES AND OUTCOME MEASURES ..... 20
3.1. Primary Objective ..... 20
3.2. Secondary Objectives ..... 20
3.3. Primary Outcome Measures ..... 20
3.4. Secondary Outcome Measures ..... 21
4. TRIAL DESIGN ..... 23
5. STUDY SETTING ..... 23
6. ELIGIBILITY CRITERIA ..... 23
6.1. Inclusion Criteria ..... 23
6.2. Exclusion Criteria ..... 25
7. TRIAL PROCEDURES ..... 25
7.1. Recruitment ..... 25
7.1.1 Patient Identification and Central Pre-Screening ..... 25
7.1.2. Site Screening (day -28 to -14) ..... 26
7.1.3. Consent ..... 26
7.1.4. Final Eculizumab Infusion (Visit 1, Day -14) ..... 27
7.2.1. Baseline Assessments & Data (Visit 2, Day 0 +/- 2 days) ..... 27
7.2.2. Study Visits (Visits 3-34) ..... 27
7.2.3. Self-monitored Urinalysis ..... 29
7.2.4. Change in Health Status ..... 31
7.3 Disease Relapse ..... 31
7.3.1 Definition of disease relapse ..... 31
7.3.2 Management of a Relapse ..... 32

7.3.2.1. Reporting a relapse ..... 32
7.3.2.2. Restarting treatment following relapse ..... 32
7.4 Schedule of Events for withdrawal participants ..... 34
7.5 Schedule of Events for Health Economics participants ..... 36
7.6 Trial Withdrawal Criteria ..... 37
7.7 Storage and Analysis of Samples ..... 37
7.8 End of Trial ..... 38
7.9 COVID-19 Pandemic ..... 38
8 . HEALTH ECONOMICS ..... 39
8.1. Health economic data collection ..... 39
8.1.1. Assessment of cost ..... 39
8.1.2. Assessment of health-related quality of life ..... 40
8.1.3. Economic analyses ..... 40
9 WITHDRAWN MEDICATION ..... 41
9.1 Name and Description of withdrawn Medication ..... 41
9.1.1 Eculizumab ..... 41
9.1.2 Access to Eculizumab ..... 41
9.2 Relapse Information ..... 41
9.3 Dosage Schedule ..... 42
9.4 Concomitant Medications ..... 42
10 . PHARMACOVIGILANCE ..... 43
10.1 Definitions ..... 43
10.2 Recording and Reporting AEs and SAEs ..... 44
10.3 Recording and Reporting SUSARs ..... 44
10.4 Responsibilities ..... 45
10.5 Notification of Deaths ..... 46
10.6 Pregnancy Reporting ..... 46
10.7 Reporting Urgent Safety Measures ..... 46
10.8 Development Safety Update Reports ..... 46
11 STATISTICAL CONSIDERATIONS ..... 47
11.1 Analysis Population ..... 47
11.2 Statistical Analyses ..... 47
11.2.1 Analysis of the Primary Outcome Measure ..... 47
11.2.2. Analysis of Secondary Outcome Measures ..... 49

11.2.3. Subgroup Analyses ... 49
11.3. Health economic data analysis ... 50
11.3.1. Analysis Population ... 50
11.3.2. Within-trial assessments of costs and outcomes ... 50
11.3.3. Assessment of cost-effectiveness ... 50
11.4. Sample Size Calculations ... 51
12. DATA HANDLING ... 51
12.1. Data Collection Tools and Source Document Identification ... 51
12.2. Data Handling and Record Keeping ... 51
12.3. Access to Data ... 52
12.4. Archiving ... 52
13. MONITORING, AUDIT & INSPECTION ... 52
14. ETHICAL AND REGULATORY CONSIDERATIONS ... 53
14.1. Research Ethics Committee Review and Reports ... 53
14.2. Peer Review ... 54
14.3. Public and Patient Involvement ... 54
14.4. Regulatory Compliance ... 54
14.5. Protocol Compliance ... 55
14.6. Notification of Serious Breaches to GCP and/or the Protocol ... 55
14.7. Data Protection and Patient Confidentiality ... 55
14.8. Indemnity ... 55
14.9. Amendments ... 56
14.10. Post-Trial Care ... 56
14.11. Access to the Final Trial Dataset ... 56
15. DISSEMINATION POLICY ... 56
16. REFERENCES ... 57
17. APPENDICES ... 59
17.1 Appendix 1 - Safety Reporting Diagram ... 59
17.2. Appendix 2 – Amendment History ... 60

# GLOSSARY OF ABREVIATIONS

| ABBREVIATION | DEFINITION |
|---|---|
| AE | Adverse Event |
| aHUS | Atypical Haemolytic Uraemic syndrome |
| AKI | Acute Kidney Injury |
| AR | Adverse Reaction |
| CDMS | Clinical Data Management System |
| CI | Chief Investigator |
| CKD | Chronic Kidney Disease |
| CTA | Clinical Trial Authorisation |
| CTIMP | Clinical Trial of an Investigational Medicinal Product |
| DMC | Data Monitoring Committee |
| DSUR | Development Safety Update Report |
| eCRF | electronic Case Report Form |
| GCP | Good Clinical Practice |
| GFR | Glomerular Filtration rate |
| HRA | Health Research Authority |
| HTA | Human Tissue Authority |
| ICF | Informed Consent Form |
| ICH | International Conference on Harmonisation of technical requirements for registration of pharmaceuticals for human use |
| IMP | Investigational Medicinal Product |
| ISF | Investigator Site File |
| LDH | Lactate Dehydrogenase |
| MA | Marketing Authorisation |
| MAHA | Microangiopathic haemolytic anaemia |

| | |
|---|---|
| MHRA | Medicines and Healthcare products Regulatory Agency |
| NCTU | Newcastle Clinical Trials Unit |
| NHS | National Health Service |
| NHSE | National Health Service England |
| NICE | National Institute for Health and Care Excellence |
| NRCTC | National Renal Complement Therapeutics Centre |
| PSS | Personal Social Services |
| PI | Principal Investigator |
| PIS | Participant Information Sheet |
| QA | Quality Assurance |
| QALY | Quality Adjusted Life Year |
| QC | Quality Control |
| R&D | Research & Development |
| REC | Research Ethics Committee |
| RSI | Reference Safety Information |
| SAE | Serious Adverse Event |
| SAR | Serious Adverse Reaction |
| SDV | Source Data Verification |
| SOP | Standard Operating Procedure |
| SmPC | Summary of Product Characteristics |
| SUSAR | Suspected Unexpected Serious Adverse Reaction |
| TMA | Thrombotic Microangiopathy |
| TMG | Trial Management Group |
| TSC | Trial Steering Committee |
| TMF | Trial Master File |

# 1. BACKGROUND

*Atypical Haemolytic Uraemic Syndrome (aHUS)*

aHUS is a severe, life-threatening disease characterised by thrombocytopenia, haemolytic anaemia and Acute Kidney Injury (AKI), although other organ involvement is common. Historically it has been associated with a poor prognosis, with 50% of patients developing end stage renal disease or dying in the first year after presentation (5). There is also a high risk of disease recurrence and graft loss after kidney transplantation (6). Prior to 2011 treatment options were limited and relied on plasma based therapies, either infusion or exchange, but in many cases this treatment failed to influence the course of disease (5) and was itself associated with significant morbidity and mortality (7). In the UK the incidence of aHUS is 0.4-0.5 cases per million per year (8).

A major breakthrough in the diagnosis and treatment of aHUS came with the finding that the disease can be caused by excessive activation of the complement system. Complement is an integral part of the body's immune system, the activity of which, in physiological conditions, is tightly regulated by a series of soluble and cell surface regulatory proteins.

Failure of regulation leads to excessive, inappropriate activation of complement, endothelial injury, activation of the coagulation cascade and a Thrombotic Microangiopathy (TMA). The clinical manifestations of the disease depend on the organs affected by the TMA, which most commonly involve the kidneys.

The association between aHUS and an inherited defect in a complement regulatory protein was first reported 20 years ago (9). In 60% of patients, an inherited genetic variant increasing complement activation can be identified and an acquired defect (autoantibody) is identified in a further 10% (10). There is incomplete penetrance with approximately 50% of people who carry a pathogenic genetic variant developing disease. It is accepted that a second, environmental trigger is required for disease to develop. Non-complement genetic causes can be identified in a small number of the remaining patients, but the cause of aHUS remains unidentified in a significant minority of patients. The underlying genetic variant that predisposes to disease has an influence on the severity of disease (6) and the likelihood of recurrent disease developing after transplantation (11).

*Eculizumab treatment for aHUS*

Eculizumab is a humanized monoclonal antibody that binds to, prevents the cleavage of and therefore inhibits the function of C5, an important protein involved in complement activation. By blocking C5, many of the effector functions of complement that are responsible for tissue damage are inhibited. Initially, case reports suggested that Eculizumab was effective in the treatment of aHUS (12). Two subsequent uncontrolled, open label trials involving 36 adult and adolescent patients confirmed the efficacy of Eculizumab treatment for aHUS over a 26 week period (13). Eculizumab was efficacious for the treatment of incident patients and those patients maintained in remission using continuous plasma-based therapies. An additional prospective study in children and a third study have confirmed efficacy (data presented in abstract form only). Follow-up of the original cohort suggests that treatment for 2 years is associated with good clinical outcomes (14). In these studies, identification of a complement genetic variant or acquired inhibitor did not influence the response to Eculizumab treatment. However, it is recognised that those patients with non-complement pathogenic genetic variants (e.g. DGKepsilon mutations) will not respond to treatment. On the basis of this evidence, Eculizumab was licensed for the treatment of aHUS by the European Medicines Agency (EMA) (15) and U.S. Food & Drug Administration (FDA) (16) in 2011. An initial evaluation by the Advisory Group for National Specialised Services recommended the use of Eculizumab in aHUS. However, this was subsequently referred to National Institute for Health and Care Excellence (NICE) for further evaluation. In the interim period, National Health Service England (NHSE) agreed to fund the use of Eculizumab, with coordination of treatment by an expert centre based in Newcastle upon Tyne. NICE

published its evaluation in 2015, recommending that Eculizumab should be used for the treatment of aHUS (3). Again, they recommended coordination of diagnostics and treatment in a specialist centre, which, after a tendering process through NHSE, was awarded to Newcastle upon Tyne (National Renal Complement Therapeutics Centre (NRCTC)). A second recommendation in the NICE evaluation was that funding was on the condition that there was a 'research programme with robust methods to evaluate when stopping treatment or dose adjustment might occur'.

*Risks associated with Eculizumab treatment*

It is clear from the trials that Eculizumab is relatively safe, and this is supported by data from patients with paroxysmal nocturnal haemoglobinuria in whom Eculizumab has been used or over 10 years. Complement is part of the immune system and, therefore, treatment with Eculizumab is immunosuppressive. In particular, complement blockade with Eculizumab predisposes to *Neisseria meningitidis* infection, the risk of which increased by 5000-fold in patients who are on Eculizumab treatment compared to the general population (17). All patients are vaccinated against meningococcal infection before starting Eculizumab and in the UK, the aHUS National Service recommends continuous prophylactic antibiotics, which itself carries the risk of emergent antibiotic resistance (currently up to 20% of *Neisseria meningitidis* in some areas). Despite these recommendations, there have been 4 cases of meningococcal infection in the UK in patients on Eculizumab treatment for aHUS, due to either antibiotic resistant strains (1 patient) or the patient not being on antibiotics (3 patients). There are concerns about the effectiveness of vaccination and the potency of anti-meningococcal antibodies in patients on Eculizumab because of lack of bactericidal complement activity. In addition, there have been two unexplained deaths (no cause found at post mortem) of patients on Eculizumab. Both deaths occurred in patients who had received a kidney transplant and were on standard post-transplant immunosuppression in addition to Eculizumab. This highlights the potential interaction between transplant immunosuppression and Eculizumab and the limited experience of this combination of therapies. We are also aware of uncommon infections occurring in this group (enteroviral pneumonitis and *Herpes simplex* meningitis) but whether these are attributable to Eculizumab treatment is unclear.

*Experience of Eculizumab withdrawal*

Lifelong treatment with Eculizumab is currently recommended because of the risk of relapse based on the known natural history of aHUS. There also have been reports of patients who have relapsed after the withdrawal of treatment (18).
However, many physicians are now questioning the recommendation for indefinite treatment and feel that alternative strategies for Eculizumab use should be considered. This is based upon the following:

- The risks associated with continued Eculizumab use as discussed above. In addition, there is the burden of treatment on patients and the financial impact on healthcare providers;
- From data prior to the introduction of Eculizumab, it is clear that the risk of relapse is greatest in the period immediately after first presentation. In the first year after presentation, 25% of children and 29% of adults will experience a relapse. 82% of relapses in adults, and 57% of relapses in children, occur in the first year after disease onset. Beyond the first year, only a further 20% of patients will relapse in the subsequent 5-10 years (5).
- Therefore, 50% of patients will not relapse after their initial presentation and would therefore, according to current recommendations, be on Eculizumab unnecessarily. We have used this data to estimate the cost savings to the NHS if 50% of patients can successfully withdraw from treatment;
- It is possible to predict, although not absolutely, which patients are more likely to relapse. Patients with no mutation are at a relatively low risk of relapse. Those patients with pathogenic variants in the gene encoding Membrane Cofactor Protein (CD46) are at high risk

of relapse, but have a relatively mild form of the disease. Therefore, continued monitoring after withdrawal is required as it is impossible to predict definitively that relapse will not occur in any individual patient.

The limited available evidence suggests that Eculizumab can be withdrawn in a significant proportion of patients. In addition, with monitoring for relapse and early reintroduction of treatment, complications from relapse can be avoided. No study has yet explored patients and carers views on how treatment and monitoring of disease can be delivered most effectively.

We are aware of a number of patients in the UK who have chosen to withdraw from treatment. A minority of patients have subsequently restarted treatment. The remaining patients continue to be well and relapse-free off treatment. A case series from Italy reported withdrawal of Eculizumab in 10 patients with aHUS with a cumulative follow up of 95 patient-months. Only 3 patients relapsed during this period (2). A recent update, now including 16, mainly paediatric, patients with a cumulative follow up of 243 patient-months reported 5 relapses. Relapse occurred early and treatment was reintroduced with no evidence of renal injury (1). This equates to 1 relapse every 49 months off treatment, with 70% (11/16 of patients) remaining stable off treatment. A similar experience has been reported in other publications (19).

# 2. RATIONALE

*Lack of evidence base*
The recommendation that Eculizumab treatment is continued lifelong is not evidence based. Data prior to the availability of Eculizumab suggests that if patients survive the initial episode of aHUS with intact renal function, less than 50% of patients will subsequently relapse. Therefore, over half of patients receiving maintenance Eculizumab treatment could be doing so unnecessarily.

*Burden of treatment*
Withdrawal of drug would lead to a reduction in the burden of treatment (intravenous Eculizumab injections every 2 weeks and use of prophylactic antibiotics), replacing it with reduced monitoring after a period of more intense monitoring. Patients will also avoid the potentially fatal complications associated with Eculizumab use (6 of approximately 100 patients treated have suffered severe complications – death, meningococcal or other unexpected, severe infection). There would be cost savings for the NHS of over £10.6 million if 50% of study participants withdraw for the 2 years of the study. These patients are likely to remain off treatment and the surveillance methods tested in this study will allow further patients to withdraw from treatment.

*Risk of unsupervised withdrawal*
Analysis in 2015 identified 10 patients in the UK with native renal function who have withdrawn from Eculizumab because of patient/physician choice. One patient has required re-initiation of treatment due to relapse. Relapse may occur after withdrawal of treatment but early re-initiation of treatment should avoid harm to patients. Therefore, a system of surveillance is required to detect relapse early, re-initiate treatment and avoid irreversible organ damage. Uncoordinated withdrawal of Eculizumab, without systematic monitoring, may place patients at risk of undiagnosed relapse and delayed reintroduction of treatment.
As the number of patients on Eculizumab increases, so does potentially unnecessary treatment and cost to the NHS (and the families themselves given the burden of using care). Therefore, a tested

method of safe withdrawal and monitoring will be vital for efficient delivery of this high cost treatment.

We feel that there is a clear rationale for a trial of Eculizumab withdrawal due to the following:

- The recommendation that Eculizumab treatment should be lifelong is not evidence based and probably unnecessary for at least 50% of patients.
- There is a widely acknowledged need for better evidence to inform the optimal use of this high cost treatment. If successful, this study will provide evidence for an alternative strategy for treatment of aHUS based on monitoring and treatment re-introduction rather than continuous treatment.
- There is a need to assess the efficacy of a monitoring protocol to detect early relapse of disease after withdrawal of treatment, particularly as some patients are independently choosing to withdraw from the drug.
- Understanding patients and carers' attitude to treatment withdrawal and acceptable levels of monitoring is critical to the successful implementation of the findings of this study.

There are a number of questions that will be addressed in this study:

- Can Eculizumab withdrawal, substituting treatment with a monitoring regime and treatment reintroduction in the event of relapse, be an effective, safe strategy for management of patients with aHUS?
- What costs and outcomes are associated with usual care compared with Eculizumab withdrawal?
- Is a policy of protocolised surveillance and treatment reintroduction, in the event of relapse, following withdrawal of Eculizumab cost-effective compared with the current standard of care?

**Overall aim**: To establish an evidence base for an alternative treatment strategy for patients with aHUS that includes the use of Eculizumab to establish remission, safe withdrawal of treatment and the reintroduction of Eculizumab in those patients who relapse.

## 2.1. Risk Assessment

This study involves the withdrawal of a treatment that is currently standard medical care. Eculizumab is licenced for the treatment of aHUS and indefinite treatment is recommended once treatment has been started. However, treatment with Eculizumab is associated with significant side effects, in particular a risk of serious infection. There has been no formal assessment comparing the risk of continuing treatment with withdrawal and reintroduction of treatment if relapse occurs.

There is data from case series to suggest that Eculizumab withdrawal with rapid re-initiation of treatment in the case of relapse is a safe and effective treatment strategy. This study will formally assess the safety and cost-effectiveness of Eculizumab withdrawal. The protocol has been designed to detect disease relapse

early and therefore facilitate rapid re-initiation of treatment within 24 hours of presentation.

This trial is categorised as:

- Type B = somewhat higher than the risk of standard clinical care

# 3. OBJECTIVES AND OUTCOME MEASURES

## 3.1. Primary Objective

The primary clinical objective is to determine the safety of Eculizumab withdrawal in patients with aHUS.

## 3.2. Secondary Objectives

*1*. Measure the effectiveness of a monitoring protocol to detect disease relapse following withdrawal of Eculizumab.
*2.* Describe the relapse rate after withdrawal of Eculizumab.
*3*. Estimate the proportion of patients, currently on long-term treatment with Eculizumab, who can be maintained off treatment.
*4.* Describe the period from withdrawal to relapse in those patients who restart treatment.
*5.* Measure the change in estimated GFR (calculated by the CKD-EPI or modified Schwartz equations) over the course of the study.
*6.* Identify important clinical and laboratory indicators of imminent relapse.

*Heath Economic Objectives*

*7.* To assess the costs and health outcomes (measured in terms of adverse events and quality-adjusted life years (QALYs)) for patients on standard care (not withdrawing from Eculizumab treatment) over the two-year trial duration.
*8.* To assess the costs and health outcomes for patients fully, or partially, withdrawing from Eculizumab treatment, and on a policy of protocolised monitoring, over the two-year trial duration.
*9.* To model the costs and health consequences (measured in terms of QALYs) associated with Eculizumab withdrawal, and a policy of protocolised monitoring following withdrawal (and treatment re-introduction if necessary), compared with standard care, beyond the two-year timeframe of the trial.

## 3.3. Primary Outcome Measures

Number of patients with a TMA related Serious Adverse Event (SAE) defined as any of the following:

- Irreversible (>3 months) reduction in estimated glomerular filtration rate (eGFR) not attributable to another cause:

**In adults**

- by ≥20% if the screening eGFR is <90mls/min/1.73m$^2$
- by >20% to a level <90mls/min/1.73m$^2$ if the screening eGFR is >90mls/min/1.73m$^2$

**In children**

- by ≥20% if the screening eGFR is <75mls/min/1.73m$^2$
- by >20% to a level <75mls/min/1.73m$^2$ if the screening eGFR is >75mls/min/1.73m$^2$

The equations used to obtain eGFR are known to be inaccurate above a value of 90 mls/min/1.73m$^2$ for adults and 75 mls/min/1.73m$^2$ for children. It is plausible that a ≥20% reduction in eGFR could be due to this inaccuracy or other outside clinical factors. Where an irreversible reduction in eGFR of ≥20% is observed, the case will be reviewed by the DMC assess the causal relationship to eculizumab withdrawal

- An episode of AKI attributed to a TMA that requires renal replacement therapy.
- A non-renal manifestation of a TMA that require hospitalisation, cause irreversible organ damage or death.

## 3.4. Secondary Outcome Measures

1. The effectiveness of a monitoring protocol to detect disease relapse following withdrawal of Eculizumab assessed by:
i. The proportion of patients who relapse and restart Eculizumab without the development of a TMA-related SAE (section 3.3).
ii. The time from the first clinical feature (symptom, positive urinalysis or laboratory result) of a relapse of TMA and the re-introduction of Eculizumab treatment.
2. The relapse rate after withdrawal of Eculizumab as determined by the proportion of patients who relapse after Eculizumab is withdrawn.
3. Estimate of the proportion of patients, currently on long-term treatment with Eculizumab, who can be maintained off treatment.
4. Description of the period from withdrawal to relapse in those patients who restart treatment measured from baseline (day 0) to day of re-introduction of treatment or end of the study.
5. The change in estimated GFR as calculated by the CKD-EPI or modified Schwartz equations over the course of the study from baseline (day 0) to end of the study.
6. Identification of important clinical and laboratory indicators of imminent relapse by review of reported symptoms, physical signs, urinalysis and laboratory results prior to the diagnosis of a relapse.

*Heath Economic Outcome Measures*

7. Cumulative costs to the NHS and participants, and determinants of costs, over the 24-month follow-up period.

8. QALYs estimated from responses to the EQ-5D-5L, and SF-36, completed at pre-determined time points, and determinants of QALYs/utilities, over the 24-month follow-up period.

9. Model-based estimate of the costs and health consequences, with results presented in terms of cost per QALY gained, over the estimated lifetime of patients withdrawing from treatment compared with standard care.

# 4. TRIAL DESIGN

Single arm, open label study with continuous monitoring of serious adverse events using the Bayes factor single arm design of Johnson and Cook (4). The small number of patients on treatment are insufficient to conduct a standard parallel group randomised, controlled trial. An economic analysis, informed by the results of this trial, will determine whether Eculizumab withdrawal, substituting treatment with a protocolised surveillance and treatment reintroduction strategy, is cost-effective.

# 5. STUDY SETTING

20 Renal units (secondary care) in the UK who are using Eculizumab to treat patients with aHUS. All NHS organisations providing care for patients with aHUS on Eculizumab treatment will be eligible to participate in this study. We will invite one physician responsible for these patients to act as principal investigator for the study at each site. A significant element of the study relates to safety assessments and will therefore involve clinical staff at the site.

Research staff may also be involved in the visits, however, as many of the visits relate to safety the involvement of research nurses will not be an absolute requirement. This will allow sites with limited research infrastructure to participate in the study, however some research administration will be required. If this is not available in sites that want to participate, the CTU will explore options with local Research Networks.

An additional consideration for participating sites is that the number of study visits for patients who withdraw from treatment is less than the visits required if treatment is continued. We have approached physicians at suitable sites and identified potential principal investigators who have expressed an interest in participating.

# 6. ELIGIBILITY CRITERIA

Patients with a diagnosis of aHUS (based on defined criteria – www.rarerenal.org) receiving Eculizumab to treat disease in native or transplanted kidneys. This is estimated to be 150 patients in the UK during the study period (110 prevalent patients and 40 incident patients). Genetic eligibility will be confirmed by the central trial team who will contact sites to confirm that they have a potentially eligible patient for site screening. Site eligibility must be assessed by a medically qualified doctor, and for those patients who enter into the trial, this assessment must be documented in the participant's medical notes. A copy of both the anonymised genetic eligibility checklist AND the site eligibility checklist must be filed in the participant's medical notes and sent to the NCTU as instructed on the forms. The eligibility checklist must also be completed in the MACRO database for those patients who enter into the trial. Only personnel formally delegated by the Principal Investigator to assess eligibility may perform this task.

## 6.1. Inclusion Criteria

All patients must fulfil the following criteria in order to be eligible for the trial:

- Age ≥2+ years of age
- On Eculizumab treatment for at least 6 months

- In remission with no evidence of ongoing microangiopathic haemolytic anaemia (MAHA) activity at screening defined by:
  - Platelet count > lower limit of normal as determined by local reference range
  - Lactate Dehydrogenase (LDH) <x2 upper limit of normal as determined by local lab reference ranges
- Normal renal function or Chronic Kidney Disease (CKD) stages 1-3
- Absence of decline of renal function confirmed by review of available assessments of renal function for the preceding 6 months by the Chief Investigator and clinical members of the Trial Management Group (TMG)

The following criteria must be met by those only wishing to participate in the withdrawal component of the trial:

- Willing to attend for safety monitoring assessments

- Willing to travel only to countries that can supply Eculizumab (to be confirmed with co-ordinating centre prior to travel).

- Able to perform or parent/guardian to perform and record self-monitoring urinalysis

- Sexually active female patients must have a negative pregnancy test at screening and be using an effective contraception for the duration of the study as defined in table 1. If not sexually active at the time of consent, the patient must be advised that, if at any point during the trial they do become sexually active, they must use an effective contraception method as defined in table 1.

OR

- fulfil one of the following criteria:
  - Be post-menopausal
  - Have undergone surgical sterilisation

| **Highly effective methods of contraception** | **Failure Rate** |
|---|---|
| Implant | <1% |
| Injectable (combined hormone) | <1% |
| Intra-uterine device | <1% |
| Intrauterine system | <1% |
| Male Sterilisation | <1% |
| Injectable (single hormone) | <1% |
| Combined Oral | <1% |
| Progesterone Only | 1% |

**Table 1. Recommended Birth Control Methods**. (http://www.chcuk.co.uk/pdf/2011-03-12_GCP_Considerations_Contraception_(CHCUK).pdf)

## 6.2. Exclusion Criteria

The following exclusion criteria is applicable to all patients wishing to participate in the trial:

- Severe non-renal disease manifestations at initial presentation with aHUS, which in the opinion of the Chief Investigator and/or the clinical members of the TMG makes the risk of treatment withdrawal unacceptable.

- Current or planned pregnancy within the study duration

- Unable to give informed consent or assent, or unable to obtain parent/guardian consent if under 16 years of age

- Current participation in another clinical trial (not including participation in ahus registries)

- Severe, uncontrolled hypertension (systolic blood pressure >160 mmHg) that is likely to induce at TMA.

The following exclusion criteria is applicable only to those wishing to participate in the withdrawal component of the trial:

- Loss of a previous transplant kidney to recurrent aHUS
- Transplant recipient with a pathogenic mutation in *C3*, *CFH* or *CFB*
- Haematuria rating of 3+

**NB: Enrolling a patient onto the trial who does not meet the inclusion/exclusion criteria is considered a protocol waiver and is in breach of Regulation 29 (SI 2004/1031) of the Medicines for Human Use (Clinical Trials) Regulations 2004. PROTOCOL WAIVERS ARE NOT PERMITTED.**

# 7. TRIAL PROCEDURES

## 7.1. Recruitment

### 7.1.1 Patient Identification and Central Pre-Screening

Patients with a diagnosis of aHUS (based on defined criteria – www.rarerenal.org) receiving Eculizumab to treat disease in native or transplanted kidneys will be identified by the central clinical team at Newcastle as potentially eligible for the trial. A list of patients who fulfil these criteria is maintained by the National aHUS Service based within the NRCTC as part of the NHS England commissioned service. This information is available to the central clinical trial team and will allow identification of patients who potentially fulfil the eligibility criteria. The central clinical trial team will assess eligibility by review of the patient's medical history available on the National aHUS service database. Those patients who meet the genetic eligibility criteria will be highlighted to sites.

It is estimated that there will be 150 patients in the UK who are on Eculizumab for the treatment of aHUS during the study period (110 prevalent patients and 40 incident patients). From our preliminary assessment of the current patient cohort

(approximately 100 patients) 30% will not meet the inclusion/exclusion criteria, leaving a pool of approximately 60-70 prevalent patients eligible to participate in the study.

**7.1.2. Site Screening (day -28 to -14)**

On receipt of the Central pre-screen eligibility checklist, the site can begin screening activities. This will include a review of the patients' notes for their medical history and concomitant medications by the site research team. A physical examination and vital signs will be performed and routine safety laboratory tests will be reviewed to ensure that the patient fulfils all eligibility criteria (section 6.1 & 6.2) for entry into the study (please see section 7.4-Schedule of events, day -28 to day -14). Site screening activities may occur up to 4 weeks prior to Baseline (Day 0 +/- 2 days) and may occur on day -14 when the patient receives their final Eculizumab infusion. Any queries regarding patient eligibility should be considered first by the PI at site and further discussed with the CI or member of the central clinical trial team before the patient is formally consented.

Recruitment of patients who are non-English speakers will be reviewed on a case by case basis. If the site has access to an interpreter who can attend all monitoring visits, the central research team will explore the possibility of arranging to have the patient documentation translated into the required language.

Once patients have consented to take part in the trial, those female participants who are taking part in the withdrawal component and are sexually active will be required to have a pregnancy test as part of the screening process.

**7.1.3. Consent**

Informed consent and assent will be sought by a member of the trial team who is suitably qualified, appropriately trained and delegated to do so. Potential participants, or their parents/legal guardian for a minor, will be given at least 24 hours after being given the Patient Information Sheet (PIS) to consider their participation in the study. As part of the consent and/or assent discussion, the requirement for sexually active females to use effective contraception must be discussed and confirmation of this discussion documented as a statement in the patients notes. Consent will be sought from the parents/legal guardian on behalf of patients under the age of 16. Assent will be taken from those patients under 16 years old.

The original signed Consent and assent forms will be retained in the Investigator Site File (ISF), with copies provided to the patient, a copy filed in the patients' clinical notes and a copy sent by secure methods to NCTU. The copy sent to NCTU for monitoring purposes will be destroyed once reviewed. The site will allocate the participant their Unique Identification Number once they have consented to the study. Please note that this is different to the screening number that will be given to sites by the central trial team following genetic eligibility confirmation. The Unique ID number for withdrawal patients will be made up of the 2 digit site code and a 3 digit patient code starting with 001 for the first patient and continuing

sequentially with each consenting patient. Those participants who are taking part in the Health Economics component only will have a unique ID number made up of the 2 digit site code and a 3 digit patient code starting with 101 for the first patient and continuing sequentially with each consenting patient.

**7.1.4. Final Eculizumab Infusion (Visit 1, Day -14)**
Patients who consent to withdrawal will receive their last dose of Eculizumab at this visit (day -14). The patients will also receive their meningococcal prophylaxis as usual.

## 7.2 Trial Assessments

### 7.2.1. Baseline Assessments & Data (Visit 2, Day 0 +/- 2 days)

Study day 0 will be the day that the patients would usually receive their next dose of Eculizumab based on standard dosing schedules (+/-2 days). The Eculizumab will not be administered however, the meningococcal prophylaxis will be continued for a further 2 weeks after day 0 to reduce risk of meningococcal infection. Patients will be trained at site to complete home urinalysis. This training must be documented in the patients notes.

At day 0 of the study (Visit 2), Eculizumab will not be administered. Patients will undergo the following assessments:

- Vital signs (temperature, pulse and Blood pressure)
- Height & weight
- Assessment of renal function (creatinine and estimated GFR).
- Urinalysis and urine protein/creatinine ratio
- Haemolysis markers including platelet count, haemoglobin, LDH
- Electrolyte Profile (U&Es)
- Liver Function (Bilirubin, ALT/AST, ALP, LDH, serum calcium, phosphate, albumin & total protein)
- Haptoglobin (if available) and blood film
- Concomitant Medication review
- Health-related quality of life questionnaires (EQ-5D-5L and SF-36)
- Health care utilisation questionnaire
- Biomarkers and complement activation sample – Sample to be taken and stored at site before transfer to Newcastle University for analysis (see 7.6).

### 7.2.2. Study Visits (Visits 3-34)

#### 7.2.2.1. Clinical Assessments

Patients will be assessed regularly for evidence of disease relapse for the 2 year duration of the study. The patients will attend a total of 34 visits* over the 2 year withdrawal follow-up period. Please see schedule of events (Section 7.4).

Study participants will be reviewed at the study site weekly (+/- 2 days) for the first month then alternate weeks (+/-2 days) until month 6, then monthly (+/- 7 days) thereafter until the end of the study period (month 24). During

the COVID-19 pandemic and due to site staff availability, it is understood that patients may be seen outside of but as close to the visit window as possible. At each study visit, patients will undergo the following assessments:

- Vital signs (temperature, pulse and Blood pressure)
- Renal function (creatinine and estimated GFR)
- Urinalysis
- Haemolysis markers including platelet count, haemoglobin and LDH
- Electrolyte Profile (U&Es)
- Liver function (Bilirubin, ALT/AST, ALP, LDH, albumin, total protein, serum calcium & phosphate)
- Concomitant Medication review
- Urinalysis Diary review
- Adverse Events

*Due to the covid-19 pandemic, patients may be unable to attend their scheduled follow up visits or may be attending a local hospital to have safety bloods taken. If the patients are unable to attend site due to self-isolation or underlying health issues; where possible, a remote, follow-up call will be carried out by a member of the local research team. During the remote follow-up call patients will be asked to report changes to their concomitant medications, any adverse events experienced since their previous follow-up and the results of their home urinalysis tests that will continue to be recorded in their urinalysis diary.

Paediatric participants must have their weight recorded at every visit for calculation of estimated GFR.

As haptoglobin is not a standard test that is performed at every NHS hospital, this is not a compulsory measure for those sites where it is not performed. Haptoglobin availability should be discussed with the central research team prior to starting recruitment at site.

Participants will provide the following at visits 2, 3, 4, 6, 8, 10, 16, 19, 22, 28 and 34.

- Biomarkers and complement activation samples

Participants will undergo the following assessments at study visits 2, 6, 10, 16, 22, 28 and 34.

- Haptoglobin (if available) and blood film
- Urine PCR

Participants will undergo the following assessments at study visits: 22 & 34:

- Physical Examination*

*Due to the COVID-19 pandemic and social distancing requirements and guidelines set out by individual hospital Trusts, patients may not be able to meet face to face with a clinician for their physical examination at the timepoints stated in the schedule of events table (section 7.4).

Following each patients visit the test results and assessment data collected should be entered into the MACO database in a timely manner and before the patients next scheduled visit.

**7.2.2.2. Health Economic Assessments**

Patients, or legal guardians/carers if patients is under 16 years who are taking part in the withdrawal trial, and those who are completing the Health Economics component only will complete the following assessments at visits 2, 4, 9, 15, 19, 22, 28 and 34:

- EQ-5D-5L (proxy version if patient < 12 years)
- SF36 (parent/guardian completes if patient <14 years)
- Health care utilisation questionnaire

The following assessment will be completed at visit 28 only:

- Time and Travel questionnaire

The Health Economic Questionnaires can be completed by patients at home or in the clinic during their study visit or standard clinic appointment. For those patients completing the questionnaires at home, these can be sent to the patient either by a site team member or, if required, by a member of the central team who is part of the National aHUS service and has permission to access the patients contact details. The questionnaires will be sent back to NCTU in a pre-paid envelope, to be entered into MACRO by the central trial team.

Health Economic analysis is required to commence by the 01 November 2023, any outstanding questionnaires due after this time point will not be completed by participants.

**7.2.3. Self-monitored Urinalysis**

Patients or carers will be trained to perform and understand the results of home urinalysis. Urinalysis will then be performed daily by the patient or carer for the first month and then three times per week for the remainder of the study period. The results will be recorded in a patient-held record and will be reviewed at each study visit (see Section 7.4. Schedule of Events). The haematuria ratings documented in the patient diary will be reviewed by the research nurse at each safety monitoring visit and any significant changes from baseline will be entered into MACRO at site. A new 2 week diary will be given to patients at each visit and the completed diaries will be retained by the site for monitoring purposes. During the COVID-19 pandemic, patients who are unable to attend site, those who are attending a local hospital for safety bloods or having bloods taken at home will be contacted where possible by a member of the local research team who will review their urinalysis diary over the telephone.

Patients or carers will report any significant change in urinalysis that is not related to menstruation to the study site using their own baseline to guide them in relation to the thresholds as detailed in table 2 below:

| Baseline | urinalysis result threshold<br>(not related to menstruation) |
|---|---|
| Neg/Trace | ++ on any occasion<br>OR<br>+ on any two occasions 24 hours apart |
| + | +++ on any occasion<br>OR<br>++ on any two occasions 24 hours apart |
| ++ | +++ on any occasion |

Table 2. Home Urinalysis result thresholds

If these criteria are met, patients will contact their treatment centre immediately to arrange assessment of disease activity (Renal function, haemoglobin, platelets, LDH, LFTs, haptoglobin and blood film, complement status) as outlined in the Schedule of Events Unscheduled visit column (See section 7.4).

#### 7.2.4. Change in Health Status

Patients participating in the study will also be advised to report any significant change in health status to the responsible site or local health care provider. Patients will be provided with a patient card to present to attending medical staff with details of the study, tests required and study centre and National aHUS Service contact details. Sites will send out the GP letter provided to notify the patients' General Practitioner of their involvement in the study and informing them of the required action to be taken in the case of suspected relapse.

If there is a clinical suspicion of disease activity formal assessment will occur (renal function, haemoglobin, platelets, LDH, LFTs, haptoglobin, blood film, U&Es and complement status). At each study visit, patients will be asked about previously unreported adverse or serious adverse events that may have occurred since the last contact.

During the COVID-19 pandemic, patients who are unable to attend site, those who are attending a local hospital for safety bloods or having bloods taken at home will be contacted remotely where possible by a member of the local research team who will ask about previously unreported adverse or serious adverse events that may have occurred since the last contact.

## 7.3 Disease Relapse

### 7.3.1 Definition of disease relapse

A relapse in aHUS after withdrawal of Eculizumab will be diagnosed by:

1. Haematological relapse with a MAHA evidenced by the presence of the following criteria:

   a. Platelet count of <150 x 109 /l (if normal at screening) or a fall in platelet count by >50% from screening

   b. An increase in LDH by >50% above screening or haemoglobin < lower limit of normal for age and gender

OR

2. The presence of renal disease manifest by a decline in renal function (Acute Kidney Injury Network stage 1 AKI) confirmed on repeat testing after 6 hours) that is not explained by another pathology

OR

3. The presence of histological features of an active TMA on tissue biopsy performed as per local protocols for the investigation of renal disease.

Any other adverse events that could represent a relapse should be discussed with the Investigators and/or the aHUS National Service. There is a Consultant

physician on call for the aHUS National Service at all times who should be informed by the responsible physician of any case where there is diagnostic uncertainty. A decision to restart will be made according to current Service procedures, requiring a consensus opinion from the consultant staff following review of the available clinical information.

### 7.3.2 Management of a Relapse

#### 7.3.2.1. Reporting a relapse

Any patient with a suspected relapse should be reported to the PI at the local site, the NCTU and the aHUS National Service. This event must be reported in the MACRO database on the *possible relapse form* in the unscheduled visit section. If the suspected relapse meets the SAE criteria listed in section 10.1, it should also be reported as such, following the procedure outlined in section 10.2 of this document.

#### 7.3.2.2. Restarting treatment following relapse

When a relapse is diagnosed patients will restart Eculizumab treatment within 24 hours of presentation:

- Provided there is no evidence of an active infection that would be a contra-indication to treatment;
- At the recommended dose of 900mg weekly for the first 4 weeks then 1200mg every two weeks thereafter (or age adjusted dose);
- With monitoring of TMA activity (platelet count, LDH) as recommended by attending clinician until haematological remission is achieved. Haematological remission is defined as both:
  - Normalisation of platelet count (>150 x $10^9$ /l)
  - Normalisation of serum LDH or within 50% of baseline level
- Maintenance of remission and effect of treatment will be monitored according to current recommended guidelines (www.rarerenal.org). This consists of:
  - Laboratory tests to confirm complement blockade (CH50 and AP50) prior to 2nd and 6th dose of Eculizumab
  - Tests to confirm TMA remission (haemoglobin, platelet count, LDH, haptoglobin, urinalysis and urine protein creatinine ratio) monthly until the end of the study.

Patients who relapse and require re-introduction of Eculizumab treatment will remain on treatment in study under follow up for the full 2 years of the study. Hospital attendance for administration of treatment will constitute a study visit with reporting of adverse events. The frequency of safety bloods following relapse and treatment reintroduction should align with local standard of care for patients receiving Eculizumab. The dates that haematology and biochemistry results are obtained may not align with the participants planned trial visit schedule however, the results should be entered into the MACRO database for the corresponding months. If this is outside of the visit window for that particular month, this will not be

considered a deviation. Home urinalysis will not be required after re-introduction of Eculizumab treatment.

Complement samples **should** be taken when a participant relapses in the UK. If the site does not have storage facilities, transportation to Newcastle will be arranged at no additional cost to the site. In such cases the site must contact the aHUS National Service on 0191 2820385 (9am to 5pm) or the Newcastle upon Tyne Hospitals switchboard (0191 2336161) requesting to speak to the clinician on call for the aHUS service to arrange this.
Due to the COVID-19 pandemic it is understood that it may not be possible for the biomarker and complement activation samples to be obtained and processed due to research staff availability. The Newcastle biobank will be closed due to government lockdown guidelines and will not be able to accept any research samples during this time. Sites will be informed when the biobank will be open to receive samples.

Patients will consent to travel to only those countries where Eculizumab is available, and this must be confirmed with their clinician prior to booking any travel. The site should inform the National aHUS service if a patient plans to travel outside of the country. The destination and dates of travel should be disclosed so that a potential relapse event can be dealt with as rapidly as possible. Patients should be reminded that they will need to continue to complete the urinalysis diary while on holiday as this is an indicator of disease activity. Patients must also be reminded to carry their Patient card at all times and to present this card to the clinician treating them should they visit a hospital while outside of the country. If patients relapse while they are travelling outside of the country, the National aHUS Service will make arrangements with the destination country to access and fund Eculizumab if required, with arrangements from the commissioning authority, NHS England. Sponsor insurance does not cover a patient’s hospital treatment and other associated costs while travelling outside of the country and participants are reminded in the Patient Information Sheet that they must arrange their own travel insurance to cover these costs and inform their insurance provider of their participation in the Clinical Trial.

## 7.4 Schedule of Events for withdrawal participants

| | Central Pre-Screen | Site Screen and Consent | Final Infusion | Withdrawal Phase | | | | | | | | | | | | | | | | | | | | | | | | | | | | | | | | |
|---|---|---|---|---|---|---|---|---|---|---|---|---|---|---|---|---|---|---|---|---|---|---|---|---|---|---|---|---|---|---|---|---|---|---|---|---|
| **Month** | 0 | 0 | 0 | 1 | | | | | 2 | | 3 | | 4 | | 5 | | 6 | | 7 | 8 | 9 | 10 | 11 | 12 | 13 | 14 | 15 | 16 | 17 | 18 | 19 | 20 | 21 | 22 | 23 | 24 | Unscheduled Visit |
| **Visit Number** | N/A | N/A | 1 | 2 | 3 | 4 | 5 | 6 | 7 | 8 | 9 | 10 | 11 | 12 | 13 | 14 | 15 | 16 | 17 | 18 | 19 | 20 | 21 | 22 | 23 | 24 | 25 | 26 | 27 | 28 | 29 | 30 | 31 | 32 | 33 | 34* | |
| **Day** | -42 | Day -28 to (and inc) Day -14 | -14 | 0 | 7 | 14 | 21 | 28 | 42 | 56 | 70 | 84 | 98 | 112 | 126 | 140 | 154 | 168 | 196 | 224 | 252 | 280 | 308 | 336 | 364 | 392 | 420 | 448 | 476 | 504 | 532 | 560 | 588 | 616 | 644 | 672 | |
| Genetic Eligibility | x | | | | | | | | | | | | | | | | | | | | | | | | | | | | | | | | | | | | |
| Medical History Review | | x | | | | | | | | | | | | | | | | | | | | | | | | | | | | | | | | | | | |
| Informed consent | | x | | | | | | | | | | | | | | | | | | | | | | | | | | | | | | | | | | | |
| Eligibility Checklist Completion | | x | | | | | | | | | | | | | | | | | | | | | | | | | | | | | | | | | | | |
| Physical Examination | | x | | | | | | | | | | | | | | | | | | | | | | x | | | | | | | | | | | | x | x |
| Height & Weight | | x | | | | | | | | | | | | | | | | | | | | | | | | | | | | | | | | | | | |
| Pregnancy test | | x | | | | | | | | | | | | | | | | | | | | | | | | | | | | | | | | | | | |
| Eculizumab Infusion | | | x | | | | | | | | | | | | | | | | | | | | | | | | | | | | | | | | | | |
| Meningococcal prophylaxis | | | x | x | | | | | | | | | | | | | | | | | | | | | | | | | | | | | | | | | |
| Urine analysis training | | | | x | | | | | | | | | | | | | | | | | | | | | | | | | | | | | | | | | |
| Vital Signs | | x | | x | x | x | x | x | x | x | x | x | x | x | x | x | x | x | x | x | x | x | x | x | x | x | x | x | x | x | x | x | x | x | x | x | x |
| Concomitant medication Review | | x | | x | x | x | x | x | x | x | x | x | x | x | x | x | x | x | x | x | x | x | x | x | x | x | x | x | x | x | x | x | x | x | x | x | x |
| Renal Function (Creatinine & GFR) | | x | | x | x | x | x | x | x | x | x | x | x | x | x | x | x | x | x | x | x | x | x | x | x | x | x | x | x | x | x | x | x | x | x | x | x |

| | Central Pre-Screen | Site Screen and Consent | Final Infusion | Withdrawal Phase | | | | | | | | | | | | | | | | | | | | | | | | | | | | | | | | | |
|---|---|---|---|---|---|---|---|---|---|---|---|---|---|---|---|---|---|---|---|---|---|---|---|---|---|---|---|---|---|---|---|---|---|---|---|---|---|
| **Month** | 0 | 0 | 0 | 1 | | | | | 2 | | 3 | | 4 | | 5 | | 6 | | 7 | 8 | 9 | 10 | 11 | 12 | 13 | 14 | 15 | 16 | 17 | 18 | 19 | 20 | 21 | 22 | 23 | 24 | Unscheduled Visit |
| **Visit Number** | N/A | N/A | 1 | 2 | 3 | 4 | 5 | 6 | 7 | 8 | 9 | 10 | 11 | 12 | 13 | 14 | 15 | 16 | 17 | 18 | 19 | 20 | 21 | 22 | 23 | 24 | 25 | 26 | 27 | 28 | 29 | 30 | 31 | 32 | 33 | 34* | |
| **Day** | -42 | Day -28 to (and inc) Day -14 | -14 | 0 | 7 | 14 | 21 | 28 | 42 | 56 | 70 | 84 | 98 | 112 | 126 | 140 | 154 | 168 | 196 | 224 | 252 | 280 | 308 | 336 | 364 | 392 | 420 | 448 | 476 | 504 | 532 | 560 | 588 | 616 | 644 | 672 | |
| Liver Function Tests | | x | | x | x | x | x | x | x | x | x | x | x | x | x | x | x | x | x | x | x | x | x | x | x | x | x | x | x | x | x | x | x | x | x | x | x |
| Haemolysis markers (full blood count & LDH) | | x | | x | x | x | x | x | x | x | x | x | x | x | x | x | x | x | x | x | x | x | x | x | x | x | x | x | x | x | x | x | x | x | x | x | x |
| Electrolyte profile (U&Es) | | x | | x | x | x | x | x | x | x | x | x | x | x | x | x | x | x | x | x | x | x | x | x | x | x | x | x | x | x | x | x | x | x | x | x | x |
| Haptoglobin & Blood film | | x | | x | | | | x | | | | x | | | | | | x | | | | | | x | | | | | | x | | | | | | x | x |
| Urine PCR | | x | | x | | | | x | | | | x | | | | | | x | | | | | | x | | | | | | x | | | | | | x | x |
| Biomarkers and complement activation sample | | x | | x | x | x | | x | | x | | x | | | | | | x | | | x | | | x | | | | | | x | | | | | | x | x |
| Home Urinalysis | | x | | Daily | | | | 3 times per week | | | | | | | | | | | | | | | | | | | | | | | | | | | | | |
| Home Urinalysis | | | | | x | x | x | x | x | x | x | x | x | x | x | x | x | x | x | x | x | x | x | x | x | x | x | x | x | x | x | x | x | x | x | x | |
| Adverse Events | | | | | x | x | x | x | x | x | x | x | x | x | x | x | x | x | x | x | x | x | x | x | x | x | x | x | x | x | x | x | x | x | x | x | x |
| EQ5D & SF36 | | | | x | | x | | | | | x | | | | | | x | | | | x | | | x | | | | | | x | | | | | | x | |
| Health care | | | | x | | x | | | | | x | | | | | | x | | | | x | | | x | | | | | | x | | | | | | x | |
| Time & Travel Questionnaire | | | | | | | | | | | | | | | | | | | | | | | | | | | | | | x | | | | | | | |

Table 2. Schedule of events for withdrawal participants, day -42 to 672

*Health Economic analysis is required to commence by the 01 November 2023, therefore, if Visit 34 questionnaires are not completed by this date they will not be required to be completed by the participant. All other visit 34 items must be completed.

### 7.5 Schedule of Events for Health Economics participants

| | Central Screen | Folllow - up | | | | | | | |
|---|---|---|---|---|---|---|---|---|---|
| month | 0 | 1 | | 3 | 6 | 9 | 12 | 18 | 24 |
| visit number | N/A | 1 | 2 | 3 | 4 | 5 | 6 | 7 | 8 |
| day | -42 | 0 | 14 | 70 | 154 | 252 | 336 | 504 | 672 |
| | | | | | | | | | |
| Medical History Review | | x | | | | | | | |
| Informed consent | | x | | | | | | | |
| Eligibility Checklist | | x | | | | | | | |
| EQ5D & SF36 | | x | x | x | x | x | x | x | x |
| Health care Utilisation questionnaire | | x | x | x | x | x | x | x | x |
| Time & Travel Questionnaire | | | | | | | | x | |

Table 3. Schedule of events for Health Economics only participants, day -42 to 672

## 7.6 Trial Withdrawal Criteria

Participants have the right to withdraw from the trial at any time without having to give a reason. Investigators at sites should try to ascertain the reason for withdrawal and document this reason within the eCRF and participant's medical notes.

The Investigator may discontinue a participant from the trial at any time if the Investigator considers it necessary for any reason including:

- Participant withdrawal of consent
- Significant protocol deviation or non-compliance
- Investigator's discretion that it is in the best interest of the participant to withdraw from the study not relating to re-starting Eculizumab treatment
- An adverse event that renders the participant unable to continue in the trial
- Termination of the clinical trial by the sponsor

Participants who withdraw from the trial will not be replaced.

## 7.7 Storage and Analysis of Samples

Standard laboratory tests and haemolytic activity will be performed locally as per site protocols.

Biomarkers and Complement activation samples will be transported for central analysis in Newcastle. These are collected at visits 1, 2, 3, 4, 6, 8, 10, 16, 19, 22, 28 and 34.
These samples should be stored in a -80°C freezer until transfer to Newcastle University tissue Bank. Please see the separate laboratory guide for further instructions.

Sites that do not have adequate storage facilities (-80 freezer) and so are unable to take the complement samples from patients on study visits will not be precluded from taking part in the study. However, should a patient relapse at a site that does not have adequate storage facilities, the sample **MUST** be taken and immediate transportation to Newcastle will be arranged by contacting the aHUS National Service.

On arrival in Newcastle samples will be stored in a -80°C freezer in the Newcastle University Tissue Biobank until they are batched for exploratory biomarker analysis. Once the samples have been analysed for the purpose of this study, they will be banked for use in future renal research if the patient has consented to this.

It is the responsibility of the trial site to ensure that samples are appropriately labelled in accordance with the trial procedures to comply with the Data Protection Act. Biological samples collected from participants as part of this trial will be transported, stored, accessed and processed in accordance with national legislation relating to the use and storage of human tissue for research purposes and such activities shall at least meet the requirements as set out in the 2004 Human Tissue Act and 2006 Human Tissue (Scotland) Act.

## 7.8 End of Trial

The last visit will be defined as the last patient attending their visit 34 on day 672 (+/-7 days).

The final treatment outcome data will be collected when the final patient attends for their visit 34. At this point the end of study notification will be submitted to the Research Ethics Committee (REC). The data analysis and final report will be completed within a year of this study notification.

The trial may be ended prematurely on the recommendation of the DMC & TSC if SAE's or interim analysis indicates that this is required. The criteria of such decisions will be decided by the DMC & TSC as specified in the respective charter and SAP.

## 7.9 COVID-19 Pandemic

Due to the COVID-19 pandemic it is possible sites may need to make a number of changes to the planned follow up visits to ensure that patients remain safe in the trial while off drug. It is possible site research teams may need to organise local hospital safety bloods or explore safety bloods to be taken at home by community research nurses for those patients who are unable to attend site for reasons associated with COVID-19. In the case where bloods are taken locally sites will need to ensure that results from the local blood tests are made available to them for review and input into the trial database for central review in a timely manner. This will facilitate the essential rapid identification of any disease activity that may occur during this time so that patients can have their Eculizumab treatment restarted immediately and within 24 hours of the site becoming aware of disease activity.

Due to the COVID- 19 social distancing guidelines set out by individual hospital Trusts it may not be possible for patients, who continue to attend site for safety bloods, to have face to face contact with the research team. All patients who are unable to have their follow up assessments carried out face to face will be, where possible, contacted remotely via telephone by site research team to assess adverse events, changes in concomitant medications and home urinalysis results as recorded in the patient diaries since the previous follow up visit.

All patients have been reminded that they must continue to carry out their home urinalysis tests and to continue to record their results in the patient diaries. They have also been reminded of the telephone numbers of the site team and the National aHUS Service who they should contact if they begin to feel unwell in any way as well as the aHUS national service website where they can access up to date information and support (http://www.atypicalhus.co.uk/). Some of these details are also available on their patient card.

The NCTU management team have compiled an individual patient risk assessment document that is updated on an ongoing basis. This document states where patients are having their safety bloods taken if this is possible and any other risks that may be significant to individual patients and how these risks can

be mitigated. The risk assessment document is reviewed on a monthly basis by members of the TMG and Sponsor.

# 8. HEALTH ECONOMICS

The economic analysis will comprise: a) a within-trial assessment of the costs and outcomes associated with successful withdrawal from Eculizumab, and the costs and outcomes associated with standard care, over a two-year time horizon, in order to provide information for the definitive model-based economic evaluation, and b) an economic model to assess the costs and health consequences, measured in terms of QALYs, of Eculizumab withdrawal, and a policy of monitoring following withdrawal, at two years and beyond the timeframe of the trial. The results of the model-based economic evaluation will be presented in terms of incremental cost per QALY gained. The economic analyses will take the perspective of the UK NHS and Personal Social Services (PSS), as well as aHUS patients and their families (e.g. time lost from usual activities, travel time and monetary costs of accessing care). Health Economic analysis is required to commence by the 01 November 2023, any outstanding questionnaires due after this time point will not be completed by participants.

## 8.1. Health economic data collection

### 8.1.1. Assessment of cost

Information on costs will be collected for all trial participants, including the additional 20 patients who are not involved in the withdrawal component of the study, but who agree to remain involved in the health economic and qualitative components (30 patients who withdraw + 20 patients who do not withdraw). Use of primary care services and private/out-of-pocket purchases of health care, and time away from usual activities, will be collected using participant-completed health utilisation questionnaires. These questionnaires will be administered at baseline, and at 1, 3, 6, 9, 12, 18 and 24 months post-consent.

Information on resource use of secondary health care services will be collected via case report forms (CRFs). Adverse events resulting in hospitalisation and/or resulting in care and associated healthcare costs (based on time in care, grade of health care staff, equipment and consumables used) will be collected using CRFs completed at baseline, and at 1, 3, 6, 9, 12, 18 and 24 months post-consent. Information on use of services will be combined with information on unit costs of those services obtained from standard sources PSSRU, NHS Reference costs (20).

Estimates of costs incurred accessing health care services, for both patients and their families, will also be captured. A time and travel questionnaire, based upon one successfully used in a number of NIHR- funded HTA trials, will be administered at 18 months post-consent. In this questionnaire, trial participants will be asked about out-of-pocket expenses relating to the attendance of appointments. These costs will be allocated to every journey a participant and/or their main caregiver makes for health care treatment, and multiplied by the number of appointments recorded over the 24-month trial duration (collected via the health service utilisation questionnaire).

Participants will also be asked about the main activities they would otherwise be undertaking if they did not have to attend appointments, and the main activities their carers, if relevant, would have been undertaking if they were not attending the appointment with them. Thus, the patient and caregiver costs can be separated into direct costs (e.g. out-of-pocket payments) and indirect costs (e.g. the time costs of travelling and attending health care). In order to calculate the mean cost, taking into account participant and carer costs, these additional direct and indirect costs will be added to the estimated NHS costs. The cost of time away from usual activities due to ill health will be estimated and valued at the national median wage rate per hour (21) if the patient or caregiver missed paid work. If the patient or caregiver reported that they had lost leisure time, this will be valued at the Department of Transport cost per hour of leisure time (22).

Data on costs for each trial participant will be summed to produce total costs for each individual, overall and also to the patients and their families. Analyses will be carried out from the perspective of the NHS and PSS, but we will also take a societal perspective by including costs borne by the participants and their families.

### 8.1.2. Assessment of health-related quality of life

The within-trial analysis will compare changes in health-related quality of life (HRQoL), based on responses to the EQ-5D-5L (23) and the SF-36 (25), at baseline, and at 1, 3, 6, 9, 12, 18 and 24 months post-consent. The EQ-5D is widely acknowledged as an appropriate tool for measuring health status and has been used to assess HRQoL in previous studies (13). However, there is a concern that this instrument might not be sensitive enough in this patient population and so, the SF-36 will also be administered. As the study population includes both children and adults, age appropriate versions of the aforementioned instruments will be used, where appropriate, along with proxy versions for very young patients. The decision of the optimal instrument, SF-36, was made following consultation with the patient group involved in the project. The data from the EQ-5D-5L and SF-36 will be combined with study participant's mortality to estimate QALYs (26).

### 8.1.3. Economic analyses

As mentioned previously, two separate analyses will be conducted:

(1) A within-trial analysis to assess the costs and outcomes associated with successful withdrawal from Eculizumab, and the costs and outcomes associated with standard care, over a two-year time horizon.
    Due to the small sample size, it is not the intention to conduct a formal economic evaluation based on the trial data alone. Rather, the within-trial analysis will focus on estimating cost and quality of life information for patients who withdraw, and who do not withdraw, from treatment. The focus of the analysis will be on understanding the key determinants of any differences in cost and quality of life

between the two patient groups. Differences may be driven by factors such as relapse, adverse events experienced and the associated required utilisation of health care services. Information derived from the within-trial assessment will be used to help populate the long-term model.

(2) Economic model: The time horizon of the trial will not capture all of the costs and health outcomes associated with withdrawal from Eculizumab. The trial itself, while providing useful data, is not an appropriate basis for a within-trial economic evaluation, given the small sample size. Therefore, an economic decision model will be developed to extrapolate costs and outcomes over the lifetime of the patient. Results of the model will be presented in terms of cost per QALY gained. The design of the model will be consistent with good practice guidelines (28). The data from the trial will be the main source of data for the economic model, but further data will be systematically derived from the literature.

The model will be developed in accordance with the NICE reference case (NICE technology appraisal guide (26)) and will characterise patients' treatment pathways and the impact of alternative strategies. At this stage, we anticipate that the model will take the form of a Markov-type, state transition model. This is likely to be most appropriate model type for this decision problem as this form of model allows the representation of a clinical situation where patients can move between health states or experience recurrent events over a long period of time. The precise structure of the model will be developed during the project and will reflect the clinical decision question and the course of the condition.

# 9 WITHDRAWN MEDICATION

## 9.1 Name and Description of withdrawn Medication

### 9.1.1 Eculizumab

Eculizumab is a humanised monoclonal antibody directed against complement component 5. It is licenced for treatment of aHUS and paroxysmal nocturnal haemoglobinuria. For aHUS, the licence is for continuous treatment once initiated.

### 9.1.2 Access to Eculizumab

Eculizumab is available to all sites in the UK within 24 hours to permit rapid treatment of active disease. This will be organized by the local site or coordinating centre. If patients are travelling overseas, we will make arrangements with the destination country to access Eculizumab if required, with arrangements from the UK commissioning authority.

## 9.2 Relapse Information

The withdrawal of Eculizumab treatment could lead to a relapse of aHUS and complications associated with both relapse and failure to re-initiate treatment within 24 hours. Relapse should be considered if:

1. The participant presents with symptoms or signs that would be consistent with the development of a TMA including neurological symptoms signs or of renal disease (macroscopic haematuria, fluid retention, fatigue)
2. The participant presents with any unexpected illness
3. Evidence of thrombocytopenia, microangiopathic haemolytic anaemia or deterioration in renal function at a study visit
4. Increased haematuria detected by urinalysis

This should lead to a full assessment for the TMA which will be identified by the presence of:

1. Thrombocytopenia
2. Microangiopathic haemolysis (fall in haemoglobin, rise in LDH, red cell fragmentation)
3. Deterioration in renal function

## 9.3 Dosage Schedule

If re-introduction of Eculizumab is required, this should follow the standard dosing regime.

## 9.4 Concomitant Medications

**Meningococcal prophylaxis**

Participants will continue with prophylactic antibiotics for 4 weeks after the last dose of Eculizumab prior to withdrawal to reduce risk of meningococcal infection. If re-introduction of Eculizumab is required the meningococcal prophylaxis will also be re-started as per the standard protocols.

# 10. PHARMACOVIGILANCE

## 10.1 Definitions

| Term | Definition |
|---|---|
| Adverse Event (AE) | Any untoward medical occurrence in a participant to whom a medicinal product has been withdrawn including occurrences which are not necessarily caused by or related to withdrawal of that product. |
| Adverse Reaction (AR) | An untoward or unintended response in a participant to an investigational medicinal product which is related to any dose administered to that participant.<br><br>The phrase “response to an investigational medicinal product” means that a causal relationship between a trial medication and an AE is at least a reasonable possibility i.e. the relationship cannot be ruled out.<br><br>All cases judged as having a reasonable suspected causal relationship to the medication qualify as adverse reactions. |
| Reference Safety Information (RSI) | The RSI is a list of medical terms detailing the ARs that are expected for a medicinal product and must be referred to when assessing a SAR for expectedness. |
| Serious Adverse Event (SAE) | A serious adverse event is any untoward medical occurrence that:<br><br>• Results in death<br>• Is life-threatening*<br>• Requires inpatient hospitalisation or prolongation of existing hospitalisation<br>• Results in persistent or significant disability/incapacity<br>• Consists of a congenital anomaly or birth defect<br>• Other important medical events that jeopardise the participant or require intervention to prevent one of the above consequences<br><br>* - life-threatening refers to an event in which the participant was at <u>immediate</u> risk of death at the time of the event; it does not refer to an event which hypothetically might have caused death if it were more severe. |
| Serious Adverse Reaction (SAR) | An adverse event that is both serious and, in the opinion of the reporting Investigator, believed with reasonable probability to be due to the trial treatment, based upon the information provided. |
| Suspected Unexpected Serious Adverse Reaction (SUSAR) | A serious adverse reaction, the nature and severity of which is not consistent with the approved Reference Safety Information. |

## 10.2 Recording and Reporting AEs and SAEs

All AEs occurring from the point of withdrawal (day 0) to end of study participation must be recorded in the study database eCRF as well as the participant's medical notes.

SAEs occurring from the point of withdrawal (day 0) must be reported to NCTU within 24 hours of the site becoming aware of the event. Any SAE that comes to the attention of the site team must be recorded and reported up until the final study visit for each participant.

For each SAE the following information will be collected:

- Full details in medical terms and case description
- Event duration (start and end dates, if applicable)
- Action taken
- Outcome
- Seriousness criteria
- Causality in the opinion of the investigator
- Whether the event is considered expected or unexpected in accordance with the approved Reference Safety Information if a causal relationship is suspected

Any change of condition or other follow-up information should be submitted to the NCTU as soon as it is available or at least within 24 hours of the information becoming available. Events will be followed up until the event has resolved or a final outcome has been reached.

## 10.3 Recording and Reporting SUSARs

SUSARs are reportable only for those patients who are withdrawn from Eculizumab and are subsequently put back on to drug during their participation in the trial. All SUSARs occurring from the first day that a patient has their Eculizumab treatment restarted until the last day of their follow up period (24 months post initial termination of Eculizumab) must be reported to the Medicines and Healthcare products Regulatory Agency (MHRA) and REC. The NCTU will perform this reporting.

The assessment of expectedness will be performed by the PI at site against the approved Reference Safety Information (RSI) for the trial (Section 4.8 of the Soliris SmPC).

Fatal and life-threatening SUSARS must be reported to the MHRA no later than 7 calendar days after the Sponsor has first knowledge of the event. Any relevant follow-up information must be sought and reported within a further 8 calendar days.

Non-fatal or non life-threatening SUSARs must be reported to the MHRA no later than 15 calendar days after the Sponsor has first knowledge of the event. Any relevant follow-up information should be sought and reported as soon as possible after the initial report.

As soon as a site suspects that a SAR may be a SUSAR they must contact the CI, sponsor representative and the trial manager immediately. The reporting timeframe starts at day 0 when the NCTU is in receipt of a minimum set of information:

- Sponsor trial reference and trial name (sponsor reference)
- EudraCT number
- Patient trial number and date of birth
- Date of notification of the event
- Medical description of the event
- Date and time of the onset of the event (including event end date if applicable)
- Causality assessment
- Seriousness of the event, particularly if life threatening or fatal
- An identifiable reporter (e.g., Principal Investigator)

This information must be provided on the SAE Form provided to site. The site is expected to fully cooperate with the NCTU in order that a full and detailed report can be submitted to the MHRA and REC within the required timelines.

PIs will be informed of all SUSARs by the C.I or member of the central clinical trial team.

## 10.4 Responsibilities

***Principal Investigator***

- *Checking for AEs and ARs when participants attend for treatment or follow-up*
- *Using medical judgement in assigning seriousness and causality and providing an opinion on expectedness of events using the Reference Safety Information approved for the trial.*
- *Ensuring that all SAEs and SARs, including SUSARs, are recorded and reported to the Sponsor within 24 hours of becoming aware of the event and provide further follow-up information as soon as available.*
- *Ensuring that AEs and ARs are recorded and reported to the Sponsor in line with the requirements of the protocol.*

***Chief Investigator***

- *Clinical oversight of the safety of trial participants, including an ongoing review of the risk/benefit.*
- *Using medical judgement in assigning seriousness, causality and expectedness of SAEs where it has not been possible to obtain local medical assessment.*
- *Using medical judgement in assigning expectedness to SARs.*
- *Immediate review of all SUSARs.*
- *Review of specific SAEs and SARs in accordance with the trial risk assessment and protocol.*

***Sponsor***

- *Assessment of expectedness of any SUSARs*
- *Expedited reporting of SUSARs to the CA and REC within required timelines*
- *Notification of all investigator sites of any SUSAR that occurs*

***TSC/DMC***

- *Review of safety data collected to date to identify any trends*

## 10.5 Notification of Deaths

All deaths will be reported as SAE's irrespective of the cause of death and reported to the NCTU following the process outlined in section 10.2. All deaths will be reported to the trial oversight committee.

## 10.6 Pregnancy Reporting

In the event of a study participant becoming pregnant on study the site must notify NCTU, the Chief Investigator and the sponsor representative within 24 hours of becoming aware of the pregnancy.

Site must approach the study participant to obtain consent to follow the pregnancy to completion.

## 10.7 Reporting Urgent Safety Measures

An Urgent Safety Measure (USM) is an action that the Sponsor or an Investigator may take in order to protect the subjects of a trial against any immediate hazard to their health or safety. Upon implementation of an USM by an Investigator, the Sponsor/CI/NCTU must be notified immediately and details of the USM given. The Sponsor/CI/NCTU must inform the MHRA and the NHS REC within 3 days of the USM taking place in accordance with the Sponsor's/NCTU's standard operating procedures.

## 10.8 Development Safety Update Reports

A Development Safety Update Report (DSUR) must be submitted to the MHRA and NHS REC once a year on the anniversary of the Development International Birth Date (DIBD). The Trial Manager must ensure that the report is submitted within 60 days of the end of the reporting period. The Trial Management Group must input into the compilation of the DSUR and the CI must review and authorise the final report before it is ready for submission. The DSUR should also be reviewed by the NCTU QA Manager and sponsor Representative prior to submission via the Common European Submission Portal (CESP) system.

# 11 STATISTICAL CONSIDERATIONS

## 11.1 Analysis Population

All consented patients who withdraw from Eculizumab will be included in the statistical analyses.

## 11.2 Statistical Analyses

A detailed Statistical Analysis Plan (SAP) will be finalised giving full detail of the planned analyses in advance of these being undertaken. These analyses are summarised below.

### 11.2.1 Analysis of the Primary Outcome Measure

This is a single arm, open label trial with the primary endpoint being a binary response (the presence/absence of a primary outcome event (TMA related SAEs as defined in section 3.3) within the follow-up period). We will compare the rate of serious events following the withdrawal of medication in the study to that expected under standard care. A maximum of 30 patients will be recruited; this is judged to be a feasible level of recruitment in a reasonable timeframe given the rare nature of the disease. There is no allowance for loss to follow-up as this patient group is already subject to a high degree of clinical follow-up and death is defined as one of the serious events under consideration.

The Bayes factor single arm binary model (4) will be used to monitor the trial. Based on historical data, the event rate for the standard of care is 0.06, and we expect that withdrawal of the treatment would give a response rate of 0.12. This choice of an acceptable event rate has been informed in discussion with patients. Using this Bayesian hypothesis test-based design, we assume the serious event rate is 0.06 under the null hypothesis, and the event rate is 0.12 under the alternative hypothesis.

We assume that the sample distribution of number of responses follows a binomial distribution, and use an inverse moment prior for response under the alternative hypothesis.

**Stopping Rules**

A minimum of 5 patients will be enrolled before applying the stopping rules, and the cohort size for monitoring the trial is 5 patients. The DMC can request earlier review if adverse events are reported before this point.

We implement two stopping rules during the trial:

(1) We will stop the trial for superiority (there being fewer serious events on the intervention than would be expected under standard of care) if the posterior probability of the alternative hypothesis is less than 0.05, i.e. $Pr(H1|Data) < 0.05$;

(2) We will stop the trial for inferiority if the posterior probability of the alternative hypothesis is greater than 0.80, i.e., Pr(H1|Data) > 0.80.

**Operating Characteristics**

The operating characteristics of the design were produced using the M. D. Anderson Cancer Center Department of Biostatistics software BayesFactorBinary, version 1.0 (https://biostatistics.mdanderson.org/SoftwareDownload/ProductSupportFiles/BayesFactorBinary/UsersGuide_Bayhttps://biostatistics.mdanderson.org/SoftwareDownload/ProductSupportFiles/BayesFactorBinary/UsersGuide_BayesFactorBinary.pdf).

| Scenario | True rate of serious events | Probability of Stopping for Inferiority | Probability of Stopping for Superiority | Average number of patients treated (Percentiles: 10%, 25%, 50%, 75%, 90%) |
|---|---|---|---|---|
| **1** | 0.06 | 0.096 | 0 | 28.44 (30, 30, 30, 30, 30 ) |
| **2** | 0.12 | 0.443 | 0 | 23.48 (5, 15, 30, 30, 30 ) |
| **3** | 0.18 | 0.753 | 0 | 17.76 (5, 10, 15, 30, 30 ) |
| **4** | 0.24 | 0.928 | 0 | 13.72 (5, 5, 15, 15, 30 ) |
| **5** | 0.30 | 0.982 | 0 | 10.52 (5, 5, 10, 15, 20 ) |

For example, if the true rate of serious events is 0.06 (Scenario 1, the null hypothesis), the trial will stop with probabilities of 0.096 and 0 in favour of the alternative and null hypotheses, respectively. The average number of patients (10%, 90% percentiles) treated is 28.44 (30, 30). If the true serious event rate is 0.12 (Scenario 2, the alternative hypothesis), the trial will stop with probabilities of 0.443 and 0 in favour of the alternative and null hypotheses, respectively. The average number of patients (10%, 90%) treated is 23.48 (5, 30).

**Stopping Boundaries**

The stopping boundaries of the design were produced using the M. D. Anderson Cancer Center Department of Biostatistics software **BayesFactorBinary**, version 1.0.

| Number of patients (in complete cohorts of 5) | **Stop** the trial for Superiority if there are this many **Events** (inclusive) | **Continue** the trial if there are this many **Events** (inclusive) | **Stop** the trial for Inferiority if there are this many **Events** (inclusive) |
|---|---|---|---|
| 5 | Never stop for superiority with this many patients | 0-1 | 2-5 |
| 10 or 15 | Never stop for superiority with this many patients | 0-2 | 3-15 |
| 20 | Never stop for superiority with this many patients | 0-3 | 4-20 |

| | | | |
|---|---|---|---|
| 25 or 30 | Never stop for superiority with this many patients | 0-4 (The trial always stops at 30 patients, which is the maximum) | 5-30 |

We have taken the conservative approach of stopping the study on the grounds of inferiority should there be 2 serious events in the first cohort of 5 patients. Subsequent to this, the study would stop should there be 3 or more serious events observed in the first 15 patients, 4 or more in the first 20 patients, and 5 or more in the whole study population during follow-up.

Therefore, we are well placed to quickly respond to any negative safety signal from the emerging data.

These numbers have been obtained using 1000 repetitions in the software simulation. These calculations have also been repeated using different numbers of repetitions; in these cases the resulting stopping boundaries are unchanged from those quoted above while the probability of stopping for inferiority and the average number of patients treated are marginally changed.

It is acknowledged that there may well be differing risk of relapse according to disease aetiology however, the available numbers do not allow for risk strata to be monitored separately. The Data Monitoring Committee (DMC) will consider the issue of risk based on disease aetiology as part of their remit.

In addition to this ongoing analysis, at the end of the study, the primary outcome data will also be reported descriptively, together with the number of patients recruited. Descriptive statistics reported will be selected from mean, median, standard deviation, range and inter-quartile range as appropriate to the specific outcome measure. For proportion outcomes, the number of patients recording the event will be reported together with this value.

Due to small the sample size, no comparative statistical methods will be applied. There will be no imputation of any missing data and analysis will take the form of a complete case analysis.

**11.2.2. Analysis of Secondary Outcome Measures**

In line with the primary outcome data, at the end of the study, the secondary outcome data will also be reported descriptively, together with the number of patients recruited. The same descriptive statistics and restrictions to the analysis will be considered as for the primary outcome.

**11.2.3. Subgroup Analyses**

With the exception of the analysis of the primary outcome on an ongoing basis, the analyses described above may be reported separately for different genetic groups or risk strata.

## 11.3. Health economic data analysis

### 11.3.1. Analysis Population

The analysis population will consist of the 30 patients who have consent to withdraw from Eculizumab, as well an additional 20 patients who do not withdraw but who agree to take part in the health economic component of the study. These additional 20 patients will serve as a comparator group for the economic assessment.

### 11.3.2. Within-trial assessments of costs and outcomes

Costs and health outcomes (measured in terms of adverse events and QALYs) associated with Eculizumab withdrawal (full and part), compared with standard care, will be assessed over the 24-month follow-up period. Information on costs and health outcomes will be recorded for each individual involved in both treatment groups. Data derived from the within-trial analysis will be assessed to understand the key determinants of differences in costs and outcomes between the two patient groups. Data will then be used to parameterise the definitive economic model (combined with data from the literature).

### 11.3.3. Assessment of cost-effectiveness

An economic decision model will be developed to assess the cost-effectiveness of the alternative treatment options under evaluation. Costs and health consequences, measured in terms of QALYs, associated with Eculizumab withdrawal, and a policy of monitoring following withdrawal, and standard care, beyond the two-year timeframe of the trial will be captured. We propose to conduct a cost-utility analysis, with results presented in terms of incremental cost per QALY gained.

The data from the trial will be the main source of data for the economic model, but further data with which to model outcomes beyond the two-year follow-up will be derived from the literature (1-3) and other existing data sources, following guidance for best practice (28). These data will include information on factors such as adverse events after Eculizumab withdrawal and relapse after Eculizumab withdrawal. The economic evaluation will be carried out from a UK NHS and PSS perspective, to take into account health care costs and longer-term social care costs. Both costs and QALYs will be discounted in the base case at 3.5% per year (NICE technology appraisal guide (26)). In order to characterise the uncertainty in the data used to populate the model, both deterministic and probabilistic sensitivity analyses will be conducted. Sensitivity analysis will focus on key structural and parameter uncertainties in the economic model. It will also explore potential variation in the implementation of patient monitoring and surveillance following withdrawal from treatment. Variation in the frequency, and intensity, of monitoring will be assessed.

### 11.4. Sample Size Calculations

A maximum of 30 patients will be recruited to the withdrawal study; this is judged to be a feasible level of recruitment in a reasonable timeframe given the rare nature of the disease. The specifics of this sample size are intrinsically linked to the analysis methodology of the primary outcome measure and as such the justification is included in the earlier section of the protocol (section 11.2.1). There is no allowance for loss to follow-up as this patient group is already subject to a high degree of clinical follow-up and death is defined as one of the serious events under consideration.

# 12. DATA HANDLING

## 12.1. Data Collection Tools and Source Document Identification

All the data required will be recorded in clinical notes as part of routine clinical procedures. Data for individual patients will be transferred by each PI or his/her delegated nominee to the eCRF (the secure, validated clinical data management system, MACRO) as soon as possible following each procedure (see 7.2). Similarly, data will be entered into the eCRF following any non-scheduled visits. Patients will record results of urinalysis in a patient-held diary which will be reviewed, copied and data entered into the eCRF by study staff. Patient identification on the eCRF will be via a unique study identifier number assigned to the patient at site once the patient has consented. A record linking the patient's name to the unique study identifier number will be held only in a locked room at the study site, and is the responsibility of the PI. As such, patients cannot be identified from eCRFs. The CI, or nominated designee, will continually monitor completeness and quality of data recording in CRFs and will correspond regularly with site PIs (or their delegated assistants) to capture any missing data where possible, and ensure continuous high-quality data.

The CDMS (Elsevier's MACRO) used for this trial is fully compliant with all regulatory frameworks for research of this nature. It uses a secure web-based interface for data entry; no data are stored on computers at site. MACRO users are assigned role based permissions specific to their site and role. The system has an inbuilt back-up facility, through Elsevier's hosting partner Rackspace's secure premises in London, and is managed and supported by the Rackspace team.

## 12.2. Data Handling and Record Keeping

All of the data collected will be kept strictly confidential and will be managed in accordance with the General Data Protection Regulation (GDPR). Identifiable data will be stored in a separate, limited-access database. Paper copies of study-related documentation will be annotated, signed and dated, and filed in the medical notes. The overall quality and retention of study data is the responsibility of the Chief Investigator. All study data will be retained in accordance with the latest Directive on GCP (2005/28/EC) and local policy.

### 12.3. Access to Data

Clinical information will not be released without the written permission of the participant, except as necessary for monitoring and auditing by the Sponsor, its designee, Regulatory Authorities, the Data Monitoring Committee (DMC) or the REC. Secure, anonymised, electronic data will be released to the Study Statistician and Health Economist for analysis. The PI and study site staff may not disclose or use for any purpose other than performance of the study, any data, record, or other unpublished, confidential information to which they have access, in order to carry out the study. Prior written agreement from the Sponsor or its designee must be obtained for the disclosure of any confidential information to other parties.

### 12.4. Archiving

Archiving of trial data transferred by eCRFs will be carried out according to NCTU and Sponsor SOPs. Routine clinical notes will be archived according to NHS Trust regulations at each site. All data will be retained for 5 years.

# 13. MONITORING, AUDIT & INSPECTION

The trial will be managed through the Newcastle Clinical Trials Unit. The study will be co-ordinated by a Trial Management Group (TMG) that will include those individuals responsible for the day-to-day management of the trial. The TMG will monitor all aspects of the conduct and progress of the study, ensure that the protocol is adhered to and take appropriate action to safeguard participants and the quality of the study itself. TMG meetings will occur at least monthly and include a teleconference link to other trial sites, where required. Progress will be monitored proactively according to timelines and any issues addressed. The TMG will liaise with the Trial Steering Committee (TSC), providing updates on trial progress and highlighting any issues arising.
The Principal Investigators will be responsible for highlighting day-to-day study conduct at each site. The NCTU will provide day-to-day support for the sites and training, via Investigator meetings, site initiation visits and routine monitoring visits.

Quality control will be maintained through adherence to NCTU SOPS, Newcastle Joint Research Office SOPs, study protocol, GCP principles, research governance and clinical trial regulations.

An independent Data Monitoring Committee (DMC) will be established to oversee treatment outcomes during the study. During the trial, interim analyses of baseline, follow-up data and any other analyses requested by the committee, will be supplied, in strict confidence, to the DMC chair. The DMC will include an independent statistician. This committee will monitor efficacy, safety and clinical outcomes. At the first meeting, the DMC will agree on its charter of operation, and discuss and advise on the inclusion of an interim analysis and possible adoption of a formal stopping rule for efficacy or safety.

A Trial Steering Committee (TSC) will be established to provide overall –independent oversight of the trial, and will oversee trial conduct and progress. The Chair will be an independent consultant, and the committee will include one lay member. Members of the Trial Management Group (TMG) will attend these meetings. The TSC terms of reference and members' names and contact details will be published ahead of its first meeting. At the first meeting, the TSC will agree on its charter of operation The committee will meet at least annually for the duration of the trial.

Clinical management of participants will remain subject to individual sites internal audit procedures. The Newcastle CTU will carry out monitoring to ensure appropriate study conduct and data collection. Electronic data will be stored on secure, password-protected computers. NCTU staff will use a combination of central review and site monitoring visits to ensure the study is conducted in accordance with GCP. The trial manager will conduct the site monitoring and will require access to the patients' clinical notes and consent forms.

The following will be monitored:

- Presence of completed original consent forms in the Investigator Site File (ISF) and copies in patients' notes.
- Existence of patients, by comparison of original consent forms with patient identification (enrolment) list.
- Reported serious adverse events, by verification against patient notes (source data verification).
- Presence of essential documents in the ISF and study files.
- Primary endpoint data for a percentage of study participants, by source data verification.
- Applications for study authorisations and submissions of progress/safety reports, for accuracy and completeness, prior to submission.
- Eligibility data for a percentage of study participants, by source data verification.

All monitoring findings will be reported and followed up with the appropriate persons in a timely manner. The study may be subject to inspection and audit by The Newcastle upon Tyne Hospitals NHS Foundation Trust under its remit as Sponsor, and other regulatory bodies, to ensure adherence to GCP. The investigators and institutions will permit study-related monitoring, audits, REC review and regulatory inspection(s), providing direct access to source data and documents relating to the study.

# 14. ETHICAL AND REGULATORY CONSIDERATIONS

## 14.1. Research Ethics Committee Review and Reports

The NCTU will obtain a favourable ethical opinion from an NHS Research Ethics Committee (REC) prior to the start of the trial. All parties will conduct the trial in accordance with this ethical opinion.

The NCTU will notify the REC of all required substantial amendments to the trial that result in a change to trial documentation (e.g. protocol or patient information sheet). Substantial amendments that require a REC favourable opinion will not be implemented until this REC favourable opinion is obtained. The NCTU will notify the REC of any serious breaches of GCP or the protocol, urgent safety measures or SUSARs that occur during the trial.

An annual progress report will be submitted each year to the REC by NCTU until the end of the trial. This report will be submitted within 30 days of the anniversary date on which the original favourable ethical opinion was granted.

The NCTU will notify the REC of the early termination or end of trial in accordance with the required timelines.

## 14.2. Peer Review

The trial protocol has been discussed with experts in the management of aHUS in the UK and in other countries who have experience of withdrawing Eculizumab. The internal team includes clinicians with experience in the treatment of aHUS and conducting multicentre clinical trials in aHUS and other diseases. The team has a diverse background including co-applicants with expertise in trial design and analysis, qualitative research, social sciences and health economics. The funding process included external peer review. The applicants responded to the peer review reports and the funding was awarded.
The protocol described is considered sufficient to detect relapse and similar protocols are under development in other countries with the expectation that withdrawal can be undertaken safely.

## 14.3. Public and Patient Involvement

Patients and the patient group (aHUSUK) have been involved in trial design and dissemination of information about the trial and collection of patient feedback. The acceptable level of monitoring was discussed with patients and has been altered to reflect their suggestions. For example, we will maximize flexibility for study visits and, in case of problems during the withdrawal phase, we will provide written patient information and access to the aHUS National Service.

## 14.4. Regulatory Compliance

The trial will be conducted in accordance with the Medicines for Human Use (Clinical Trials) Regulations 2004 and subsequent amendments. All parties must abide by these regulations and the ICH GCP guidelines.

The NCTU will obtain a Clinical Trial Authorisation (CTA) from the MHRA prior to the start of the trial and will notify the MHRA of any substantial amendments that require review by the competent authority. These substantial amendments will not be implemented until the MHRA have issued an acceptance of the amendment.

The NCTU will notify the MHRA of any serious breaches of GCP or the protocol, urgent safety measures or SUSARs that occur during the trial.

The Development Safety Update Report will be submitted each year to the MHRA by the NCTU until the end of the trial.

The NCTU will notify the MHRA of the early termination or end of trial in accordance with the required timelines.

## 14.5. Protocol Compliance

Protocol deviations, non-compliances and breaches are departures from the approved protocol. Unintentional protocol deviations will be documented and reported to the CI and sponsor. Where necessary, Corrective and Preventative Actions (CAPA) will be implemented. These will also be documented and reported to the CI and sponsor. Deviations found to frequently recur at a site are not acceptable and could be classified as a serious breach.

## 14.6. Notification of Serious Breaches to GCP and/or the Protocol

A serious breach is a breach that is likely to effect to a significant degree –

- the safety or physical or mental integrity of the subjects of the trial; or
- the scientific value of the trial

The sponsor must be notified immediately of any incident that may be classified as a serious breach. The NCTU will notify the MHRA and the NHS REC within the required timelines in accordance with the NCTU SOP.

## 14.7. Data Protection and Patient Confidentiality

Personal data will be regarded as strictly confidential. All data retained at site and sent electronically to the main co-ordinating centre will contain Study ID and initials only. The secure password-protected eCRF database (MACRO) also requires initials and date of birth. This is essential for participant identification and verification. This information is also required for the fax-to-email SAE reporting system.

All personnel with access to trial data are qualified and trained in, and will comply with ICH GCP. Justification for all such electronic transmissions will be approved by Sponsor and covered in the Caldicott applications made locally at each site.

A Participant Identification List will be the only document retained within the ISF, which contains full details of hospital number, patient name, and study ID.

The study will comply with the Data Protection Act, 1998. All study records and Investigator Site Files will be kept at site in a locked filing cabinet with restricted access to those who are named on the SETS aHUS delegation log at each Site.

## 14.8. Indemnity

The NHS Trust has liability for clinical negligence that harms individuals toward whom they have a duty of care. NHS Indemnity covers NHS staff and medical academic staff with honorary contracts conducting the trial for potential liability with respect to negligent harm arising from the conduct of the study. The Newcastle upon Tyne Hospitals NHS Foundation Trust is the Sponsor and through the Sponsor, NHS indemnity is provided with respect to potential liability and negligent harm arising from study management. Indemnity, with respect

to potential liability arising from negligent harm related to study design, is provided by NHS schemes for those protocol authors who have their substantive contracts of employment with the NHS, and by Newcastle University Insurance schemes for those protocol authors who have their substantive contract of employment with Newcastle University. This is a non-commercial study and there are no arrangements for non-negligent compensation.

### 14.9. Amendments

It is the responsibility of the Research Sponsor to determine if an amendment is substantial or not and study procedures must not be changed without the mutual agreement of the CI, Sponsor and the Trial Management Group.

Substantial amendments will be submitted to the REC and/or MHRA (as appropriate) and will not be implemented until this approval is in place. It is the responsibility of the NCTU to submit substantial amendments.

Non-substantial amendments will be submitted to the Health Research Authority (HRA) and will not be implemented until authorisation is received.

Substantial amendments and those minor amendments which may impact sites will be submitted to the relevant NHS R&D Departments for notification to determine if the amendment affects the NHS permission for that site.  Amendment documentation will provide to sites by the NCTU.

### 14.10. Post-Trial Care

After the Trial, patients will be followed up according to the routine review procedures within the clinic. No additional follow-up is required in relation to the Trial. Those patients who withdrew from Eculizumab may remain withdrawn or return to treatment depending on their preference and clinical opinion.

### 14.11. Access to the Final Trial Dataset

Until publication of the Trial results, access to the full dataset will be limited to the Project Management Group and to authors of the publication. If not co-authors, the leads for clinical sites will be given access to the full dataset from all participating Centres (anonymised) that is included in the Trial publication.

## 15. DISSEMINATION POLICY

Data will be the property of the Chief Investigator and Co-Investigators. Participating centres must not present or publish any data or analysis of data related to their participation in the study without consent of the Trial Management Group and the funder.
On completion of the Trial, data will be analysed and a Final Study report prepared.
Publication will be reviewed by the Trial Management Group in partnership with the Chief Investigator and authorship agreed with the Co-Investigators and Principal Investigators.

Publication of results will require preceding consent from the funder according to the terms of the award.

Final outcomes will be disseminated at relevant conferences by platform and poster presentations. The findings will be submitted for publication in peer reviewed journals. Results will also be reported to the Sponsor and Funder, and will be available on their websites. Manuscripts, abstracts and other modes of presentation will be reviewed by the Trial Steering Committee and Funder prior to submission. Individual patients will not be identifiable in any study report.

We will feed back to centres via newsletters, trial close down meetings and publications. Feedback in the form of a lay summary will be provided to participants via a participant-specific newsletter at the end of trial (if they have indicated on the consent form that they wish to receive it) and by wider publicity generated. Results will also be available at the end of the study on the atypical HUS website (http://www.atypicalhus.co.uk/).

# 16. REFERENCES

1. Ardissino G, Possenti I, Tel F, Testa S, Salardi S, Ladisa V. Discontinuation of Eculizumab Treatment in Atypical Hemolytic Uremic Syndrome: An Update. Am J Kidney Dis. 2015;66(1):172-3.
2. Ardissino G, Testa S, Possenti I, Tel F, Paglialonga F, Salardi S, et al. Discontinuation of eculizumab maintenance treatment for atypical hemolytic uremic syndrome: a report of 10 cases. Am J Kidney Dis. 2014;64(4):633-7.
3. NICE. https://www.nice.org.uk/guidance/hst1/resources/eculizumab-for-treating-atypical-haemolytic-uraemic-syndrome-1394895848389 2015 [
4. Johnson VE, Cook JD. Bayesian design of single-arm phase II clinical trials with continuous monitoring. Clin Trials. 2009;6(3):217-26.
5. Fremeaux-Bacchi V, Fakhouri F, Garnier A, Bienaime F, Dragon-Durey MA, Ngo S, et al. Genetics and outcome of atypical hemolytic uremic syndrome: a nationwide French series comparing children and adults. Clin J Am Soc Nephrol. 2013;8(4):554-62.
6. Noris M, Remuzzi G. Thrombotic microangiopathy after kidney transplantation. Am J Transplant. 2010;10(7):1517-23.
7. Johnson S, Stojanovic J, Ariceta G, Bitzan M, Besbas N, Frieling M, et al. An audit analysis of a guideline for the investigation and initial therapy of diarrhea negative (atypical) hemolytic uremic syndrome. Pediatric nephrology (Berlin, Germany). 2014;29(10):1967-78.
8. Sheerin NS, Kavanagh D, Goodship TH, Johnson S. A national specialized service in England for atypical haemolytic uraemic syndrome-the first year's experience. QJM. 2016;109(1):27-33.
9. Warwicker P, Goodship TH, Donne RL, Pirson Y, Nicholls A, Ward RM, et al. Genetic studies into inherited and sporadic hemolytic uremic syndrome. Kidney international. 1998;53(4):836-44.
10. Noris M, Caprioli J, Bresin E, Mossali C, Pianetti G, Gamba S, et al. Relative role of genetic complement abnormalities in sporadic and familial aHUS and their impact on clinical phenotype. Clin J Am Soc Nephrol. 2010;5(10):1844-59.
11. Le Quintrec M, Zuber J, Moulin B, Kamar N, Jablonski M, Lionet A, et al. Complement genes strongly predict recurrence and graft outcome in adult renal transplant recipients with atypical hemolytic and uremic syndrome. Am J Transplant. 2013;13(3):663-75.
12. Gruppo RA, Rother RP. Eculizumab for congenital atypical hemolytic-uremic syndrome. The New England journal of medicine. 2009;360(5):544-6.

13. Legendre CM, Licht C, Muus P, Greenbaum LA, Babu S, Bedrosian C, et al. Terminal complement inhibitor eculizumab in atypical hemolytic-uremic syndrome. The New England journal of medicine. 2013;368(23):2169-81.
14. Licht C, Greenbaum LA, Muus P, Babu S, Bedrosian CL, Cohen DJ, et al. Efficacy and safety of eculizumab in atypical hemolytic uremic syndrome from 2-year extensions of phase 2 studies. Kidney international. 2015;87(5):1061-73.
15. European Medicines Agency. http://www.ema.europa.eu/docs/en_GB/document_library/EPAR_-_Assessment_Report_-_Variation/human/000791/WC500119185.pdf 2011 [
16. FDA. http://www.fda.gov/AboutFDA/CentersOffices/OfficeofMedicalProductsandTobacco/CDER/ucm273089.htm [
17. Walport MJ. Complement. First of two parts. The New England journal of medicine. 2001;344(14):1058-66.
18. Carr R, Cataland SR. Relapse of aHUS after discontinuation of therapy with eculizumab in a patient with aHUS and factor H mutation. Annals of hematology. 2013;92(6):845-6.
19. Nester CM, Barbour T, de Cordoba SR, Dragon-Durey MA, Fremeaux-Bacchi V, Goodship TH, et al. Atypical aHUS: State of the art. Molecular immunology. 2015;67(1):31-42.
20. Curtis L. Unit costs of Health and Social Care. PSSRU, University of Kent, 2014.
21. Statistics OoN. Annual Survey of Hours and Earnings, 2014 Provisional Results. 2014.
22. Transport Do. TAG Unit A1.3 User and Provider Impacts. 2014.
23. Devlin N, Shah K, Feng Y, Mulhern B, Van Hout B. Valuing Health-Related Quality of Life: An EQ-5D-5L Value Set for England. Office of Health Economics. 2016.
24. McCabe C, Stevens K, Roberts J, Brazier J. Health state values for the HUI 2 descriptive system: results from a UK survey. Health Econ. 2005;14(3):231-44.
25. Brazier J, Roberts J, Deverill M. The estimation of a preference-based measure of health from the SF-36. J Health Econ. 2002;21(2):271-92.
26. NICE. Guide to the methods of technology appraisal 2013. 2013.
27. Glaser BG. The Constant Comparative Method of Qualitative Analysis. Social Problems. 1965;12(4):436-45.
28. Philips Z, Ginnelly L, Sculpher M, Claxton K, Golder S, Riemsma R, et al. Review of guidelines for good practice in decision-analytic modelling in health technology assessment. Health Technol Assess. 2004;8(36):iii-iv, ix-xi, 1-158.

# 17. APPENDICES

## 17.1 Appendix 1 - Safety Reporting Diagram

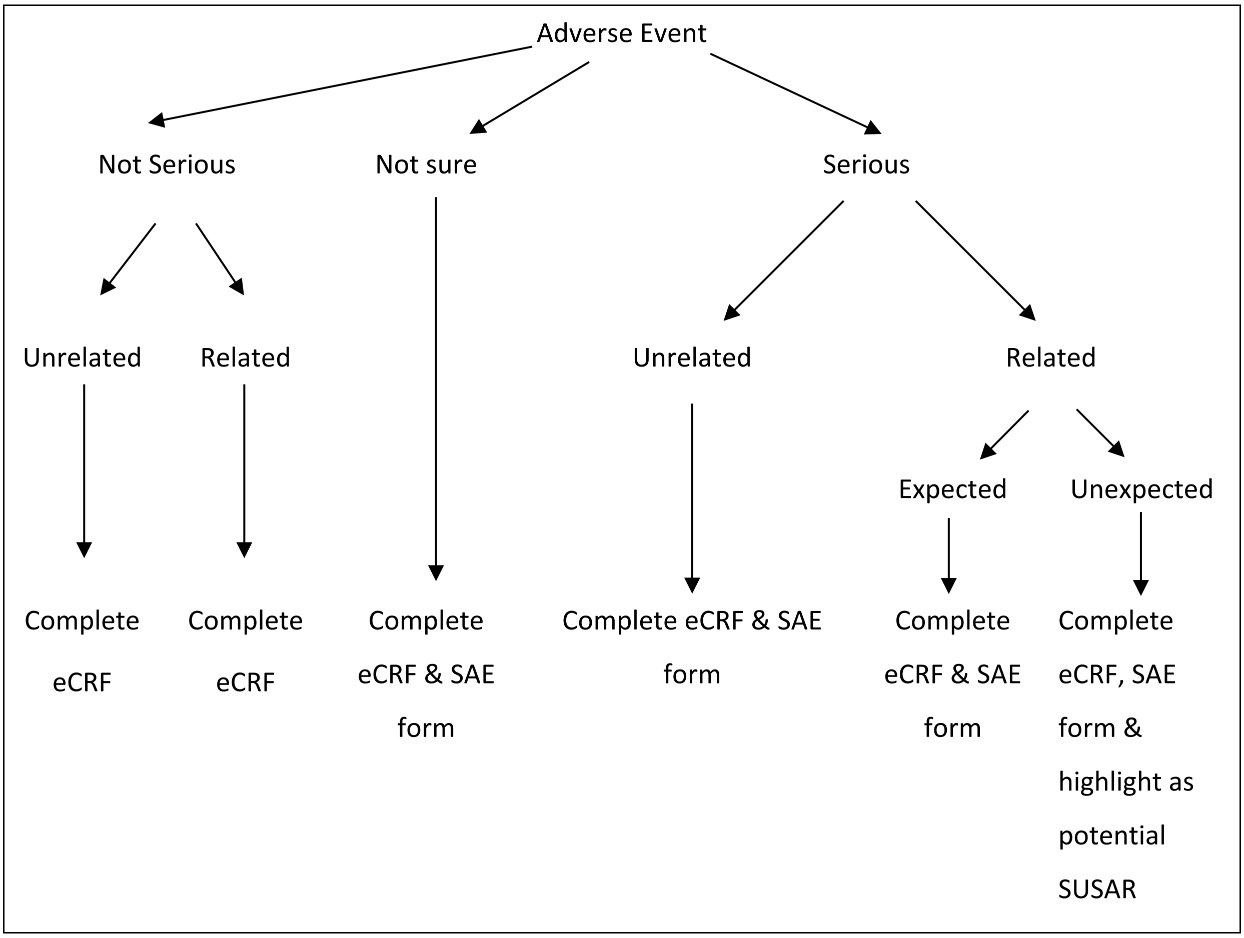


Emergency contact details for reporting SAEs

outside of normal 9am-5pm working hours please contact the Newcastle Hospitals Switchboard and request to speak to the clinician on call for the aHUS Service on +44 (0)191 233 6161

**<u>You must also</u>**

Please send SAE form(s) via secure email to nctu.sets.sae@nhs.net (NHS.net to NHS.net ONLY)

## 17.2. Appendix 2 – Amendment History

| Amendment Number | Protocol version no. | Date issued | Author(s) of changes | Details of changes made |
|---|---|---|---|---|
| SA01 | 2.0 | 10/05/2018 | Sarah Dunn | **Changes to protocol:**<br>Inclusion items that relate only to those consenting to withdraw from Eculizumab have been described as such on pg. 22. Exclusion criteria item removed due to duplication (appears in inclusion criteria) pg. 22.<br>Two Complement activation and biomarker samples added to the schedule of events (Day 1 & unscheduled visit) and 8 removed (Days 5, 12, 14, 17, 18, 20, 25 & 31). This has also been updated in the text on page 25.<br>Minor typographical errors and text omissions on pages 9 & 25 & 39.<br>Addition of reporting details to Appendix 1.<br>**Other:**<br>Additional text to the Patient Urinalysis diaries.<br>Dr Edwin Wong replaced Professor Neil Sheerin as P.I for NUTH<br>Manchester University Hospital Trust and Cambridge University Hospital Trusts have been added as sites. |
| SA02 | 3.0 | 31/08/2018 | Sarah Dunn | **Changes to protocol:**<br>Update to safety definition table and SUSAR Reporting information in section 10 |

| | | | | |
|---|---|---|---|---|
| | | | | Minor typographical errors and text omissions updated on pages 23, 25, 26 & 27. |
| SA03 | 4.0 | 10/03/2019 | Sarah Dunn | **<u>Changes to protocol:</u>**<br><br>The exclusion criteria relating to current or planned pregnancy has been updated.<br><br>Updates to the Schedule of events<br><br>Addition of PID text<br><br>Minor typographical errors and text omissions updated on pages 14, 23 and 24.<br><br>**Other:**<br><br>GDPR text added to PIS (16+ AND Legal Guardian only)<br>Typographical errors updated in HE Leal Guardian Consent.<br>Addition of Patient Invitation Letter.<br>City of Sunderland Hospitals Trust, Great Ormond Street Children's Hospital Trust and Aberdeen Royal Hospital (NHS Grampian) have been added as sites. |
| SA04 | 5.0 | 18/12/2019 | Sarah Dunn | **<u>Changes to protocol:</u>**<br><br>The exclusion criteria pertaining to all participants has been updated.<br><br>Schedule of events for Health Economics patients has been added.<br><br>Requirements for haptoglobin has been changed to non-compulsory if unavailable at site.<br><br>Process for recruiting non-English speaking patients has been added.<br><br>Urine PCR has been removed from the bullet point list for every visit. Urine PCR occurs on 8 occasions as stated in the schedule of events. |

| | | | | |
|---|---|---|---|---|
| | | | | Reference to the Data Protection Act 1998 has been removed and replaced with the General Data Protection Regulation (GDPR).<br><br>**Other**<br><br>Addition of 3 sites & change to Newcastle PI.<br><br>**RSI for trial – Soliris SmPC updated (section 4.8)** |
| NSA04 | 6.0 | 30/04/2020 | Sarah Dunn | **Changes to protocol:**<br><br>Addition of COVID-19 pandemic mitigation strategies.<br><br>Addition of PI signature page |
| SA05 | 7.0 | 14/01/2021 | Sarah Dunn | **Changes to protocol:**<br><br>Primary outcome measure definition refined (pg20-21)<br><br>Health Economic questionnaire delivery and completion updated (Pg29)<br><br>TMA related relapse identification refined (pg42) |
| NSA09 | 8.0 | 12/07/2021 | Sarah Dunn | **Changes to protocol:**<br><br>Clarification regarding contraceptive use should a female patient, withdrawing from treatment, become sexually active during the trial (pg 24).<br><br>Updated relapse reporting requirements (pg 32).<br><br>Separation of relapse management (section 3.2.3) into 2 subsections (pg 32).<br><br>Removal of SOHO66 number as method of SAE form reporting (pg 59). |
| NSA10 | 9.0 | 01/06/2023 | Ciara Kennedy | **Changes to protocol:** |

| | | | | |
|---|---|---|---|---|
| | | | | Update to the health Economics questionnaire schedule to allow for HE analysis to commence November 2023. (pg 29 & pg 39)<br><br>We have also added a footnote to the Schedule of events (pg 35) |
| NSA12 | 10.0 | 21/11/2023 | Ciara Kennedy | Updated wording in Section 10.2 of the Protocol to clarify the expected safety reporting time-frame for participants |
