# Supplementary material for: Eculizumab withdrawal and monitoring in atypical haemolytic uraemic syndrome (SETS aHUS): a multicentre, open label, prospective, single arm trial
Source: Lancet Reg Health Eur. 2025 Aug 7;56:101392. doi: 10.1016/j.lanepe.2025.101392 (PMC12811780; doi:10.1016/j.lanepe.2025.101392)
Supplement: Appendix [file mmc2.docx]

*Supplemental appendix to:*

Stopping eculizumab treatment safely in atypical haemolytic uraemic syndrome (SETS aHUS): a multicentre, open label, prospective, single arm trial

Andrew Bryant, Jan Lecouturier, Giovany Orozco-Leal, Victoria Brocklebank, Sonya Carnell, Tom Chadwick, Sarah Dunn, Sally Johnson, David Kavanagh, Ciara Kennedy, Michael Malina, Emma Montgomery, Colin Muirhead, Yemi Oluboyede, Luke Vale, Chris Weetman, Edwin Wong, Neil S Sheerin for the SETS aHUS consortium

**Table to contents**

[OBJECTIVES AND OUTCOME MEASURES 3](#_Toc184159097)

[STUDY PROTOCOL 5](#_Toc184159098)

[1. TRIAL DESIGN 5](#_Toc184159099)

[2. ELIGIBILITY CRITERIA 5](#_Toc184159100)

[3. TRIAL PROCEDURES 7](#_Toc184159101)

[4 WITHDRAWN MEDICATION 18](#_Toc184159102)

[5. PHARMACOVIGILANCE 20](#_Toc184159103)

[6. STATISTICAL CONSIDERATIONS 24](#_Toc184159104)

[7. DATA HANDLING 27](#_Toc184159105)

[8. MONITORING, AUDIT & INSPECTION 28](#_Toc184159106)

[9. ETHICAL AND REGULATORY CONSIDERATIONS 29](#_Toc184159107)

[Trial Steering Committee 33](#_Toc184159108)

[Data Monitoring Committee 33](#_Toc184159109)

[Patient and Public Involvement and Engagement 33](#_Toc184159110)

[Supplementary table 1 Patient characteristics in the eculizumab withdrawal study 34](#_Toc184159111)

[Supplementary table 2. Summary of haematology results 36](#_Toc184159112)

[Supplementary table 3: Summary of biochemistry results 47](#_Toc184159113)

[Supplementary table 4. Summary of reported Serious Adverse Events (SAEs) 69](#_Toc184159114)

[Supplementary figure 1. Biochemical and haematological parameters for patient 7 72](#_Toc184159115)

[Supplementary figure 2. Biochemical and haematological parameters for patient 8 73](#_Toc184159116)

# OBJECTIVES AND OUTCOME MEASURES

## Primary Objective

The primary clinical objective is to determine the safety of Eculizumab withdrawal in patients with aHUS.

## Secondary Objectives

*1*. Measure the effectiveness of a monitoring protocol to detect disease relapse following withdrawal of Eculizumab.

*2.* Describe the relapse rate after withdrawal of Eculizumab.

*3*. Estimate the proportion of patients, currently on long-term treatment with Eculizumab, who can be maintained off treatment.

*4.* Describe the period from withdrawal to relapse in those patients who restart treatment.

*5.* Measure the change in estimated GFR (calculated by the CKD-EPI or modified Schwartz equations) over the course of the study.

*6.* Identify important clinical and laboratory indicators of imminent relapse.

## Primary Outcome Measures

Number of patients with a TMA related Serious Adverse Event (SAE) defined as any of the following:

- Irreversible (>3 months) reduction in estimated glomerular filtration rate (eGFR) not attributable to another cause:
- by ≥20% if the screening eGFR is <90mls/min/1.73m^2^
- by >20% to a level <90mls/min/1.73m^2^ if the screening eGFR is >90mls/min/1.73m^2^

The equations used to obtain eGFR are known to be inaccurate above a value of 90 mls/min/1.73m^2^ for both adults and children. It is plausible that a ≥20% reduction in eGFR could be due to this inaccuracy or other outside clinical factors. Where an irreversible reduction in eGFR of ≥20% is observed, the case will be reviewed by the DMEC assess the causal relationship to eculizumab withdrawal

- An episode of AKI attributed to a TMA that requires renal replacement therapy.
- A non-renal manifestation of a TMA that require hospitalisation, cause irreversible organ damage or death.

## Secondary Outcome Measures

1. The effectiveness of a monitoring protocol to detect disease relapse following withdrawal of Eculizumab assessed by:

i. The proportion of patients who relapse and restart Eculizumab without the development of a TMA-related SAE (section 3.3).

ii. The time from the first clinical feature (symptom, positive urinalysis or laboratory result) of a relapse of TMA and the re-introduction of Eculizumab treatment.

2. The relapse rate after withdrawal of Eculizumab as determined by the proportion of patients who relapse after Eculizumab is withdrawn.

3. Estimate of the proportion of patients, currently on long-term treatment with Eculizumab, who can be maintained off treatment.

4. Description of the period from withdrawal to relapse in those patients who restart treatment measured from baseline (day 0) to day of re-introduction of treatment or end of the study.

5. The change in estimated GFR as calculated by the CKD-EPI or modified Schwartz equations over the course of the study from baseline (day 0) to end of the study.

6. Identification of important clinical and laboratory indicators of imminent relapse by review of reported symptoms, physical signs, urinalysis and laboratory results prior to the diagnosis of a relapse.

# STUDY PROTOCOL

# TRIAL DESIGN

Single arm, open label study with continuous monitoring of serious adverse events using the Bayes factor single arm design of Johnson and Cook (4). The small number of patients on treatment are insufficient to conduct a standard parallel group randomised, controlled trial. An economic analysis, informed by the results of this trial, will determine whether Eculizumab withdrawal, substituting treatment with a protocolised surveillance and treatment reintroduction strategy, is cost-effective.

# ELIGIBILITY CRITERIA

Patients with a diagnosis of aHUS (based on defined criteria – www.rarerenal.org) receiving Eculizumab to treat disease in native or transplanted kidneys. This is estimated to be 150 patients in the UK during the study period (110 prevalent patients and 40 incident patients). Genetic eligibility will be confirmed by the central trial team who will contact sites to confirm that they have a potentially eligible patient for site screening. Site eligibility must be assessed by a medically qualified doctor, and for those patients who enter into the trial, this assessment must be documented in the participant’s medical notes. A copy of both the anonymised genetic eligibility checklist AND the site eligibility checklist must be filed in the participant’s medical notes and sent to the NCTU as instructed on the forms. The eligibility checklist must also be completed in the MACRO database for those patients who enter into the trial. Only personnel formally delegated by the Principal Investigator to assess eligibility may perform this task.

## Inclusion Criteria

All patients must fulfil the following criteria in order to be eligible for the trial:

- Age ≥2+ years of age
- On Eculizumab treatment for at least 6 months
- In remission with no evidence of ongoing microangiopathic haemolytic anaemia (MAHA) activity at screening defined by:
- Platelet count > lower limit of normal as determined by local reference range
- Lactate Dehydrogenase (LDH) <x2 upper limit of normal as determined by local lab reference ranges
- Normal renal function or Chronic Kidney Disease (CKD) stages 1-3
- Absence of decline of renal function confirmed by review of available assessments of renal function for the preceding 6 months by the Chief Investigator and clinical members of the Trial Management Group (TMG)

The following criteria must be met by those only wishing to participate in the withdrawal component of the trial:

- Willing to attend for safety monitoring assessments
- Willing to travel only to countries that can supply Eculizumab (to be confirmed with co-ordinating centre prior to travel).
- Able to perform or parent/guardian to perform and record self-monitoring urinalysis
- Sexually active female patients must have a negative pregnancy test at screening and be using an effective contraception for the duration of the study as defined in table 1.

OR

- fulfil one of the following criteria:
- Be post-menopausal
- Have undergone surgical sterilisation

| **Highly effective methods of contraception** | **Failure Rate** |
| --- | --- |
| Implant | <1% |
| Injectable (combined hormone) | <1% |
| Intra-uterine device | <1% |
| Intrauterine system | <1% |
| Male Sterilisation | <1% |
| Injectable (single hormone) | <1% |
| Combined Oral | <1% |
| Progesterone Only | 1% |

**Table 1. Recommended Birth Control Methods**. (<http://www.chcuk.co.uk/pdf/2011-03-12_GCP_Considerations_Contraception_(CHCUK).pdf)>

## Exclusion Criteria

The following exclusion criteria is applicable to all patients wishing to participate in the trial:

- Severe non-renal disease manifestations at initial presentation with aHUS, which in the opinion of the Chief Investigator and/or the clinical members of the TMG makes the risk of treatment withdrawal unacceptable.
- Current or planned pregnancy within the study duration
- Unable to give informed consent or assent, or unable to obtain parent/guardian consent if under 16 years of age
- Current participation in another clinical trial (not including participation in ahus registries)
- Severe, uncontrolled hypertension (systolic blood pressure >160 mmHg) that is likely to induce at TMA.

The following exclusion criteria is applicable only to those wishing to participate in the withdrawal component of the trial:

- Loss of a previous transplant kidney to recurrent aHUS
- Transplant recipient with a pathogenic mutation in *C3*, *CFH* or *CFB*
- Haematuria rating of 3+

**NB: Enrolling a patient onto the trial who does not meet the inclusion/exclusion criteria is considered a protocol waiver and is in breach of Regulation 29 (SI 2004/1031) of the Medicines for Human Use (Clinical Trials) Regulations 2004. PROTOCOL WAIVERS ARE NOT PERMITTED.**

# TRIAL PROCEDURES

## Recruitment

### Patient Identification and Central Pre-Screening

Patients with a diagnosis of aHUS (based on defined criteria – www.rarerenal.org) receiving Eculizumab to treat disease in native or transplanted kidneys will be identified by the central clinical team at Newcastle as potentially eligible for the trial. A list of patients who fulfil these criteria is maintained by the National aHUS Service based within the NRCTC as part of the NHS England commissioned service. This information is available to the central clinical trial team and will allow identification of patients who potentially fulfil the eligibility criteria. The central clinical trial team will assess eligibility by review of the patient’s medical history available on the National aHUS service database. Those patients who meet the genetic eligibility criteria will be highlighted to sites.

It is estimated that there will be 150 patients in the UK who are on Eculizumab for the treatment of aHUS during the study period (110 prevalent patients and 40 incident patients). From our preliminary assessment of the current patient cohort (approximately 100 patients) 30% will not meet the inclusion/exclusion criteria, leaving a pool of approximately 60-70 prevalent patients eligible to participate in the study.

### Site Screening (day -28 to -14)

On receipt of the Central pre-screen eligibility checklist, the site can begin screening activities. This will include a review of the patients’ notes for their medical history and concomitant medications by the site research team. A physical examination and vital signs will be performed and routine safety laboratory tests will be reviewed to ensure that the patient fulfils all eligibility criteria (section 6.1 & 6.2) for entry into the study (please see section 7.4-Schedule of events, day -28 to day -14). Site screening activities may occur up to 4 weeks prior to Baseline (Day 0 +/- 2 days) and may occur on day -14 when the patient receives their final Eculizumab infusion. Any queries regarding patient eligibility should be considered first by the PI at site and further discussed with the CI or member of the central clinical trial team before the patient is formally consented.

Recruitment of patients who are non-English speakers will be reviewed on a case by case basis. If the site has access to an interpreter who can attend all monitoring visits, the central research team will explore the possibility of arranging to have the patient documentation translated into the required language.

Once patients have consented to take part in the trial, those female participants who are taking part in the withdrawal component and are sexually active will be required to have a pregnancy test as part of the screening process.

## Consent

Informed consent and assent will be sought by a member of the trial team who is suitably qualified, appropriately trained and delegated to do so. Potential participants, or their parents/legal guardian for a minor, will be given at least 24 hours after being given the Patient Information Sheet (PIS) to consider their participation in the study. As part of the consent and/or assent discussion, the requirement for sexually active females to use effective contraception must be discussed and confirmation of this discussion documented as a statement in the patients notes. Consent will be sought from the parents/legal guardian on behalf of patients under the age of 16. Assent will be taken from those patients under 16 years old.

The original signed Consent and assent forms will be retained in the Investigator Site File (ISF), with copies provided to the patient, a copy filed in the patients’ clinical notes and a copy sent by secure methods to NCTU. The copy sent to NCTU for monitoring purposes will be destroyed once reviewed. The site will allocate the participant their Unique Identification Number once they have consented to the study. Please note that this is different to the screening number that will be given to sites by the central trial team following genetic eligibility confirmation. The Unique ID number for withdrawal patients will be made up of the 2 digit site code and a 3 digit patient code starting with 001 for the first patient and continuing sequentially with each consenting patient. Those participants who are taking part in the Health Economics component only will have a unique ID number made up of the 2 digit site code and a 3 digit patient code starting with 101 for the first patient and continuing sequentially with each consenting patient.

### Final Eculizumab Infusion (Visit 1, Day -14)

Patients who consent to withdrawal will receive their last dose of Eculizumab at this visit (day -14). The patients will also receive their meningococcal prophylaxis as usual.

**Trial Assessments**

### Baseline Assessments & Data (Visit 2, Day 0 +/- 2 days)

Study day 0 will be the day that the patients would usually receive their next dose of Eculizumab based on standard dosing schedules (+/-2 days). The Eculizumab will not be administered however, the meningococcal prophylaxis will be continued for a further 2 weeks after day 0 to reduce risk of meningococcal infection. Patients will be trained at site to complete home urinalysis. This training must be documented in the patients notes.

At day 0 of the study (Visit 2), Eculizumab will not be administered. Patients will undergo the following assessments:

- Vital signs (temperature, pulse and Blood pressure)
- Height & weight
- Assessment of renal function (creatinine and estimated GFR).
- Urinalysis and urine protein/creatinine ratio
- Haemolysis markers including platelet count, haemoglobin, LDH
- Electrolyte Profile (U&Es)
- Liver Function (Bilirubin, ALT/AST, ALP, LDH, serum calcium, phosphate, albumin & total protein)
- Haptoglobin (if available) and blood film
- Concomitant Medication review
- Health-related quality of life questionnaires (EQ-5D-5L and SF-36)
- Health care utilisation questionnaire
- Biomarkers and complement activation sample – Sample to be taken and stored at site before transfer to Newcastle University for analysis (see 7.6).

### Study Visits (Visits 3-34)

**Clinical Assessments**

Patients will be assessed regularly for evidence of disease relapse for the 2 year duration of the study. The patients will attend a total of 34 visits* over the 2 year withdrawal follow-up period. Please see schedule of events (Section 7.4).

Study participants will be reviewed at the study site weekly (+/- 2 days) for the first month then alternate weeks (+/-2 days) until month 6, then monthly (+/- 7 days) thereafter until the end of the study period (month 24). During the COVID-19 pandemic and due to site staff availability, it is understood that patients may be seen outside of but as close to the visit window as possible.

At each study visit, patients will undergo the following assessments:

- Vital signs (temperature, pulse and Blood pressure)
- Renal function (creatinine and estimated GFR)
- Urinalysis
- Haemolysis markers including platelet count, haemoglobin and LDH
- Electrolyte Profile (U&Es)
- Liver function (Bilirubin, ALT/AST, ALP, LDH, albumin, total protein, serum calcium & phosphate)
- Concomitant Medication review
- Urinalysis Diary review
- Adverse Events

*Due to the covid-19 pandemic, patients may be unable to attend their scheduled follow up visits or may be attending a local hospital to have safety bloods taken. If the patients are unable to attend site due to self-isolation or underlying health issues; where possible, a remote, follow-up call will be carried out by a member of the local research team. During the remote follow-up call patients will be asked to report changes to their concomitant medications, any adverse events experienced since their previous follow-up and the results of their home urinalysis tests that will continue to be recorded in their urinalysis diary.

Paediatric participants must have their weight recorded at every visit for calculation of estimated GFR.

As haptoglobin is not a standard test that is performed at every NHS hospital, this is not a compulsory measure for those sites where it is not performed. Haptoglobin availability should be discussed with the central research team prior to starting recruitment at site.

Participants will provide the following at visits 2, 3, 4, 6, 8, 10, 16, 19, 22, 28 and 34.

- Biomarkers and complement activation samples

Participants will undergo the following assessments at study visits 2, 6, 10, 16, 22, 28 and 34.

- Haptoglobin (if available) and blood film
- Urine PCR

Participants will undergo the following assessments at study visits: 22 & 34:

- Physical Examination*

*Due to the COVID-19 pandemic and social distancing requirements and guidelines set out by individual hospital Trusts, patients may not be able to meet face to face with a clinician for their physical examination at the timepoints stated in the schedule of events table (section 7.4).

Following each patients visit the test results and assessment data collected should be entered into the MACO database in a timely manner and before the patient’s next scheduled visit.

### Self-monitored Urinalysis

Patients or carers will be trained to perform and understand the results of home urinalysis. Urinalysis will then be performed daily by the patient or carer for the first month and then three times per week for the remainder of the study period. The results will be recorded in a patient-held record and will be reviewed at each study visit (see Section 7.4. Schedule of Events). The haematuria ratings documented in the patient diary will be reviewed by the research nurse at each safety monitoring visit and any significant changes from baseline will be entered into MACRO at site. A new 2 week diary will be given to patients at each visit and the completed diaries will be retained by the site for monitoring purposes. During the COVID-19 pandemic, patients who are unable to attend site, those who are attending a local hospital for safety bloods or having bloods taken at home will be contacted where possible by a member of the local research team who will review their urinalysis diary over the telephone.

Patients or carers will report any significant change in urinalysis that is not related to menstruation to the study site using their own baseline to guide them in relation to the thresholds as detailed in table 2 below:

| Baseline | urinalysis result threshold  (not related to menstruation) |
| --- | --- |
| Neg/Trace | ++ on any occasion  OR  + on any two occasions 24 hours apart |
| + | +++ on any occasion  OR  ++ on any two occasions 24 hours apart |
| ++ | +++ on any occasion |

Table 2. Home Urinalysis result thresholds

If these criteria are met, patients will contact their treatment centre immediately to arrange assessment of disease activity (Renal function, haemoglobin, platelets, LDH, LFTs, haptoglobin and blood film, complement status) as outlined in the Schedule of Events Unscheduled visit column (See section 7.4).

### Change in Health Status

Patients participating in the study will also be advised to report any significant change in health status to the responsible site or local health care provider. Patients will be provided with a patient card to present to attending medical staff with details of the study, tests required and study centre and National aHUS Service contact details. Sites will send out the GP letter provided to notify the patients’ General Practitioner of their involvement in the study and informing them of the required action to be taken in the case of suspected relapse.

If there is a clinical suspicion of disease activity formal assessment will occur (renal function, haemoglobin, platelets, LDH, LFTs, haptoglobin, blood film, U&Es and complement status). At each study visit, patients will be asked about previously unreported adverse or serious adverse events that may have occurred since the last contact.

During the COVID-19 pandemic, patients who are unable to attend site, those who are attending a local hospital for safety bloods or having bloods taken at home will be contacted remotely where possible by a member of the local research team who will ask about previously unreported adverse or serious adverse events that may have occurred since the last contact.

## Disease Relapse

### Definition of disease relapse

A relapse in aHUS after withdrawal of Eculizumab will be diagnosed by:

1. Haematological relapse with a MAHA evidenced by the presence of the following criteria:

a. Platelet count of <150 x 109 /l (if normal at screening) or a fall in platelet count by >50% from screening

b. An increase in LDH by >50% above screening or haemoglobin < lower limit of normal for age and gender

OR

2. The presence of renal disease manifest by a decline in renal function (Acute Kidney Injury Network stage 1 AKI) confirmed on repeat testing after 6 hours) that is not explained by another pathology

OR

3. The presence of histological features of an active TMA on tissue biopsy performed as per local protocols for the investigation of renal disease.

Any other adverse events that could represent a relapse should be discussed with the Investigators and/or the aHUS National Service. There is a Consultant physician on call for the aHUS National Service at all times who should be informed by the responsible physician of any case where there is diagnostic uncertainty. A decision to restart will be made according to current Service procedures, requiring a consensus opinion from the consultant staff following review of the available clinical information.

### Management of a Relapse

Any patient with a suspected relapse should be reported to the PI at the local site and the aHUS National Service. This event would also need to be reported as an SAE following the procedure outlined in section 10.2 of this document.

When a relapse is diagnosed patients will restart Eculizumab treatment within 24 hours of presentation:

- Provided there is no evidence of an active infection that would be a contra-indication to treatment;
- At the recommended dose of 900mg weekly for the first 4 weeks then 1200mg every two weeks thereafter (or age adjusted dose);
- With monitoring of TMA activity (platelet count, LDH) as recommended by attending clinician until haematological remission is achieved. Haematological remission is defined as both:
  - Normalisation of platelet count (>150 x 109 /l)
  - Normalisation of serum LDH or within 50% of baseline level
- Maintenance of remission and effect of treatment will be monitored according to current recommended guidelines ([www.rarerenal.org](http://www.rarerenal.org/)). This consists of:
  - Laboratory tests to confirm complement blockade (CH50 and AP50) prior to 2^nd^ and 6^th^ dose of Eculizumab
  - Tests to confirm TMA remission (haemoglobin, platelet count, LDH, haptoglobin, urinalysis and urine protein creatinine ratio) monthly until the end of the study.

Patients who relapse and require re-introduction of Eculizumab treatment will remain on treatment in study under follow up for the full 2 years of the study. Hospital attendance for administration of treatment will constitute a study visit with reporting of adverse events. Home urinalysis will not be required after re-introduction of Eculizumab treatment.

Complement samples **should** be taken when a participant relapses in the UK. If the site does not have storage facilities, transportation to Newcastle will be arranged at no additional cost to the site. In such cases the site must contact the aHUS National Service on 0191 2820385 (9am to 5pm) or the Newcastle upon Tyne Hospitals switchboard (0191 2336161) requesting to speak to the clinician on call for the aHUS service to arrange this.

Due to the COVID-19 pandemic it is understood that it may not be possible for the biomarker and complement activation samples to be obtained and processed due to research staff availability. The Newcastle biobank will be closed due to government lockdown guidelines and will not be able to accept any research samples during this time. Sites will be informed when the biobank will be open to receive samples.

Patients will consent to travel to only those countries where Eculizumab is available and this must be confirmed with their clinician prior to booking any travel. The site should inform the National aHUS service if a patient plans to travel outside of the country. The destination and dates of travel should be disclosed so that a potential relapse event can be dealt with as rapidly as possible. Patients should be reminded that they will need to continue to complete the urinalysis diary while on holiday as this is an indicator of disease activity. Patients must also be reminded to carry their Patient card at all times and to present this card to the clinician treating them should they visit a hospital while outside of the country. If patients relapse while they are travelling outside of the country, the National aHUS Service will make arrangements with the destination country to access and fund Eculizumab if required, with arrangements from the commissioning authority, NHS England. Sponsor insurance does not cover a patient’s hospital treatment and other associated costs while travelling outside of the country and participants are reminded in the Patient Information Sheet that they must arrange their own travel insurance to cover these costs and inform their insurance provider of their participation in the Clinical Trial.

## Schedule of Events for withdrawal participants

Table 2. Schedule of events for withdrawal participants, day -42 to 672

## Schedule of Events for Health Economics participants

Table . Schedule of events for Health Economics only participants, day -42 to 672

## Trial Withdrawal Criteria

Participants have the right to withdraw from the trial at any time without having to give a reason. Investigators at sites should try to ascertain the reason for withdrawal and document this reason within the eCRF and participant’s medical notes.

The Investigator may discontinue a participant from the trial at any time if the Investigator considers it necessary for any reason including:

- Participant withdrawal of consent
- Significant protocol deviation or non-compliance
- Investigator’s discretion that it is in the best interest of the participant to withdraw from the study not relating to re-starting Eculizumab treatment
- An adverse event that renders the participant unable to continue in the trial
- Termination of the clinical trial by the sponsor

Participants who withdraw from the trial will not be replaced.

## End of Trial

The last visit will be defined as the last patient attending their visit 34 on day 672 (+/-7 days).

The final treatment outcome data will be collected when the final patient attends for their visit 34. At this point the end of study notification will be submitted to the Research Ethics Committee (REC). The data analysis and final report will be completed within a year of this study notification.

The trial may be ended prematurely on the recommendation of the DMC & TSC if SAE’s or interim analysis indicates that this is required. The criteria of such decisions will be decided by the DMC & TSC as specified in the respective charter and SAP.

# WITHDRAWN MEDICATION

## Name and Description of withdrawn Medication

## Eculizumab

Eculizumab is a humanised monoclonal antibody directed against complement component 5. It is licenced for treatment of aHUS and paroxysmal nocturnal haemoglobinuria. For aHUS, the licence is for continuous treatment once initiated.

## Access to Eculizumab

Eculizumab is available to all sites in the UK within 24 hours to permit rapid treatment of active disease. This will be organized by the local site or coordinating centre. If patients are travelling overseas, we will make arrangements with the destination country to access Eculizumab if required, with arrangements from the UK commissioning authority.

## Relapse Information

The withdrawal of Eculizumab treatment could lead to a relapse of aHUS and complications associated with both relapse and failure to re-initiate treatment within 24 hours. Relapse should be considered if:

1. The participant presents with symptoms or signs that would be consistent with the development of a TMA including neurological symptoms signs or of renal disease (macroscopic haematuria, fluid retention, fatigue)
2. The participant presents with any unexpected illness
3. Evidence of thrombocytopenia, microangiopathic haemolytic anaemia or deterioration in renal function at a study visit
4. Increased haematuria detected by urinalysis

This should lead to a full assessment for the TMA which will be identified by the presence of:

1. Thrombocytopenia
2. Microangiopathic haemolysis (fall in haemoglobin, rise in LDH, red cell fragmentation)
3. Deterioration in estimated GFR

## Dosage Schedule

If re-introduction of Eculizumab is required, this should follow the standard dosing regime.

## Concomitant Medications

**Meningococcal prophylaxis**

Participants will continue with prophylactic antibiotics for 4 weeks after the last dose of Eculizumab prior to withdrawal to reduce risk of meningococcal infection.

If re-introduction of Eculizumab is required the meningococcal prophylaxis will also be re-started as per the standard protocols.

# PHARMACOVIGILANCE

## Definitions

| Term | Definition |
| --- | --- |
| Adverse Event (AE) | Any untoward medical occurrence in a participant to whom a medicinal product has been withdrawn including occurrences which are not necessarily caused by or related to withdrawal of that product. |
| Adverse Reaction (AR) | An untoward or unintended response in a participant to an investigational medicinal product which is related to any dose administered to that participant.  The phrase “response to an investigational medicinal product” means that a causal relationship between a trial medication and an AE is at least a reasonable possibility i.e. the relationship cannot be ruled out.  All cases judged as having a reasonable suspected causal relationship to the medication qualify as adverse reactions. |
| Reference Safety Information (RSI) | The RSI is a list of medical terms detailing the ARs that are expected for a medicinal product and must be referred to when assessing a SAR for expectedness. |
| Serious Adverse Event (SAE) | A serious adverse event is any untoward medical occurrence that:   - Results in death - Is life-threatening* - Requires inpatient hospitalisation or prolongation of existing hospitalisation - Results in persistent or significant disability/incapacity - Consists of a congenital anomaly or birth defect - Other important medical events that jeopardise the participant or require intervention to prevent one of the above consequences   * - life-threatening refers to an event in which the participant was at immediate risk of death at the time of the event; it does not refer to an event which hypothetically might have caused death if it were more severe. |
| Serious Adverse Reaction (SAR) | An adverse event that is both serious and, in the opinion of the reporting Investigator, believed with reasonable probability to be due to the trial treatment, based upon the information provided. |
| Suspected Unexpected Serious Adverse Reaction (SUSAR) | A serious adverse reaction, the nature and severity of which is not consistent with the approved Reference Safety Information. |

## Recording and Reporting AEs and SAEs

All AEs occurring from the point of withdrawal (day 0) to end of study participation must be recorded in the study database eCRF as well as the participant’s medical notes.

SAEs occurring from the point of withdrawal (day 0) must be reported to NCTU within 24 hours of the site becoming aware of the event. Any SAE that comes to the attention of the site team must be recorded and reported up until the point of study closure.

For each SAE the following information will be collected:

- Full details in medical terms and case description
- Event duration (start and end dates, if applicable)
- Action taken
- Outcome
- Seriousness criteria
- Causality in the opinion of the investigator
- Whether the event is considered expected or unexpected in accordance with the approved Reference Safety Information if a causal relationship is suspected

Any change of condition or other follow-up information should be submitted to the NCTU as soon as it is available or at least within 24 hours of the information becoming available. Events will be followed up until the event has resolved or a final outcome has been reached.

## Recording and Reporting SUSARs

SUSARs are reportable only for those patients who are withdrawn from Eculizumab and are subsequently put back on to drug during their participation in the trial. All SUSARs occurring from the first day that a patient has their Eculizumab treatment restarted until the last day of their follow up period (24 months post initial termination of Eculizumab) must be reported to the Medicines and Healthcare products Regulatory Agency (MHRA) and REC. The NCTU will perform this reporting.

The assessment of expectedness will be performed by the PI at site against the approved Reference Safety Information (RSI) for the trial (Section 4.8 of the Soliris SmPC).

Fatal and life-threatening SUSARS must be reported to the MHRA no later than 7 calendar days after the Sponsor has first knowledge of the event. Any relevant follow-up information must be sought and reported within a further 8 calendar days.

Non-fatal or non life-threatening SUSARs must be reported to the MHRA no later than 15 calendar days after the Sponsor has first knowledge of the event. Any relevant follow-up information should be sought and reported as soon as possible after the initial report.

As soon as a site suspects that a SAR may be a SUSAR they must contact the CI, sponsor representative and the trial manager immediately. The reporting timeframe starts at day 0 when the NCTU is in receipt of a minimum set of information:

- Sponsor trial reference and trial name (sponsor reference)
- EudraCT number
- Patient trial number and date of birth
- Date of notification of the event
- Medical description of the event
- Date and time of the onset of the event (including event end date if applicable)
- Causality assessment
- Seriousness of the event, particularly if life threatening or fatal
- An identifiable reporter (e.g., Principal Investigator)

This information must be provided on the SAE Form provided to site. The site is expected to fully cooperate with the NCTU in order that a full and detailed report can be submitted to the MHRA and REC within the required timelines.

PIs will be informed of all SUSARs by the C.I or member of the central clinical trial team.

## Responsibilities

***Principal Investigator***

- *Checking for AEs and ARs when participants attend for treatment or follow-up*
- *Using medical judgement in assigning seriousness and causality and providing an opinion on expectedness of events using the Reference Safety Information approved for the trial.*
- *Ensuring that all SAEs and SARs, including SUSARs, are recorded and reported to the Sponsor within 24 hours of becoming aware of the event and provide further follow-up information as soon as available.*
- *Ensuring that AEs and ARs are recorded and reported to the Sponsor in line with the requirements of the protocol.*

***Chief Investigator***

- *Clinical oversight of the safety of trial participants, including an ongoing review of the risk/benefit.*
- *Using medical judgement in assigning seriousness, causality and expectedness of SAEs where it has not been possible to obtain local medical assessment.*
- *Using medical judgement in assigning expectedness to SARs.*
- *Immediate review of all SUSARs.*
- *Review of specific SAEs and SARs in accordance with the trial risk assessment and protocol.*

***Sponsor***

- *Assessment of expectedness of any SUSARs*
- *Expedited reporting of SUSARs to the CA and REC within required timelines*
- *Notification of all investigator sites of any SUSAR that occurs*

***TSC/DMC***

- *Review of safety data collected to date to identify any trends*

## Notification of Deaths

All deaths will be reported as SAE’s irrespective of the cause of death and reported to the NCTU following the process outlined in section 10.2. All deaths will be reported to the trial oversight committee.

## Pregnancy Reporting

In the event of a study participant becoming pregnant on study the site must notify NCTU, the Chief Investigator and the sponsor representative within 24 hours of becoming aware of the pregnancy.

Site must approach the study participant to obtain consent to follow the pregnancy to completion.

## Reporting Urgent Safety Measures

An Urgent Safety Measure (USM) is an action that the Sponsor or an Investigator may take in order to protect the subjects of a trial against any immediate hazard to their health or safety. Upon implementation of an USM by an Investigator, the Sponsor/CI/NCTU must be notified immediately and details of the USM given. The Sponsor/CI/NCTU must inform the MHRA and the NHS REC within 3 days of the USM taking place in accordance with the Sponsor’s/NCTU’s standard operating procedures.

## Development Safety Update Reports

A Development Safety Update Report (DSUR) must be submitted to the MHRA and NHS REC once a year on the anniversary of the Development International Birth Date (DIBD). The Trial Manager must ensure that the report is submitted within 60 days of the end of the reporting period. The Trial Management Group must input into the compilation of the DSUR and the CI must review and authorise the final report before it is ready for submission. The DSUR should also be reviewed by the NCTU QA Manager and sponsor Representative prior to submission via the Common European Submission Portal (CESP) system.

# STATISTICAL CONSIDERATIONS

**Analysis Population**

All consented patients who withdraw from Eculizumab will be included in the statistical analyses.

## Statistical Analyses

A detailed Statistical Analysis Plan (SAP) will be finalised giving full detail of the planned analyses in advance of these being undertaken. These analyses are summarised below.

### Analysis of the Primary Outcome Measure

This is a single arm, open label trial with the primary endpoint being a binary response (the presence/absence of a primary outcome event (TMA related SAEs as defined in section 3.3) within the follow-up period). We will compare the rate of serious events following the withdrawal of medication in the study to that expected under standard care. A maximum of 30 patients will be recruited; this is judged to be a feasible level of recruitment in a reasonable timeframe given the rare nature of the disease. There is no allowance for loss to follow-up as this patient group is already subject to a high degree of clinical follow-up and death is defined as one of the serious events under consideration.

The Bayes factor single arm binary model (4) will be used to monitor the trial. Based on historical data, the event rate for the standard of care is 0.06, and we expect that withdrawal of the treatment would give a response rate of 0.12. This choice of an acceptable event rate has been informed in discussion with patients. Using this Bayesian hypothesis test-based design, we assume the serious event rate is 0.06 under the null hypothesis, and the event rate is 0.12 under the alternative hypothesis.

We assume that the sample distribution of number of responses follows a binomial distribution, and use an inverse moment prior for response under the alternative hypothesis.

**Stopping Rules**

A minimum of 5 patients will be enrolled before applying the stopping rules, and the cohort size for monitoring the trial is 5 patients. The DMC can request earlier review if adverse events are reported before this point.

We implement two stopping rules during the trial:

(1) We will stop the trial for superiority (there being fewer serious events on the intervention than would be expected under standard of care) if the posterior probability of the alternative hypothesis is less than 0.05, i.e. Pr(H1|Data) < 0.05;

(2) We will stop the trial for inferiority if the posterior probability of the alternative hypothesis is greater than 0.80, i.e., Pr(H1|Data) > 0.80.

**Operating Characteristics**

The operating characteristics of the design were produced using the M. D. Anderson Cancer Center Department of Biostatistics software BayesFactorBinary, version 1.0 (https://biostatistics.mdanderson.org/SoftwareDownload/ProductSupportFiles/BayesFactorBinary/UsersGuide_Bay<https://biostatistics.mdanderson.org/SoftwareDownload/ProductSupportFiles/BayesFactorBinary/UsersGuide_BayesFactorBinary.pdf>).

| Scenario | True rate of serious events | Probability of Stopping for Inferiority | Probability of Stopping for Superiority | Average number of patients treated (Percentiles: 10%, 25%, 50%, 75%, 90%) |
| --- | --- | --- | --- | --- |
| 1 | 0.06 | 0.096 | 0 | 28.44 (30, 30, 30, 30, 30 ) |
| 2 | 0.12 | 0.443 | 0 | 23.48 (5, 15, 30, 30, 30 ) |
| 3 | 0.18 | 0.753 | 0 | 17.76 (5, 10, 15, 30, 30 ) |
| 4 | 0.24 | 0.928 | 0 | 13.72 (5, 5, 15, 15, 30 ) |
| 5 | 0.30 | 0.982 | 0 | 10.52 (5, 5, 10, 15, 20 ) |

For example, if the true rate of serious events is 0.06 (Scenario 1, the null hypothesis), the trial will stop with probabilities of 0.096 and 0 in favour of the alternative and null hypotheses, respectively. The average number of patients (10%, 90% percentiles) treated is 28.44 (30, 30). If the true serious event rate is 0.12 (Scenario 2, the alternative hypothesis), the trial will stop with probabilities of 0.443 and 0 in favour of the alternative and null hypotheses, respectively. The average number of patients (10%, 90%) treated is 23.48 (5, 30).

**Stopping Boundaries**

The stopping boundaries of the design were produced using the M. D. Anderson Cancer Center Department of Biostatistics software **BayesFactorBinary**, version 1.0.

| Number of patients (in complete cohorts of 5) | **Stop** the trial for Superiority if there are this many **Events** (inclusive) | **Continue** the trial if there are this many **Events** (inclusive) | **Stop** the trial for Inferiority if there are this many **Events** (inclusive) |
| --- | --- | --- | --- |
| 5 | Never stop for superiority with this many patients | 0-1 | 2-5 |
| 10 or 15 | Never stop for superiority with this many patients | 0-2 | 3-15 |
| 20 | Never stop for superiority with this many patients | 0-3 | 4-20 |
| 25 or 30 | Never stop for superiority with this many patients | 0-4 (The trial always stops at 30 patients, which is the maximum) | 5-30 |

We have taken the conservative approach of stopping the study on the grounds of inferiority should there be 2 serious events in the first cohort of 5 patients. Subsequent to this, the study would stop should there be 3 or more serious events observed in the first 15 patients, 4 or more in the first 20 patients, and 5 or more in the whole study population during follow-up.

Therefore, we are well placed to quickly respond to any negative safety signal from the emerging data.

These numbers have been obtained using 1000 repetitions in the software simulation. These calculations have also been repeated using different numbers of repetitions; in these cases the resulting stopping boundaries are unchanged from those quoted above while the probability of stopping for inferiority and the average number of patients treated are marginally changed.

It is acknowledged that there may well be differing risk of relapse according to disease aetiology however, the available numbers do not allow for risk strata to be monitored separately. The Data Monitoring Committee (DMC) will consider the issue of risk based on disease aetiology as part of their remit.

In addition to this ongoing analysis, at the end of the study, the primary outcome data will also be reported descriptively, together with the number of patients recruited. Descriptive statistics reported will be selected from mean, median, standard deviation, range and inter-quartile range as appropriate to the specific outcome measure. For proportion outcomes, the number of patients recording the event will be reported together with this value.

Due to small the sample size, no comparative statistical methods will be applied. There will be no imputation of any missing data and analysis will take the form of a complete case analysis.

### Analysis of Secondary Outcome Measures

In line with the primary outcome data, at the end of the study, the secondary outcome data will also be reported descriptively, together with the number of patients recruited. The same descriptive statistics and restrictions to the analysis will be considered as for the primary outcome.

# 7. DATA HANDLING

## Data Collection Tools and Source Document Identification

All the data required will be recorded in clinical notes as part of routine clinical procedures. Data for individual patients will be transferred by each PI or his/her delegated nominee to the eCRF (the secure, validated clinical data management system, MACRO) as soon as possible following each procedure (see 7.2). Similarly, data will be entered into the eCRF following any non-scheduled visits. Patients will record results of urinalysis in a patient-held diary which will be reviewed, copied and data entered into the eCRF by study staff. Patient identification on the eCRF will be via a unique study identifier number assigned to the patient at site once the patient has consented. A record linking the patient’s name to the unique study identifier number will be held only in a locked room at the study site, and is the responsibility of the PI. As such, patients cannot be identified from eCRFs. The CI, or nominated designee, will continually monitor completeness and quality of data recording in CRFs and will correspond regularly with site PIs (or their delegated assistants) to capture any missing data where possible, and ensure continuous high-quality data.

The CDMS (Elsevier’s MACRO) used for this trial is fully compliant with all regulatory frameworks for research of this nature. It uses a secure web-based interface for data entry; no data are stored on computers at site. MACRO users are assigned role based permissions specific to their site and role. The system has an inbuilt back-up facility, through Elsevier’s hosting partner Rackspace’s secure premises in London, and is managed and supported by the Rackspace team.

## Data Handling and Record Keeping

All of the data collected will be kept strictly confidential and will be managed in accordance with the General Data Protection Regulation (GDPR). Identifiable data will be stored in a separate, limited-access database. Paper copies of study-related documentation will be annotated, signed and dated, and filed in the medical notes. The overall quality and retention of study data is the responsibility of the Chief Investigator. All study data will be retained in accordance with the latest Directive on GCP (2005/28/EC) and local policy.

## Access to Data

Clinical information will not be released without the written permission of the participant, except as necessary for monitoring and auditing by the Sponsor, its designee, Regulatory Authorities, the Data Monitoring Committee (DMC) or the REC. Secure, anonymised, electronic data will be released to the Study Statistician and Health Economist for analysis. The PI and study site staff may not disclose or use for any purpose other than performance of the study, any data, record, or other unpublished, confidential information to which they have access, in order to carry out the study. Prior written agreement from the Sponsor or its designee must be obtained for the disclosure of any confidential information to other parties.

## Archiving

Archiving of trial data transferred by eCRFs will be carried out according to NCTU and Sponsor SOPs. Routine clinical notes will be archived according to NHS Trust regulations at each site. All data will be retained for 5 years.

# MONITORING, AUDIT & INSPECTION

The trial will be managed through the Newcastle Clinical Trials Unit. The study will be co-ordinated by a Trial Management Group (TMG) that will include those individuals responsible for the day-to-day management of the trial. The TMG will monitor all aspects of the conduct and progress of the study, ensure that the protocol is adhered to and take appropriate action to safeguard participants and the quality of the study itself. TMG meetings will occur at least monthly and include a teleconference link to other trial sites, where required. Progress will be monitored proactively according to timelines and any issues addressed. The TMG will liaise with the Trial Steering Committee (TSC), providing updates on trial progress and highlighting any issues arising.

The Principal Investigators will be responsible for highlighting day-to-day study conduct at each site. The NCTU will provide day-to-day support for the sites and training, via Investigator meetings, site initiation visits and routine monitoring visits.

Quality control will be maintained through adherence to NCTU SOPS, Newcastle Joint Research Office SOPs, study protocol, GCP principles, research governance and clinical trial regulations.

An independent Data Monitoring Committee (DMC) will be established to oversee treatment outcomes during the study. During the trial, interim analyses of baseline, follow-up data and any other analyses requested by the committee, will be supplied, in strict confidence, to the DMC chair. The DMC will include an independent statistician. This committee will monitor efficacy, safety and clinical outcomes. At the first meeting, the DMC will agree on its charter of operation, and discuss and advise on the inclusion of an interim analysis and possible adoption of a formal stopping rule for efficacy or safety.

A Trial Steering Committee (TSC) will be established to provide overall –independent oversight of the trial, and will oversee trial conduct and progress. The Chair will be an independent consultant, and the committee will include one lay member. Members of the Trial Management Group (TMG) will attend these meetings. The TSC terms of reference and members’ names and contact details will be published ahead of its first meeting. At the first meeting, the TSC will agree on its charter of operation The committee will meet at least annually for the duration of the trial.

Clinical management of participants will remain subject to individual sites internal audit procedures. The Newcastle CTU will carry out monitoring to ensure appropriate study conduct and data collection. Electronic data will be stored on secure, password-protected computers. NCTU staff will use a combination of central review and site monitoring visits to ensure the study is conducted in accordance with GCP. The trial manager will conduct the site monitoring and will require access to the patients’ clinical notes and consent forms.

The following will be monitored:

- Presence of completed original consent forms in the Investigator Site File (ISF) and copies in patients’ notes.
- Existence of patients, by comparison of original consent forms with patient identification (enrolment) list.
- Reported serious adverse events, by verification against patient notes (source data verification).
- Presence of essential documents in the ISF and study files.
- Primary endpoint data for a percentage of study participants, by source data verification.
- Applications for study authorisations and submissions of progress/safety reports, for accuracy and completeness, prior to submission.
- Eligibility data for a percentage of study participants, by source data verification.

All monitoring findings will be reported and followed up with the appropriate persons in a timely manner. The study may be subject to inspection and audit by The Newcastle upon Tyne Hospitals NHS Foundation Trust under its remit as Sponsor, and other regulatory bodies, to ensure adherence to GCP. The investigators and institutions will permit study-related monitoring, audits, REC review and regulatory inspection(s), providing direct access to source data and documents relating to the study.

# ETHICAL AND REGULATORY CONSIDERATIONS

## Research Ethics Committee Review and Reports

The NCTU will obtain a favourable ethical opinion from an NHS Research Ethics Committee (REC) prior to the start of the trial. All parties will conduct the trial in accordance with this ethical opinion.

The NCTU will notify the REC of all required substantial amendments to the trial that result in a change to trial documentation (e.g. protocol or patient information sheet). Substantial amendments that require a REC favourable opinion will not be implemented until this REC favourable opinion is obtained. The NCTU will notify the REC of any serious breaches of GCP or the protocol, urgent safety measures or SUSARs that occur during the trial.

An annual progress report will be submitted each year to the REC by NCTU until the end of the trial. This report will be submitted within 30 days of the anniversary date on which the original favourable ethical opinion was granted.

The NCTU will notify the REC of the early termination or end of trial in accordance with the required timelines.

## Peer Review

The trial protocol has been discussed with experts in the management of aHUS in the UK and in other countries who have experience of withdrawing Eculizumab. The internal team includes clinicians with experience in the treatment of aHUS and conducting multicentre clinical trials in aHUS and other diseases. The team has a diverse background including co-applicants with expertise in trial design and analysis, qualitative research, social sciences and health economics. The funding process included external peer review. The applicants responded to the peer review reports and the funding was awarded.

The protocol described is considered sufficient to detect relapse and similar protocols are under development in other countries with the expectation that withdrawal can be undertaken safely.

## Public and Patient Involvement

Patients and the patient group (aHUSUK) have been involved in trial design and dissemination of information about the trial and collection of patient feedback. The acceptable level of monitoring was discussed with patients and has been altered to reflect their suggestions. For example, we will maximize flexibility for study visits and, in case of problems during the withdrawal phase, we will provide written patient information and access to the aHUS National Service.

## Regulatory Compliance

The trial will be conducted in accordance with the Medicines for Human Use (Clinical Trials) Regulations 2004 and subsequent amendments. All parties must abide by these regulations and the ICH GCP guidelines.

The NCTU will obtain a Clinical Trial Authorisation (CTA) from the MHRA prior to the start of the trial and will notify the MHRA of any substantial amendments that require review by the competent authority. These substantial amendments will not be implemented until the MHRA have issued an acceptance of the amendment.

The NCTU will notify the MHRA of any serious breaches of GCP or the protocol, urgent safety measures or SUSARs that occur during the trial.

The Development Safety Update Report will be submitted each year to the MHRA by the NCTU until the end of the trial.

The NCTU will notify the MHRA of the early termination or end of trial in accordance with the required timelines.

## Protocol Compliance

Protocol deviations, non-compliances and breaches are departures from the approved protocol. Unintentional protocol deviations will be documented and reported to the CI and sponsor. Where necessary, Corrective and Preventative Actions (CAPA) will be implemented. These will also be documented and reported to the CI and sponsor. Deviations found to frequently recur at a site are not acceptable and could be classified as a serious breach.

## Notification of Serious Breaches to GCP and/or the Protocol

A serious breach is a breach that is likely to effect to a significant degree –

- the safety or physical or mental integrity of the subjects of the trial; or
- the scientific value of the trial

The sponsor must be notified immediately of any incident that may be classified as a serious breach. The NCTU will notify the MHRA and the NHS REC within the required timelines in accordance with the NCTU SOP.

## Data Protection and Patient Confidentiality

Personal data will be regarded as strictly confidential. All data retained at site and sent electronically to the main co-ordinating centre will contain Study ID and initials only. The secure password-protected eCRF database (MACRO) also requires initials and date of birth. This is essential for participant identification and verification. This information is also required for the fax-to-email SAE reporting system.

All personnel with access to trial data are qualified and trained in, and will comply with ICH GCP. Justification for all such electronic transmissions will be approved by Sponsor and covered in the Caldicott applications made locally at each site.

A Participant Identification List will be the only document retained within the ISF, which contains full details of hospital number, patient name, and study ID.

The study will comply with the Data Protection Act, 1998. All study records and Investigator Site Files will be kept at site in a locked filing cabinet with restricted access to those who are named on the SETS aHUS delegation log at each Site.

## Indemnity

The NHS Trust has liability for clinical negligence that harms individuals toward whom they have a duty of care. NHS Indemnity covers NHS staff and medical academic staff with honorary contracts conducting the trial for potential liability with respect to negligent harm arising from the conduct of the study. The Newcastle upon Tyne Hospitals NHS Foundation Trust is the Sponsor and through the Sponsor, NHS indemnity is provided with respect to potential liability and negligent harm arising from study management. Indemnity, with respect to potential liability arising from negligent harm related to study design, is provided by NHS schemes for those protocol authors who have their substantive contracts of employment with the NHS, and by Newcastle University Insurance schemes for those protocol authors who have their substantive contract of employment with Newcastle University. This is a non-commercial study and there are no arrangements for non-negligent compensation.

## Amendments

It is the responsibility of the Research Sponsor to determine if an amendment is substantial or not and study procedures must not be changed without the mutual agreement of the CI, Sponsor and the Trial Management Group.

Substantial amendments will be submitted to the REC and/or MHRA (as appropriate) and will not be implemented until this approval is in place. It is the responsibility of the NCTU to submit substantial amendments.

Non-substantial amendments will be submitted to the Health Research Authority (HRA) and will not be implemented until authorisation is received.

Substantial amendments and those minor amendments which may impact sites will be submitted to the relevant NHS R&D Departments for notification to determine if the amendment affects the NHS permission for that site. Amendment documentation will provide to sites by the NCTU.

## Post-Trial Care

After the Trial, patients will be followed up according to the routine review procedures within the clinic. No additional follow-up is required in relation to the Trial. Those patients who withdrew from Eculizumab may remain withdrawn or return to treatment depending on their preference and clinical opinion.

## Access to the Final Trial Dataset

Until publication of the Trial results, access to the full dataset will be limited to the Project Management Group and to authors of the publication. If not co-authors, the leads for clinical sites will be given access to the full dataset from all participating Centres (anonymised) that is included in the Trial publication.

# Trial Steering Committee

Independent members

Professor Jeremy Hughes (Chair)

Dr Paul Tappenden

Professor Yvonne Birks

Vera Mackersie (patient representative

Dr Paul Warwicker

# Data Monitoring Committee

Independent members

Professor Phil Kalra

Dr Atif Awan

Dr Daniel Meddings (replaced by Dr Thomas Homberg)

# Patient and Public Involvement and Engagement

The trial design was designed and developed in collaboration with the aHUSUK – a charity that provides a voice for Atypical Haemolytic Uraemic Syndrome (aHUS) patients. At an aHUS Patient and Family Conference, the Chief Investigator presented the proposed trial. A focus group was held to specifically discuss the acceptable rate of withdrawal-related events, with the proposal that this should be set at two-fold the rate of treatment-related adverse events (set of 0.06 based on the first 100 patients treated by the aHUS National Service).

Subsequent patient feedback focused the design of the study around patient safety. This included ensuring that patients had immediate access to specialist medical care (and drug if necessary) through the aHUS National Service. A patient representative volunteered to be a co-applicant and coordinated further PPI activity throughout the trial. PPIE activity was costed in line with INVOLVE guidance. Patients were represented on the trial steering committee, providing a channel to inform patients on progress of the study. Provision for this was included in the trial costs. Patient and carers were be involved in the development of patient facing information resources to ensure that all study documentation provides the information required by patients in a suitable format.

# Supplementary table 1 Patient characteristics in the eculizumab withdrawal study

| **Patient ID** | **Age at withdrawal (years)** | **Complement regulatory defect** | **Classification** | **Duration of eculizumab treatment (months)** | **Outcome** |
| --- | --- | --- | --- | --- | --- |
| 1 | 2 | No | - | 18 | No relapse |
| 2 | 3 | No | - | 20 | No relapse |
| 3 | 8 | No | - | 6 | No relapse |
| 4 | 31 | *CD46* c.632delG (p.Gly211Valfs*22) | Pathogenic | 35 | No relapse |
| 5 | 4 | No | - | 6 | No relapse |
| 6 | 26 | *CFH* homozygousc.2836dupT (p.Ser946fs) | Pathogenic | 16 | No relapse |
| 7 | 35 | *CFH* c.3570T>G (p.Tyr1190*) | Pathogenic | 18 | Relapse |
| 8 | 37 | *CD46* homozygous c.97+2_97+12del | Pathogenic | 22 | Relapse^±^ |
| 9 | 31 | No | - | 32 | No relapse |
| 10 | 12 | Anti-FH autoantibodies (32000^#^) | ­- | 46 | No relapse |
| 11 | 8 | Anti-FH autoantibodies (1443^#^) | - | 24 | No relapse |
| 12 | 2 | No | - | 23 | No relapse |
| 13 | 14 | Anti-FH autoantibodies (9239^#^) | Pathogenic | 12 | No relapse |
| 14 | 3 | No | - | 20 | No relapse |
| 15 | 20 | Anti-FH autoantibodies (>4000^#^) | - | 15 | No relapse |
| 16 | 9 | *CFH* c.3643C>G (p.Arg1215Gly) | Pathogenic | 25 | Relapse |
| 17 | 39 | No | - | 20 | No relapse |
| 18 | 9 | *C3* homozygous c.3325C>G (p.Leu1109Val) | Pathogenic | 23 | No relapse |
| 19 | 6 | *C3* c.3470T>C (p.Ile1157Thr) | Pathogenic | 21 | Relapse |
| 20 | 25 | No | - | 21 | No relapse |
| 21 | 8 | *CD46* c.808T>C (p.Cys270Arg) | Pathogenic | 25 | No relapse |
| 22 | 2 | *CFH* c.2500A>C (p.Lys834Gln) | VUS | 21 | No relapse |
| 23 | 6 | C3 c.1618G>T (p.Ala540Ser) | VUS | 30 | No relapse |
| 24 | 11 | *CD46* c.286+2T>G | LP | 61 | No relapse |
| 25 | 43 | No | - | 44 | No relapse |
| 26 | 6 | Anti-FH autoantibodies (97^#¥^) | - | 15 | No relapse |
| 27 | 13 | *CD46* c.97+2_97+12del | Pathogenic | 56 | No relapse |
| 28 | 59 | No | - | 51 | No relapse |

^#^ Antibody titres closest to the point of treatment withdrawal, normal range <100. All patients with autoantibodies were homozygotic for a deletion at *CFHR1/3* locus

^±^ Met primary outcome

^¥^Peak titre 917

LP Likely pathogenic, VUS Variant of Uncertain Significance

# Supplementary table 2. Summary of haematology results

|  | Visit 2 (baseline) (n=27) | | | Visit 3 (7 days) (n=25) | | | Visit 4 (14 days)(n=28) | | |
| --- | --- | --- | --- | --- | --- | --- | --- | --- | --- |
| **Full blood count** | Mean  (SD) | Median (IQR) | Range | Mean  (SD) | Median (IQR) | Range | Mean  (SD) | Median (IQR) | Range |
| Haemoglobin (g/L) | 127.3  (15.3) | 127  (115-140) | 96-155 | 128.6  (12.5) | 127  (118-139) | 106-150 | 126.1  (14.9) | 129.5  (117.5-136) | 93-159 |
| Haematocrit (L/L) | 0.38  (0.04) | 0.38  (0.34-0.41) | 0.30-0.46 | 0.38  (0.04) | 0.37  (0.36-0.41) | 0.33-0.44 | 0.37  (0.04) | 0.38  (0.34-0.4) | 0.30-0.46 |
| RBC (10^12^/l) | 4.6^a^  (0.5) | 4.6  (4.3-4.9) | 3.5-5.5 | 4.7a  (0.5) | 4.6  (4.4-4.8) | 3.8-6.1 | 4.6a  (0.4) | 4.6  (4.2-4.8) | 3.87-5.62 |
| MCV (fl) | 82.4  (5.1) | 81.8  (79-87) | 72-92 | 82.1  (5.0) | 81.3  (78.4-86) | 72-90 | 81.9  (5.2) | 82  (78.1-86.4) | 70-90.5 |
| MCH (pg) | 27.8^a^  (1.9) | 27.9  (26.2-29.4) | 23.3-31 | 27.6a  (2.0) | 27.3  (26.3-29.3) | 22.9-31.4 | 27.7a  (2.0) | 27.1  (26.5-29.2) | 23.3-31 |
| Platelets (10^9^/l) | 282.7  (86.6) | 267  (228-324) | 184-533 | 308.4a  (113.4) | 286  (252-332) | 181-744 | 300.5  (117.0) | 287.5  (217.5-336) | 179-744 |
| White blood cells (10^9^/l) | 7.3  (2.5) | 6.5  (5.6-9.1) | 4.5-14.4 | 7.3  (2.7) | 6.6  (5.3-8.6) | 3.4-13.6 | 7.3  (2.3) | 7.2  (5.4-8.85) | 3.5-12 |
| Neutrophils (10^9^/l) | 3.8  (2.0) | 3.5  (2.4-4.5) | 1.3-9.9 | 3.7  (2.0) | 3.3  (2.4-4.6) | 1.1-8.6 | 3.8  (1.6) | 3.5  (2.3-5.1) | 1.2-6.4 |
| Lymphocytes (10^9^/l) | 2.5  (1.0) | 2.2  (1.7-3.3) | 0.9-4.9 | 2.5  (1.1) | 2.2  (1.6-3.4) | 0.8-4.9 | 2.5  (1.2) | 2.25  (1.55-3.2) | 0.8-6.2 |
| Monocytes (10^9^/l) | 0.52  (0.23) | 0.5  (0.32-0.6) | 0.25-1.2 | 0.51  (0.23) | 0.43  (0.36-0.6) | 0.29-1.2 | 0.49  (0.25) | 0.41  (0.35-0.5) | 0.27-1.45 |
| Eosinophils (10^9^/l) | 0.33  (0.42) | 0.22  (0.1-0.4) | 0-2.08 | 0.37  (0.52) | 0.22  (0.13-0.4) | 0.06-2.58 | 0.31  (0.30) | 0.2  (0.11-0.4) | 0-1.26 |
| Basophils (10^9^/l) | 0.06  (0.05) | 0.04  (0.01-0.1) | 0-0.2 | 0.06  (0.04) | 0.05  (0.02-0.1) | 0-0.15 | 0.04  (0.04) | 0.04  (0.1-0.07) | 0-0.1 |

^a^ Missing value from one participant

|  | Visit 5 (21 days)(n=26) | | | Visit 6 (28 days)(n=28) | | | Visit 7 (42 days)(n=28) | | |
| --- | --- | --- | --- | --- | --- | --- | --- | --- | --- |
| **Full blood count** | Mean  (SD) | Median (IQR) | Range | Mean  (SD) | Median (IQR) | Range | Mean  (SD) | Median (IQR) | Range |
| Haemoglobin (g/L) | 125.0  (13.0) | 125  116-135 | 101-149 | 126.1  14.5 | 127  116-134.5 | 95-156 | 125.3  13.6 | 126.5  114.5-133 | 92-152 |
| Haematocrit (L/L) | .37  .04 | .36  .34-.4 | .32-.44 | .37  .05 | .38  .34-.41 | .28-.45 | 0.37  .04 | .37  .34-.4 | .3-.45 |
| RBC (10^12^/l) | 4.50a  .45 | 4.55  4.14-4.8 | 3.7-5.6 | 4.54  .51 | 4.53  4.26-4.91 | 3.51-5.78 | 4.52  .43 | 4.52  4.29-4.78 | 3.61-5.56 |
| MCV (fl) | 82.1  4.6 | 82.5  78.6-86 | 72-91 | 82.5  4.8 | 82.3  80.2-86.6 | 70-91 | 82.3  4.6 | 82  78.5-86.9 | 72-90 |
| MCH (pg) | 27.7a  2.0 | 27.9  26-28.9 | 22.5-31 | 27.9  1.9 | 28.2  26.4-29.4 | 22.8-31 | 27.7  2.0 | 27.7  26.3-29.4 | 22.7-31 |
| Platelets (10^9^/l) | 313.1  119.8 | 278  236-350 | 173-631 | 303.8  101.2 | 301.5  224-350 | 162-586 | 300.5  99.7 | 296.5  227-331.5 | 169-588 |
| White blood cells (10^9^/l) | 8.1  3.3 | 7.95  5.9-9.3 | 3.9-17.5 | 7.3  2.3 | 7.5  5.5-8.6 | 3.4-12.9 | 7.3  2.5 | 6.8  5.8-8.1 | 4-15.2 |
| Neutrophils (10^9^/l) | 4.2a  2.3 | 4  2.2-5.3 | 1.8-10.7 | 3.9  1.8 | 3.3  2.8-4.8 | 1.5-9.3 | 3.9  2.2 | 3.45  2.65-4.25 | 1.6-11.8 |
| Lymphocytes (10^9^/l) | 2.64  1.2 | 2.55  1.7-3.4 | .7-5.1 | 2.54  1.1 | 2.55  1.65-3.1 | .8-5.8 | 2.56  1.0 | 2.45  1.8-3.2 | .9-4.9 |
| Monocytes (10^9^/l) | .55  .27 | .49  .37-.6 | .29-1.4 | .49  .21 | .47  .3-.6 | .27-1.23 | 0.51  0.27 | 0.46  0.36-0.6 | 0.2-1.4 |
| Eosinophils (10^9^/l) | .33  .26 | .2  .13-.5 | 0-1.01 | .29  .27 | .15  .1-.38 | .07-1.1 | 0.27  0.23 | 0.17  0.1-0.45 | 0.05-0.92 |
| Basophils (10^9^/l) | .05  .04 | .06  .03-.1 | 0-.1 | .06  .05 | .05  .02-.09 | 0-.21 | 0.05  0.04 | 0.05  0.01-0.1 | 0-0.11 |

^a^ Missing value from one participant

|  | Visit 8 (56 days)(n=28) | | | Visit 9 (70 days)(n=26) | | | Visit 10 (84 days)(n=27) | | |
| --- | --- | --- | --- | --- | --- | --- | --- | --- | --- |
| **Full blood count** | Mean  (SD) | Median (IQR) | Range | Mean  (SD) | Median (IQR) | Range | Mean  (SD) | Median (IQR) | Range |
| Haemoglobin (g/L) | 124.7  13.9 | 126.5  115.5-135 | 89-154 | 125.4  12.6 | 129  116-136 | 101-147 | 125.8  14.5 | 128  118-138 | 82-148 |
| Haematocrit (L/L) | 0.37  0.04 | 0.37  0.34-0.40 | 0.29-0.45 | 0.38  0.04 | 0.38  0.35-0.41 | 0.3-0.44 | 0.37  0.04 | 0.37  0.34-0.4 | 0.27-0.44 |
| RBC (10^12^/l) | 4.51a  (0.46) | 4.53  4.32-4.72 | 3.55-5.7 | 4.6  0.5 | 4.6  4.3-4.8 | 3.34-5.7 | 4.53  0.49 | 4.6  4.23-4.8 | N=25  3.38-5.66 |
| MCV (fl) | 82.0  4.7 | 81.3  79.4-86.5 | 73-91 | 82.3  5.2 | 82.1  79-87 | 71-91 | 82.1  5.1 | 80.8  79-86.9 | 71-91 |
| MCH (pg) | 27.6a  2.2 | 27.7  26.1-28.9 | 22.1-32 | 27.7  2.1 | 27.7  26.1-29.8 | 22.8-31 | 27.6  2.2 | 28  25.8-28.9 | N=25  22.8-31.3 |
| Platelets (10^9^/l) | 278.2  102.7 | 262.5  220.5-317.5 | 85-582 | 290.0  86.2 | 282  234-340 | 148-504 | 301.8  98.7 | 297  232-338 | 151-630 |
| White blood cells (10^9^/l) | 7.0  2.2 | 6.65  5.4-8.45 | 3.5-12.7 | 7.2  2.7 | 6.8  5.2-8.4 | 3.7-14.4 | 7.4  2.4 | 7.6  6-8.9 | 3.3-14.1 |
| Neutrophils (10^9^/l) | 3.7  1.8 | 3.2  2.45-4.6 | 1.4-9.3 | 3.7  2.0 | 3  2.5-4.2 | 1.1-8.8 | 3.7  2.0 | 3.2  2.3-4.5 | 1.5-10 |
| Lymphocytes (10^9^/l) | 2.5  1.0 | 2.45  1.7-3 | 0.7-5.5 | 2.5  1.2 | 2.35  1.5-3.6 | 0.8-5 | 2.7  1.3 | 2.4  1.6-3.6 | 0.7-6 |
| Monocytes (10^9^/l) | 0.50  (0.21) | 0.45  0.39-0.55 | 0.2-1.22 | 0.53  0.31 | 0.50  0.4-0.56 | 0.2-1.67 | 0.51  0.21 | 0.41  0.37-0.7 | 0.2-1 |
| Eosinophils (10^9^/l) | 0.25  (0.20) | 0.2  0.1-0.3 | 0-0.85 | 0.28  0.23 | 0.19  0.1-0.4 | 0.05-0.72 | 0.35  0.29 | 0.28  0.16-0.4 | 0.07-1.19 |
| Basophils (10^9^/l) | 0.06a  0.05 | 0.05  0.02-0.1 | 0-0.21 | 0.06  0.06 | 0.06  0.02-0.1 | 0-0.27 | 0.06  0.06 | 0.06  0-0.1 | 0-0.29 |

^a^ Missing value from one participant

|  | Visit 11 (98 days)(n=26) | | | Visit 12 (112 days)(n=27) | | | Visit 13 (126 days)(n=26) | | |
| --- | --- | --- | --- | --- | --- | --- | --- | --- | --- |
| **Full blood count** | Mean  (SD) | Median (IQR) | Range | Mean  (SD) | Median (IQR) | Range | Mean  (SD) | Median (IQR) | Range |
| Haemoglobin (g/L) | 124.6  10.5 | 125  120-131 | 105-149 | 122.7  13.0 | 122  118-131 | 84-150 | 121.0  13.6 | 122  112-130 | 82-147 |
| Haematocrit (L/L) | 0.37  0.03 | 0.37  0.35-0.39 | 0.31-0.44 | 0.37  0.38 | 0.36  0.34-0.39 | 0.29-0.46 | 0.36  0.04 | 0.36  0.33-0.39 | 0.26-0.44 |
| RBC (10^12^/l) | 4.53  0.42 | 4.50  4.24-4.8 | 3.99-5.85 | 4.52a  0.46 | 4.59  4.3-4.79 | 3.57-5.51 | 4.43  0.46 | 4.48  4.22-4.72 | 3.45-5.43 |
| MCV (fl) | 81.9  5.0 | 81.5  77.9-86 | 71-91 | 81.6  5.0 | 82  79-85.8 | 71-91 | 81.3  5.5 | 81.2  77.5-86.6 | 71-90 |
| MCH (pg) | 27.6  2.1 | 27.75  26.2-29.3 | 22.2-31 | 27.2a  2.1 | 27.65  25.8-28.6 | 22.1-30.5 | 27.3  2.1 | 27.7  25.9-28.7 | 22.1-30.7 |
| Platelets (10^9^/l) | 293.8  96.9 | 286  219-340 | 151-556 | 291.3a  95.9 | 295  220-327 | 156-645 | 290.3  93.8 | 275.5  240-332 | 157-607 |
| White blood cells (10^9^/l) | 7.7  3.1 | 6.4  5.9-9.3 | 3.7-16.5 | 7.1  2.0 | 6.9  5.5-8.6 | 4.1-10.9 | 7.2  2.8 | 6.55  5.4-8 | 3.8-16.2 |
| Neutrophils (10^9^/l) | 3.95  2.6 | 2.9  2.3-4.1 | 1.8-11 | 3.5  1.3 | 3.4  2.7-4.4 | 1.5-6.7 | 3.65  2.4 | 3.1  2.4-4 | 1.3-11.5 |
| Lymphocytes (10^9^/l) | 2.8  1.1 | 2.7  2.1-3.3 | 0.7-6.2 | 2.7  1.2 | 2.5  2-3.2 | 0.6-5.8 | 2.4  1.1 | 2.35  1.5-3 | 0.7-5.7 |
| Monocytes (10^9^/l) | 0.55  0.27 | 0.5  0.37-0.6 | 0.26-1.3 | 0.53  0.25 | 0.5  0.39-0.63 | 0.28-1.42 | 0.56  0.37 | 0.47  0.32-0.66 | 0.21-1.9 |
| Eosinophils (10^9^/l) | 0.29  0.23 | 0.2  0.11-0.36 | 0.09-0.8 | 0.26  0.20 | 0.2  0.1-0.3 | 0.06-0.97 | 0.38  0.33 | 0.2  0.13-0.54 | 0.06-1.3 |
| Basophils (10^9^/l) | 0.06  0.05 | 0.07  0.01-0.1 | 0-0.17 | 0.04  0.04 | 0.04  0-0.06 | 0-0.11 | 0.06a  0.04 | 0.05  0.04-0.1 | 0-0.17 |

^a^ Missing value from one participant

|  | Visit 14 (140 days)(n=25) | | | Visit 15 (154 days)(n=27) | | | Visit 16 (168 days)(n=26) | | |
| --- | --- | --- | --- | --- | --- | --- | --- | --- | --- |
| **Full blood count** | Mean  (SD) | Median (IQR) | Range | Mean  (SD) | Median (IQR) | Range | Mean  (SD) | Median (IQR) | Range |
| Haemoglobin (g/L) | 124.6  10.7 | 123  120-133 | 104-145 | 121.7  16.2 | 124  114-135 | 82-151 | 124.9  12.1 | 125  117-133 | 99-145 |
| Haematocrit (L/L) | 0.37  0.03 | 0.36  0.35-0.4 | 0.30-0.43 | 0.36  0.05 | 0.37  0.33-0.41 | 0.25-0.45 | 0.37  0.04 | 0.37  0.35-0.4 | 0.28-0.44 |
| RBC (10^12^/l) | 4.49  0.46 | 4.44  4.22-4.82 | n=23  3.52-5.46 | 4.49  0.56 | 4.5  4.24-4.9 | N=25  3.23-5.6 | 4.53  0.48 | 4.61  4.17-4.69 | N=24  3.45-5.52 |
| MCV (fl) | 82.2  4.9 | 83  79-85.5 | 70-89.8 | 81.9  5.1 | 82  77.9-85.2 | 73-90.7 | 82.1  4.9 | 82.2  78.7-85 | 73-91.2 |
| MCH (pg) | 27.8  2.1 | 28.2  26.4-29.2 | N=23  22-31.5 | 27.3  2.2 | 27.7  25.4-28.7 | N=25  22.1-31 | 27.5  1.9 | 27.9  26.3-28.7 | N=24  22.1-30.4 |
| Platelets (10^9^/l) | 284.5  83.5 | 280  217-327 | 166-485 | 309.5  105.5 | 309  231-360 | 148-656 | 281.7  69.9 | 285.5  225-319 | 169-449 |
| White blood cells (10^9^/l) | 7.6  3.2 | 7.4  5.7-8.3 | 3.8-19.8 | 6.8  2.1 | 6.8  5.2-7.9 | 3.1-11.6 | 7.55  3.2 | 6.55  5.6-8.6 | 3.7-17.6 |
| Neutrophils (10^9^/l) | 4.1  3.0 | 3.2  2.7-3.9 | 1.2-16.5 | 3.4  1.6 | 2.7  2.4-4 | 1.7-7.6 | 4.1  2.7 | 3.1  2.5-4.7 | 1.8-13.6 |
| Lymphocytes (10^9^/l) | 2.5  1.2 | 2.4  1.5-3 | 0.6-5.7 | 2.5  1.1 | 2.4  1.6-3.4 | 0.8-5 | 2.5  1.2 | 2.5  1.8-2.9 | 0.7-6.8 |
| Monocytes (10^9^/l) | 0.55  0.3 | 0.5  0.38-0.6 | 0.23-1.3 | 0.51  0.27 | 0.44  0.34-0.52 | 0.2-1.3 | 0.56  0.3 | 0.5  0.37-0.6 | 0.3-1.5 |
| Eosinophils (10^9^/l) | 0.35  0.38 | 0.2  0.1-0.56 | 0-1.59 | 0.27  0.22 | 0.2  0.1-0.36 | 0.05-0.93 | 0.25  0.21 | 0.18  0.1-0.3 | 0-0.81 |
| Basophils (10^9^/l) | 0.06a  0.04 | 0.06  0.02-0.1 | 0-0.14 | 0.05  0.04 | 0.05  0.03-0.1 | 0-0.11 | 0.05  0.04 | 0.05  0.02-0.08 | 0-0.1 |

^a^ Missing value from one participant

|  | Visit 17 (196 days)(n=28) | | | Visit 18 (224 days)(n=27) | | | Visit 19 (252 days)(n=26) | | |
| --- | --- | --- | --- | --- | --- | --- | --- | --- | --- |
| **Full blood count** | Mean  (SD) | Median (IQR) | Range | Mean  (SD) | Median (IQR) | Range | Mean  (SD) | Median (IQR) | Range |
| Haemoglobin (g/L) | 124.2  11.6 | 125.5  116.5-133 | 93-153 | 124.6  11.5 | 122  115-134 | 108-149 | 126.7  12.9 | 126  116-135 | 105-154 |
| Haematocrit (L/L) | 0.37  0.04 | 0.37  0.34-0.4 | 0.26-0.46 | 0.37  0.03 | 0.37  0.34-0.39 | 0.32-0.44 | 0.37a  0.04 | 0.36  0.35-0.40 | 0.31-0.46 |
| RBC (10^12^/l) | 4.49  0.45 | 4.51  4.29-4.66 | N=26  3.2-5.4 | 4.49a  0.44 | 4.55  4.15-4.67 | 3.81-5.68 | 4.49  0.49 | 4.46  4.21-4.83 | N=24  3.52-5.41 |
| MCV (fl) | 82.2  5.0 | 82.6  79.35-86 | 71-91 | 82.0  4.7 | 82  79.4-86 | 72-92 | 82.4a  5.4 | 82.1  78.2-86 | 69-92.4 |
| MCH (pg) | 27.6  1.9 | 28.05  26.2-29 | N=26  22.6-30.3 | 27.75a  2.1 | 28.1  26.4-29.1 | 21.8-31 | 28.1  1.9 | 28.4  27.1-29.6 | N=24  22.7-31 |
| Platelets (10^9^/l) | 289.6  92.8 | 293.5  225.5-323 | 163-569 | 285.2  76.0 | 273  233-326 | 168-447 | 268.2  82.3 | 253.5  207-314 | 151-424 |
| White blood cells (10^9^/l) | 7.1  2.3 | 6.9  5.45-8.95 | 3.2-12.1 | 7.1  2.0 | 6.9  5.7-7.9 | 3.9-14.7 | 6.7  2.4 | 6.15  4.7-8 | 3.5-11.4 |
| Neutrophils (10^9^/l) | 3.7  1.9 | 3.2  2.15-4.8 | 1.5-8.7 | 3.5  1.6 | 3.3  2.46-4 | 1.5-9.6 | 3.5  2.1 | 2.7  2-3.9 | 1.3-9.1 |
| Lymphocytes (10^9^/l) | 2.43  1.15 | 2.2  1.6-3.1 | 0.6-6.1 | 2.66  1.0 | 2.6  2.2-3.4 | 0.7-4.5 | 2.9a  1.0 | 2.3  1.4-2.8 | 0.5-4.9 |
| Monocytes (10^9^/l) | 0.53  0.29 | 0.47  0.40-0.6 | 0.24-1.7 | 0.51  0.24 | 0.43  0.4-0.54 | 0.27-1.36 | 0.47a  0.24 | 0.41  0.3-0.5 | 0.26-1.2 |
| Eosinophils (10^9^/l) | 0.31  0.33 | 0.2  0.1-0.41 | 0-1.6 | 0.28  0.20 | 0.2  0.1-0.43 | 0.06-0.75 | 0.27a  0.23 | 0.2  0.1-0.43 | 0.05-0.93 |
| Basophils (10^9^/l) | 0.05  0.04 | 0.05  0.01-0.08 | 0-0.1 | 0.05  0.04 | 0.05  0-0.09 | 0-0.1 | 0.04a  0.03 | 0.04  0.02-0.06 | 0-0.1 |

^a^ Missing value from one participant

|  | Visit 20 (280 days)(n=26) | | | Visit 21 (308 days)(n=26) | | | Visit 22 (336 days)(n=25) | | |
| --- | --- | --- | --- | --- | --- | --- | --- | --- | --- |
| **Full blood count** | Mean  (SD) | Median (IQR) | Range | Mean  (SD) | Median (IQR) | Range | Mean  (SD) | Median (IQR) | Range |
| Haemoglobin (g/L) | 124.7  11.6 | 119.5  116-133 | 106-150 | 125.3  12.0 | 124.5  116-133 | 106-155 | 122.4  14.0 | 124  111-134 | 98-154 |
| Haematocrit (L/L) | 0.37  0.04 | 0.35  0.34-0.40 | 0.31-0.47 | 0.37  0.04 | 0.37  0.34-0.4 | 0.31-0.47 | 0.36  0.04 | 0.35  0.34-0.39 | 0.3-0.46 |
| RBC (10^12^/l) | 4.49a  0.43 | 4.48  4.15-4.73 | 3.77-5.32 | 4.54  0.46 | 4.61  4.26-4.74 | 3.61-5.74 | 4.39  0.44 | 4.4  4.14-4.7 | 3.46-5.19 |
| MCV (fl) | 81.9  4.9 | 81.8  79-86.6 | 73-89.7 | 82.4  5.3 | 81.45  79.3-87 | 70-91.1 | 82.6  5.2 | 82  80-86 | 70-91 |
| MCH (pg) | 27.8a  2.0 | 28  26.9-28.3 | 22.7-31 | 27.8  2.1 | 28.3  26.3-29.1 | 21.3-31 | 27.9  2.0 | 27.8  26.7-29.2 | 21.6-31.2 |
| Platelets (10^9^/l) | 282.6  82.0 | 277.5  222-326 | 149-528 | 287.8  79.8 | 267.5  237-334 | 160-447 | 281.4  81.1 | 268  224-312 | 172-481 |
| White blood cells (10^9^/l) | 7.3  2.6 | 6.75  5.5-8.2 | 3.6-15.2 | 7.3  2.5 | 7.4  5.4-8.4 | 3.5-14.7 | 6.5  1.6 | 6.4  5.4-7.4 | 3.5-10.2 |
| Neutrophils (10^9^/l) | 3.78  2.3 | 3.3  2.07-4.7 | 1.4-11 | 3.76  1.78 | 3.47  2.5-4.5 | 1.5-8.9 | 3.27  1.2 | 3.1  2.6-4 | 1.5-6 |
| Lymphocytes (10^9^/l) | 2.53  1.1 | 2.25  1.9-3.2 | 0.7-6.1 | 2.51  1.1 | 2.4  1.7-3.2 | 0.7-5.5 | 2.3  0.8 | 2.2  1.7-2.8 | 0.6-4.1 |
| Monocytes (10^9^/l) | 0.54  0.31 | 0.45  0.4-0.52 | 0.29-1.65 | 0.55  0.31 | 0.46  0.38-0.6 | 0.2-1.46 | 0.48  0.2 | 0.44  0.32-0.57 | 0.23-1.08 |
| Eosinophils (10^9^/l) | 0.27  0.25 | 0.18  0.1-0.44 | 0.01-1.12 | 0.30  0.29 | 0.2  0.1-0.4 | 0.04-1.14 | 0.28  0.21 | 0.24  0.1-0.4 | 0.04-0.91 |
| Basophils (10^9^/l) | 0.05  0.04 | 0.05  0-0.08 | 0-0.11 | 0.05  0.04 | 0.05  0-0.07 | 0-0.18 | 0.06  0.08 | 0.04  0.03-0.07 | 0-0.4 |

^a^ Missing value from one participant

|  | Visit 23 (364 days) | | | Visit 24 (392 days) | | | Visit 25 (420 days) | | |
| --- | --- | --- | --- | --- | --- | --- | --- | --- | --- |
| **Full blood count** | Mean  (SD) | Median (IQR) | Range | Mean  (SD) | Median (IQR) | Range | Mean  (SD) | Median (IQR) | Range |
| Haemoglobin (g/L) | 126.3  11.1 | 126  116-135 | 107-147 | 124.1  11.4 | 125.5  113.5-132 | 107-153 | 127.2  13.45 | 125  116-138 | 106-157 |
| Haematocrit (L/L) | 0.37  0.03 | 0.37  0.34-0.4 | 0.31-0.43 | 0.37  0.04 | 0.36  0.34-0.39 | 0.32-0.47 | 0.37  0.04 | 0.37  0.338  0.401 | 0.30  0.47 |
| RBC (10^12^/l) | 4.54  0.47 | 4.49  4.31-4.8 | 3.6-5.93 | 4.475  0.535 | 4.475  4.08-4.71 | 3.58-5.9 | 4.54  0.54 | 4.39  4.24-4.99 | 3.48-5.73 |
| MCV (fl) | 82.0  5.06 | 82  79-85 | 67-90 | 82.35  5.13 | 81.9  79.45-86.75 | 69-89.9 | 82.4  5.0 | 82.1  80.25-86.85 | 68-90 |
| MCH (pg) | 27.95  2.01 | 27.8  27-29.2 | 21.2-31.5 | 27.9  2.0 | 28.05  27-28.9 | 21.7-31.8 | 28.05  1.97 | 28.4  26.8-28.85 | 21.6-31.4 |
| Platelets (10^9^/l) | 279.8  61.85 | 271  237-325 | 168-415 | 295.5  84.3 | 289.5  230-370.5 | 167-450 | 291.6  108.8 | 278  227-373 | 101-591 |
| White blood cells (10^9^/l) | 6.48  1.62 | 6.6  5.6-6.9 | 3.7-11.3 | 7.14  2.66 | 6.7  5.7-7.65 | 3.3-14.6 | 7.36  2.51 | 7.2  5.6-8.8 | 3.9-13.6 |
| Neutrophils (10^9^/l) | 3.21  1.26 | 3  2.4-3.9 | 1.5-6.5 | 3.63  2.15 | 3.35  2.17-3.9 | 1.4-10.5 | 3.96  1.89 | 3.3  2.7-4.6 | 1.5-8.8 |
| Lymphocytes (10^9^/l) | 2.37  0.98 | 2.3  1.7-3 | 0.6-5.4 | 2.53  0.92 | 2.45  1.95-3.15 | 0.9-4.7 | 2.51  0.94 | 2.6  1.85-3 | 0.7-5.4 |
| Monocytes (10^9^/l) | 0.46  0.20 | 0.42  0.32-0.5 | 0.24-1.1 | 0.52  0.24 | 0.415  0.39-0.505 | 0.3-1.4 | 0.53  0.28 | 0.49  0.39-0.61 | 0.2-1.5 |
| Eosinophils (10^9^/l) | 0.30  0.27 | 0.2  0.1-0.36 | 0.07-1 | 0.34  0.26 | 0.30  0.11-0.46 | 0.07-0.95 | 0.27  0.15 | 0.24  0.12-0.4 | 0.07-0.6 |
| Basophils (10^9^/l) | 0.04  0.03 | 0.05  0.02-0.06 | 0-0.1 | 0.05  0.04 | 0.06  0.03-0.09 | 0-0.1 | 0.05  0.04 | 0.05  0.03-0.09 | 0-0.1 |

^a^ Missing value from one participant

|  | Visit 26 (448 days) | | | Visit 27 (476 days) | | | Visit 28 (504 days) | | |
| --- | --- | --- | --- | --- | --- | --- | --- | --- | --- |
| **Full blood count** | Mean  (SD) | Median (IQR) | Range | Mean  (SD) | Median (IQR) | Range | Mean  (SD) | Median (IQR) | Range |
| Haemoglobin (g/L) | 125.8  12.0 | 124.5  120-131.5 | 107-150 | 126.2  13.21 | 122.5  118-136 | 99-158 | 128.0  16.3 | 126.5  118-142 | 91-159 |
| Haematocrit (L/L) | 0.37  0.04 | 0.36  0.35-0.40 | 0.32-0.46 | 0.37  0.04 | 0.36  0.35-0.40 | 0.28-0.47 | 0.38  0.05 | 0.37  0.35-0.41 | 0.28-0.46 |
| RBC (10^12^/l) | 4.50  0.48 | 4.47  4.18-4.71 | 3.71-5.66 | 4.53  0.52 | 4.57  4.21-4.82 | 3.23-5.64 | 4.56  0.60 | 4.6  4.12-4.9 | 3.26-5.79 |
| MCV (fl) | 82.53  5.23 | 81.85  79.5-87.4 | 69-91 | 82.68  5.05 | 81.8  79.4-87.2 | 69-90.4 | 83.21  5.12 | 82.9  80-87 | 70-91 |
| MCH (pg) | 27.99  1.79 | 28.4  26.8-29.1 | 22.3-31.1 | 27.95  2.08 | 28.05  26.8-29.3 | 21.1-31.6 | 27.95  1.98 | 28  26.9-29.1 | 21.6-31.1 |
| Platelets (10^9^/l) | 286.2  85.0 | 272.5  227-324.5 | 159-465 | 294.8  95.95 | 280  213-338 | 163-602 | 280.1  82.8 | 261  226-316 | 145-506 |
| White blood cells (10^9^/l) | 7.06  2.1 | 6.55  5.7-8.05 | 4-12 | 6.97  3.09 | 6.75  4.8-7.6 | 3.8-19.1 | 6.70  2.28 | 6.3  5-7.8 | 3.4-14.5 |
| Neutrophils (10^9^/l) | 3.58  1.61 | 3.2  2.64-3.86 | 1.7-8.5 | 3.63  2.73 | 2.85  2-4.2 | 1.3-14.8 | 3.7  2.15 | 3.2  2.2-4.6 | 1.3-12 |
| Lymphocytes (10^9^/l) | 2.47  0.77 | 2.6  1.85-2.95 | 0.7-4.4 | 2.46  0.78 | 2.4  1.9-2.9 | 0.7-4 | 2.28  0.83 | 2.3  1.6-2.9 | 0.7-4.2 |
| Monocytes (10^9^/l) | 0.53  0.32 | 0.43  0.38-0.51 | 0.2-1.6 | 0.50  0.23 | 0.44  0.34-0.53 | 0.24-1.2 | 0.45  0.18 | 0.4  0.3-0.6 | 0.2-0.8 |
| Eosinophils (10^9^/l) | 0.33  0.30 | 0.21  0.13-0.41 | 0.06-1.33 | 0.25  0.15 | 0.2  0.12-0.31 | 0.1-0.66 | 0.215  0.17 | 0.165  0.1-0.24 | 0-0.65 |
| Basophils (10^9^/l) | 0.05  0.04 | 0.05  0.03-0.09 | 0-0.11 | 0.05  0.05 | 0.05  0-0.07 | 0-0.2 | 0.04  0.04 | 0.035  0-0.05 | 0-0.1 |

^a^ Missing value from one participant

|  | Visit 29 (532 days) | | | Visit 30 (560 days) | | | Visit 31 (588 days) | | |
| --- | --- | --- | --- | --- | --- | --- | --- | --- | --- |
| **Full blood count** | Mean  (SD) | Median (IQR) | Range | Mean  (SD) | Median (IQR) | Range | Mean  (SD) | Median (IQR) | Range |
| Haemoglobin (g/L) | 129.1  11.6 | 130  118-139 | 112-152 | 128.666  12.973 | 132  118.5-138 | 107-151 | 125.36  13.422 | 126  116-131 | 92-156 |
| Haematocrit (L/L) | 0.38  0.04 | 0.38  0.34-0.41 | 0.33-0.45 | 0.378  0.040 | 0.383  0.341-0.41 | 0.304-0.446 | 0.376  0.043 | 0.38  0.346-0.402 | 0.269-0.48 |
| RBC (10^12^/l) | 4.618  0.474 | 4.64  4.36-4.82 | 3.59-5.85 | 4.608  0.484 | 4.655  4.25-4.935 | 3.48-5.81 | 4.520  0.539 | 4.64  4.19-4.76 | 2.96-5.87 |
| MCV (fl) | 82.74  5.404 | 81.7  80-87.9 | 69-93 | 82.275  4.780 | 81.45  79-87 | 72-90 | 83.1  5.070 | 82.8  80-87.3 | 71-91 |
| MCH (pg) | 28.064  1.937 | 28.1  26.8-29.4 | 22.2-32 | 28.012  1.976 | 28.15  26.8-29.45 | 21.7-30.7 | 27.856  2.226 | 28.1  26.7-29.3 | 21.1-31.3 |
| Platelets (10^9^/l) | 281.56  90.506 | 278  214-295 | 178-575 | 278.043  69.704 | 266  242-299 | 185-474 | 289.56  91.276 | 271  238-308 | 166-586 |
| White blood cells (10^9^/l) | 7.172  3.00 | 6.1  5.3-8.4 | 3.5-14.3 | 7.066  2.269 | 7  5.3-8.35 | 3.7-12.2 | 6.824  2.428 | 6.4  5.2-7.9 | 3-14.1 |
| Neutrophils (10^9^/l) | 3.832  2.708 | 3  2.2-3.9 | 1.2-10.6 | 3.671  2.122 | 2.7  2.2-4.15 | 1.8-9.21 | 3.570  2.051 | 2.8  2.3-4.5 | 1.25-10.5 |
| Lymphocytes (10^9^/l) | 2.504  0.933 | 2.4  2-3 | 0.8-5 | 2.512  0.847 | 2.4  2-2.95 | 1.3-4.5 | 2.34  0.873 | 2.4  1.6-2.7 | 1.1-5.2 |
| Monocytes (10^9^/l) | 0.520  0.259 | 0.44  0.34-0.6 | 0.27-1.32 | 0.494  0.203 | 0.455  0.385-0.6 | 0.1-1.01 | 0.52  0.219 | 0.5  0.38-0.6 | 0.21-1.12 |
| Eosinophils (10^9^/l) | 0.198  0.130 | 0.16  0.1-0.28 | 0-0.61 | 0.248  0.190 | 0.17  0.1-0.435 | 0.06-0.73 | 0.198  0.098 | 0.19  0.12-0.28 | 0.06-0.44 |
| Basophils (10^9^/l) | 0.047  0.037 | 0.04  0.02-0.06 | 0-0.12 | 0.049  0.037 | 0.05  0.02-0.07 | 0-0.12 | 0.044  0.034 | 0.05  0.02-0.07 | 0-0.1 |

^a^ Missing value from one participant

|  | Visit 32 (616 days) | | | Visit 33 (644 days) | | | Visit 34 (672 days) | | |
| --- | --- | --- | --- | --- | --- | --- | --- | --- | --- |
| **Full blood count** | Mean  (SD) | Median (IQR) | Range | Mean  (SD) | Median (IQR) | Range | Mean  (SD) | Median (IQR) | Range |
| Haemoglobin (g/L) | 129.916  13.454 | 131.5  118-136.5 | 104-159 | 125.625  17.120 | 129.5  114-138.5 | 82-150 | 129.826  15.867 | 133  120-138 | 84-154 |
| Haematocrit (L/L) | 0.382  0.043 | 0.372  0.349-0.42 | 0.299-0.49 | 0.375  0.047 | 0.375  0.345-0.41 | 0.251-0.46 | 0.378  0.048 | 0.383  0.351-0.406 | 0.238-0.46 |
| RBC (10^12^/l) | 4.614  0.542 | 4.6  4.26-4.9 | 3.24-5.9 | 4.513  0.647 | 4.59  4.225-4.92 | 2.65-5.78 | 4.61  0.586 | 4.66  4.38-4.92 | 2.72-5.66 |
| MCV (fl) | 83.091  5.554 | 82  80-87.3 | 71-92.3 | 83.160  6.066 | 81  79.5-88 | 70-94.7 | 82.534  5.294 | 81.5  79-86.8 | 71-91.8 |
| MCH (pg) | 28.126  2.057 | 28.1  27.1-29.6 | 22.2-32.1 | 27.925  2.077 | 28.25  26.65-29 | 22.1-31.1 | 28.286  1.966 | 28.4  27-29.8 | 23-31 |
| Platelets (10^9^/l) | 281.75  86.045 | 275  226-328 | 155-512 | 300.304  121.669 | 279  239-350 | 98-680 | 283.363  105.289 | 269  233-294 | 154-568 |
| White blood cells (10^9^/l) | 7.262  2.368 | 7.05  5.35-9.1 | 3.8-11.9 | 7.337  2.266 | 6.85  6.1-8.05 | 3.8-13.2 | 7.930  4.146 | 7.2  6.1-8.4 | 3.6-23 |
| Neutrophils (10^9^/l) | 3.993  2.070 | 3.6  2.3-5.33 | 1.4-8.8 | 3.917  1.745 | 3.32  2.8-4.535 | 1.6-8.3 | 4.460  3.878 | 3.5  2.4-5.1 | 1.5-19.89 |
| Lymphocytes (10^9^/l) | 2.408  0.842 | 2.4  1.8-2.8 | 1.1-4.1 | 2.541  1.130 | 2.6  1.55-3 | 0.8-5.1 | 2.508  0.937 | 2.5  1.7-2.9 | 1.2-4.9 |
| Monocytes (10^9^/l) | 0.506  0.249 | 0.42  0.34-0.56 | 0.27-1.2 | 0.540  0.231 | 0.475  0.4-0.63 | 0.33-1.4 | 0.553  0.304 | 0.5  0.31-0.6 | 0.25-1.6 |
| Eosinophils (10^9^/l) | 0.256  0.201 | 0.18  0.1-0.4 | 0.05-0.81 | 0.211  0.104 | 0.185  0.13-0.305 | 0.07-0.4 | 0.269  0.265 | 0.14  0.1-0.48 | 0.02-1.19 |
| Basophils (10^9^/l) | 0.043  0.035 | 0.04  0-0.08 | 0-0.1 | 0.036  0.031 | 0.03  0.005-0.06 | 0-0.1 | 0.055  0.032 | 0.05  0.04-0.08 | 0-0.1 |

^a^ Missing value from one participant

# Supplementary table 3: Summary of biochemistry results

|  | Visit 2 (baseline) (n=27) | | | Visit 3 (7 days) (n=27) | | | Visit 4 (14 days)(n=28) | | |
| --- | --- | --- | --- | --- | --- | --- | --- | --- | --- |
| **Biochemistry** | Mean  (SD) | Median (IQR) | Range | Mean  (SD) | Median (IQR) | Range | Mean  (SD) | Median (IQR) | Range |
| Sodium | 138.6  (1.9) | 139  (137-140) | 135-142 | 138.8  (2.0) | 139  (138-140) | 133-142 | 138.5  (1.6) | 139  (137-139.5) | 135-141 |
| Potassium | 4.3a  (0.3) | 4.35  (4-4.5) | 3.3-5 | 4.29  (0.28) | 4.3  (4.1-4.4) | 3.8-4.9 | 4.29  (0.4) | 4.3  (4-4.55) | 3.6-5.4 |
| Urea | 5.4  (2.0) | 5.4  (3.9-6) | 2.2-11.4 | 5.4  (1.7) | 5.1  (4.3-6.6) | 2.6-9.2 | 5.3  (1.6) | 5.3  (4.05-5.85) | 2.4-8.9 |
| Creatinine | 55.8  (34.5) | 42  (29-76) | 18-158 | 58.5  (33.4) | 49  (32-80) | 19-158 | 59  (34.8) | 46  (33.5-83) | 19-163 |
| eGFR | 116.0 (37.2) | 117.7 (90.9 to 128.5) | 48.1 to 210.2 | 110.60  (35.2) | 107.5  (89.3-119.2) | 47.8-221.2 | 108.8  (34.7) | 102.5  (85.6-128.8) | 46.0-184.4 |
| Total Protein | 69.9  (4.8) | 70  (68-74) | N=21  61-76 | 71.4  (7.6) | 71  (68-76) | N=21  56-90 | 69.4  (6.0) | 71  (65-74) | N=23  55-81 |
| Albumin | 42.9  (4.4) | 43  (40-46) | 35-50 | 43.2  (5.0) | 43  (40-46) | 33-55 | 42.5  (4.2) | 42.5  (39.5-44.5) | 33-50 |
| Serum calcium | 2.40  (0.1) | 2.41  (2.37-2.43) | N=16  2.19-2.52 | 2.42  (0.1) | 2.4  (2.37-2.49) | N=18  2.3-2.58 | 2.37  (0.07) | 2.38  (2.33-2.43) | N=18  2.24-2.48 |
| Adjusted calcium | 2.40  (0.12) | 2.4  (2.37-2.49) | N=21  2.07-2.56 | 2.42  (0.11) | 2.42  (2.35-2.49) | N=23  2.19-2.61 | 2.39  (0.09) | 2.41  (2.31-2.44) | N=24  2.2-2.57 |
| Phosphate | 1.33  (0.2) | 1.33  (1.28-1.46) | N=21  0.7-1.66 | 1.38  (0.24) | 1.46  (1.34-1.54) | N=25  0.6-1.66 | 1.33  (0.26) | 1.39  (1.17-1.54) | N=26  0.57-1.66 |
| Total bilirubin | 7.0  (5.3) | 5  (3-10) | (3-24) | 8.5a  (6.8) | 6  (4-11) | 3-30 | 7.1  (5.0) | 5  (4-8.5) | 3-23 |
| ALP^a^ | 172.5a  (92.6) | 169  (87-206) | 50-389 | 168.8  (87.5) | 164.5  (90-205) | 54-413 | 165.5  (87.5) | 164.5  (86.5-209) | 51-397 |
| ALT^a^ | 21.0a  (11.6) | 18  (14-24) | (8-64) | 19.3a  (10.8) | 18  (14-21) | 6-62 | 19.1a  (7.1) | 17  (14-24) | 4-35 |
| AST^a^ | 30.4  (14.8) | 25  (20-39) | N=13  13-65 | 30.3  (15.6) | 25  (20-35) | N=14  15-74 | 28.8  (10.6) | 27  (19-36) | N=11  18-49 |
| Lactate Dehydrogenase | 340.2  (144.2) | 301  (217-494) | N=23  138-552 | 316.9  (136.1) | 266  (206-459) | 160-594 | 324  (147.5) | 256  (213-483) | N=26  138-643 |
| Urine PCR | 35.1^a, b^  (50.9) | 17.2  (9.5-34) | 0-221 | No obs | | | | | |

^a^ Missing value from one participant

^b^ Urine PCR was reported in g/mmol as 0.04 normal range is usually between 2 and 200. Sensitivity analysis removing this value had minimal impact on the results; Mean=36.5 (SD= 51.5); median=17,4; IQR= 15.5-34; range=3-221

|  | Visit 5 (21 days)(n=27) | | | Visit 6 (28 days)(n=28) | | | Visit 7 (42 days)(n=28) | | |
| --- | --- | --- | --- | --- | --- | --- | --- | --- | --- |
| **Biochemistry** | Mean  (SD) | Median (IQR) | Range | Mean  (SD) | Median (IQR) | Range | Mean  (SD) | Median (IQR) | Range |
| Sodium | 138.7  2.1 | 138  137-143 | 134-143 | 138.7  1.9 | 139  137-140 | 135-142 | 138.0  2.0 | 138  136-139 | N=26  134-141 |
| Potassium | 4.3  .35 | 4.4  4.1-4.6 | 3.7-5.1 | 4.4  .39 | 4.4  4.1-4.5 | 3.8-5.6 | 4.4  0.3 | 4.3  4.1-4.6 | N=26  3.7-4.9 |
| Urea | 5.0  1.7 | 4.9  3.9-6.2 | 2-9.3 | 5.2  1.8 | 4.7  3.95-5.8 | 3-9.8 | 5.6a  2.2 | 5.2  4.1-6.2 | 2.7-12.2 |
| Creatinine | 58.1  37.6 | 45  31-83 | 18-180 | 59.0  36.8 | 47.5  32-79.5 | 19-187 | 58.7a  (37.1) | 47  33-85 | 20-178 |
| eGFR | 111.3  (35.9) | 108.4  (85.7- 125.5) | 40.8-193.2 | 110.0  (34.7) | 105.7  (90.6-124.5) | 39.0-211.6 | 112.2  (36.9) | 110.5  (90.4-126.3) | N=25  41.4-193.2 |
| Total Protein | 69.9  8.1 | 70  64-75 | N=23  55-91 | 70.6  6.5 | 70.5  66.5-75.5 | N=24  55-81 | 68.7a  6.3 | 68  63-74 | N=23  57-86 |
| Albumin | 42.2  4.1 | 42  40-45 | 33-49 | 42.7  4.1 | 43  40-44 | 35-52 | 42.8  3.5 | 43.5  40-45 | N=26  36-48 |
| Serum calcium | 2.42  .08 | 2.43  2.36-2.46 | N=17  2.32-2.6 | 2.40  .09 | 2.39  2.33-2.45 | N=17  2.24-2.54 | 2.40  0.09 | 2.39  2.34-2.45 | N=17  2.2-2.59 |
| Adjusted calcium | 2.44  .08 | 2.45  2.4-2.48 | N=25  2.21-2.6 | 2.41  .07 | 2.42  2.34-2.47 | N=23  2.29-2.53 | 2.39  0.10 | 2.39  2.32-2.45 | N=24  2.24-2.64 |
| Phosphate | 1.33  .21 | 1.37  1.18-1.51 | .85-1.71 | 1.37  .25 | 1.39  1.22-1.58 | N=25  .85-1.74 | 1.35  0.22 | 1.36  1.22-1.53 | N=25  0.87-1.7 |
| Total bilirubin | 7^b^  6.7 | 5  3-7 | 2-29 | 7.5  5.5 | 6  4-9.5 | 3-22 | 8.3a  (6.9) | 6  (4-10) | 3-29 |
| ALP^a^ | 170.4  84.9 | 175  89-215 | 49-415 | 167.3  88.6 | 169.5  87-213 | 47-445 | 170.5  90.6 | 168  88-218 | 45-451 |
| ALT^a^ | 19.6  13.8 | 17  13-21 | 5-80 | 20.6a  15.2 | 16  12-24 | 6-77 | 18.7  (9.3) | 17  12-21 | 8-50 |
| AST^a^ | 26.2  9.2 | 23  19-35 | N=12  15-40 | 26.2  11.0 | 22  19-33 | N=13  12-51 | 26.5  9.6 | 24  18-33 | N=13  14-45 |
| Lactate Dehydrogenase | 310.1^a, c^  142.6 | 250.5  192-402 | 141-637 | 300.9a  133.1 | 243  183-388 | 150-611 | 302.1  126.6 | 261  196-369 | N=25  140-594 |

^a^ Missing value from one participant

^b^ Total Bilirubin’ included an ‘actual lab value of < 3’ for one participant but the value entered was 3. Sensitivity analysis excluding this value had a minimal impact, with only the mean being slightly increased to 7.2 (SD=6.8), otherwise all other values remained the same

^c^ Lactate Dehydrogenase was recorded as 247 in one participant, but result should be interpreted with caution due to the haemolysis level which was above the normal acceptable level for this test. Sensitivity analysis excluding this value had a minimal impact, with only the mean being slightly increased to 312.6 (SD=144.9), median to 154 (IQR=192-402), with the range unhanged.

|  | Visit 8 (56 days)(n=28) | | | Visit 9 (70 days)(n=26) | | | Visit 10 (84 days)(n=27) | | |
| --- | --- | --- | --- | --- | --- | --- | --- | --- | --- |
| **Biochemistry** | Mean  (SD) | Median (IQR) | Range | Mean  (SD) | Median (IQR) | Range | Mean  (SD) | Median (IQR) | Range |
| Sodium | 138.8  1.8 | 139  137-140.5 | 136-143 | 138.5  2.1 | 138.5  138-140 | 132-142 | 139.1  1.9 | 139  138-143 | 135-143 |
| Potassium | 4.4  0.5 | 4.3  4.1-4.6 | 3.8-6.5 | 4.3  0.4 | 4.3  4.1-4.6 | 3.8-5.2 | 4.4  0.3 | 4.4  4-4.5 | 3.8-5.1 |
| Urea | 5.68  3.5 | 5.1  3.95-6.25 | 2.3-22 | 5.72  2.7 | 5.25  3.9-6.4 | 2.8-15.6 | 5.58  2.5 | 5.6  4-6.4 | 2.6-15.5 |
| Creatinine | 62.0  52.0 | 50  33.5-77.5 | 20-296 | 62  49.0 | 46  31-78 | 21-259 | 62.1  50.3 | 51  32-81 | 21-283 |
| eGFR | 111.0 (34.9) | 106.4  (86.7- 130.3) | 22.4-184.4 | 108.1  (35.9) | 99.1  (87.9-131.9) | 26.3-181.4 | 108.9  (34.0) | 105.7  (84.1-129.3) | 23.6-174.8^a^ |
| Total Protein | 67.9  5.4 | 67  65-73 | N=25  55-77 | 67.6  6.4 | 69  63-70 | N=22  57-85 | 68.9  5.9 | 68  65.5-73.5 | N=24  54-80 |
| Albumin | 42.6  4.1 | 42.5  40-45 | 33-50 | 42.2  3.5 | 43  39-45 | 36-48 | 42.7  3.7 | 42  40-45 | 34-52 |
| Serum calcium | 2.34  0.17 | 2.39  2.28-2.43 | N=16  1.81-2.55 | 2.37  0.09 | 2.39  2.31-2.4 | N=16  2.2-2.49 | 2.37  0.10 | 2.4  2.29-2.44 | N=17  2.18-2.51 |
| Adjusted calcium | 2.36  0.15 | 2.38  2.34-2.42 | N=24  1.79-2.64 | 2.38  0.08 | 2.42  2.32-2.45 | N=24  2.21-2.52 | 2.39  0.10 | 2.4  2.32-2.47 | N=24  2.19-2.55 |
| Phosphate | 1.32  0.24 | 1.37  1.21-1.52 | N=24  0.86-1.62 | 1.36  0.24 | 1.38  1.24-1.56 | N=4  0.79-1.73 | 1.36  0.26 | 1.40  1.24-1.52 | N=24  0.87-1.86 |
| Total bilirubin | 7.5  6.1 | 6  3.5-9.5 | 3-31 | 6.9  4.7 | 5  4-10 | 3-22 | 7.7^b^  7.7 | 5  4-8 | 3-37 |
| ALP^a^ | 173.6  93.6 | 177  88-232 | 41-455 | 193.3  120.9 | 183  88-240 | 45-596 | 183.7  95.9 | 177  98-259 | 50-430 |
| ALT^a^ | 21.6a  19.9 | 15  13-21 | 8-110 | 18.5  8.5 | 18  13-23 | 7-49 | 17.8a  9.7 | 16  11-21 | 6-47 |
| AST^a^ | 51.5  92.5 | 23.5  18-41 | N=14  14-370 | 28.5  8.1 | 32  22-35 | N=11  16-41 | 29.6  8.8 | 31.5  21-35 | N=14  15-44 |
| Lactate Dehydrogenase | 328.3  167.5 | 281  195-468 | N=26  149-841 | 302.3  119.6 | 269  204-378 | 170-613 | 298  112.0 | 260  205-377 | 172-575 |
| Urine PCR | Not recorded at these visits | | | | | | 30.5  33.2 | 15.2  10.3-38.5 | N=24  2.8-114.4 |

^a^ Missing value from one participant

^b^ Total Bilirubin’ included an ‘actual lab value of < 3’ for one participant but the value entered was 3. Sensitivity analysis excluding this value had a minimal impact, with only the mean being slightly increased to 7.9 (SD=7.8), otherwise all other values remained the same

|  | Visit 11 (98 days)(n=26) | | | Visit 12 (112 days)(n=27) | | | Visit 13 (126 days)(n=26) | | |
| --- | --- | --- | --- | --- | --- | --- | --- | --- | --- |
| **Biochemistry** | Mean  (SD) | Median (IQR) | Range | Mean  (SD) | Median (IQR) | Range | Mean  (SD) | Median (IQR) | Range |
| Sodium | 139.1  1.7 | 139  138-140 | 135-142 | 138.3a  2.0 | 139  137-139 | 134-142 | 139.6a  2.8 | 139  138-141 | 136-150 |
| Potassium | 4.3a  0.5 | 4.3  3.9-4.5 | 3.6-5.3 | 4.3a  0.3 | 4.4  4-4.5 | 3.8-5.1 | 4.4a  0.4 | 4.2  4.1-4.7 | 3.8-5.2 |
| Urea | 5.65  3.1 | 5.05  3.8-6.3 | 2.3-18 | 5.77a  2.5 | 5.2  4.2-6.6 | 3.4-14.2 | 5.96a  3.3 | 5.1  4.3-6.1 | 2.2-19.2 |
| Creatinine | 64.9  60.5 | 48  32-77 | 22-330 | 61.4a  51.1 | 44  33-76 | 20-274 | 65.3a  62.7 | 46  31-83 | 19-331 |
| eGFR | 108.0  (36.2) | 104.5  (81.2-126.0) | 19.6-178.0 | 110.6  (39.2) | 104.0  (83.9-134.2) | N=24  24.6-204.7 | 110.9  (41.2) | 100.4  (86.6-149.7) | N=23  19.5-186.1 |
| Total Protein | 69.6a  6.4 | 70  67-75 | 50-80 | 69.0  6.7 | 70  66-72 | N=25  51-84 | 68.2  7.4 | 69  63-72 | N=23  54-86 |
| Albumin | 42.9  4.5 | 43  41-45 | 30-51 | 42.4  3.5 | 42  40-45 | 31-48 | 42.0  4.1 | 42.5  39-46 | 33-47 |
| Serum calcium | 2.39  0.1 | 2.43  2.34-2.49 | N=18  2.15-2.52 | 2.39  0.1 | 2.4  2.3-2.49 | N=17  2.1-2.52 | 2.39  0.09 | 2.41  2.33-2.44 | N=16  2.21-2.52 |
| Adjusted calcium | 2.41  0.10 | 2.40  2.33-2.46 | 2.23-2.6 | 2.39a  0.10 | 2.39  2.31-2.45 | 2.24-2.57 | 2.40  0.09 | 2.41  2.36-2.46 | N=24  2.24-2.52 |
| Phosphate | 1.36  0.27 | 1.39  1.12-1.53 | 0.92-1.94 | 1.35  0.24 | 1.38  1.21-1.48 | N=25  0.87-1.7 | 1.39a  0.23 | 1.47  1.28-1.53 | 0.83-1.72 |
| Total bilirubin | 7.5  5.9 | 5  4-10 | 3-28 | 7.3^c^  5.9 | 5  3-9 | 3-31 | 7.3a  5.0 | 6  4-9 | 3-26 |
| ALP^a^ | 184.5  96.0 | 171  94-247 | 48-422 | 182.2  91.5 | 186  92-248 | 52-353 | 184.0  90.5 | 184  94-253 | 53-381 |
| ALT^a^ | 17.7a  10.0 | 16  13-22 | 5-55 | 19.8a  12.0 | 15  13-22 | 6-61 | 18.7a  9.4 | 16  13-22 | 7-51 |
| AST^a^ | 27  8.2 | 24  22-32 | N=13  14-42 | 30.3  8.0 | 32  24-35 | N=13  18-42 | 27.8  8.5 | 27.5  21-33.5 | N=16  14-43 |
| Lactate Dehydrogenase | 294.5^a, b^  110.6 | 249  215-374 | 169-599 | 285.6  102.4 | 269  209.5-338 | N=24  145-583 | 280.3  96.9 | 247  217-343 | 137-568 |
| Urine PCR | Not recorded at these visits | | | | | | | | |

^a^ Missing value from one participant

^b^ Lactate Dehydrogenase was recorded as 277 in one participant, but result should be interpreted with caution due to the haemolysis level which was above the normal acceptable level for this test. Sensitivity analysis excluding this value had a minimal impact, with only the mean being slightly increased to 295.2 (SD=112.9), median to 242.5 (IQR=210-388.5), with the range unhanged.

^c^ Total Bilirubin’ included an ‘actual lab value of < 3’ for one participant but the value entered was 3. Sensitivity analysis excluding this value had a minimal impact, with only the mean being slightly increased to 7.5 and lower quartile (25^th^ percentile) increasing to 4, otherwise all other values remained the same

|  | Visit 14 (140 days)(n=27) | | | Visit 15 (154 days)(n=27) | | | Visit 16 (168 days)(n=26) | | |
| --- | --- | --- | --- | --- | --- | --- | --- | --- | --- |
| **Biochemistry** | Mean  (SD) | Median (IQR) | Range | Mean  (SD) | Median (IQR) | Range | Mean  (SD) | Median (IQR) | Range |
| Sodium | 138.5a  2.7 | 139  137-140 | 132-144 | 138.8  1.9 | 139  137-140 | 134-142 | 138.2  1.8 | 138  137-140 | 133-141 |
| Potassium | 4.33a  0.39 | 4.3  4.1-4.6 | 3.5-5.2 | 4.42  0.34 | 4.4  4.2-4.7 | 3.9-5 | 4.33a  0.44 | 4.3  4-4.7 | 3.5-5.3 |
| Urea | 5.9a  3.2 | 5.2  4.5-6.3 | 2.6-18.8 | 5.7  2.5 | 5  4.1-7 | 1.9-14 | 5.7  2.4 | 5.35  4.1-6.2 | 2.9-13 |
| Creatinine | 62.8a  50.5 | 51.5  30-75 | 22-272 | 63.2  44.9 | 53  31-91 | 21-235 | 60.0  40.8 | 49  32-76 | 18-207 |
| eGFR | 111.0  (37.5) | 109.6  (87.9-132.1) | N=25  24.8-189.4 | 107.0  (36.3) | 101.3  (81.3-132.6) | 29.6-173.6 | 110.2  (36.1) | 105.5  (83.8-124.8) | 34.5-178.7 |
| Total Protein | 67.9  6.4 | 68  66-70 | N=21  47-79 | 67.9  6.8 | 69  65.5-71.5 | N=24  47-80 | 68.1  6.9 | 68  65-72 | 52-86 |
| Albumin | 42.5  4.7 | 44  40-46 | 28-49 | 42.4  4.9 | 43  40-45 | 28-50 | 42.3  4.4 | 43  39-45 | 29-49 |
| Serum calcium | 2.40  0.13 | 2.43  2.35-2.5 | N=17  2.02-2.52 | 2.39  0.12 | 2.42  2.32-2.47 | N=15  2.09-2.57 | 2.36  0.12 | 2.38  2.29-2.43 | 2.14-2.52 |
| Adjusted calcium | 2.40a  0.09 | 2.39  2.35-2.45 | 2.24-2.55 | 2.39  0.09 | 2.4  2.33-2.45 | N=25  2.23-2.56 | 2.38a  0.09 | 2.38  2.34-2.43 | 2.18-2.55 |
| Phosphate | 1.33a  0.24 | 1.43  1.11-1.51 | 0.8-1.5 | 1.36  0.23 | 1.34  1.22 | N=25  0.79-1.69 | 1.33a  0.23 | 1.36  1.21-1.49 | 0.76-1.69 |
| Total bilirubin | 7.4  6.0 | 6  4-9 | N=25  3-31 | 6.5a  4.1 | 5  3-9 | 3-21 | 7.2^a, b^  4.8 | 6  4-10 | 3-22 |
| ALP^a^ | 173.6a  89.6 | 175  90-239 | 45-376 | 176.8  92.3 | 164  84-263 | 47-347 | 184.3  99.5 | 180  84-266.5 | N=24  53-423 |
| ALT^a^ | 18.5  8.1 | 16  12-22 | N=25  6-37 | 17.8  8.8 | 16  12-21 | 7-47 | 18.8a  9.5 | 15  13-21 | 10-44 |
| AST^a^ | 26.3  6.6 | 28  21-32 | N=12  17-36 | 28.1  11.9 | 27  20-34 | N=18  8-60 | 28.9  9.6 | 28  21-35 | N=15  17-52 |
| Lactate Dehydrogenase | 280.6  111.5 | 242.5  203-323 | N=24  158-578 | 291.4  121.3 | 256  205-349 | N=22  153-585 | 282.5a  127.4 | 242  209-307 | 148-630 |
| Urine PCR | Not recorded at these visits | | | | | | 38.6  65.7 | 15.1  10.4-34 | 3n=21  3-303 |

^a^ Missing value from one participant

^b^ ‘Total Bilirubin’ in V16 at Liverpool site for participant 02001 included an ‘actual lab value of < 3’ but the value entered was 3. Sensitivity analysis excluding this value had a minimal impact, with only the mean being slightly increased to 7.4 and the upper quartile (75^th^ percentile) increasing to 10.5, otherwise all other values remained the same.

|  | Visit 17 (196 days)(n=28) | | | Visit 18 (224 days)(n=27) | | | Visit 19 (252 days)(n=27) | | |
| --- | --- | --- | --- | --- | --- | --- | --- | --- | --- |
| **Biochemistry** | Mean  (SD) | Median (IQR) | Range | Mean  (SD) | Median (IQR) | Range | Mean  (SD) | Median (IQR) | Range |
| Sodium | 139.0  1.7 | 139  138-140 | 134-142 | 139  1.9 | 139  138-140 | 135-144 | 138.8a  1.7 | 139  138-140 | 134-142 |
| Potassium | 4.3  0.4 | 4.15  4-4.55 | 3.6-5.4 | 4.3a  0.4 | 4.2  4.1-4.5 | 3.6-5.3 | 4.35a  0.45 | 4.25  4.1-4.5 | 3.7-5.4 |
| Urea | 5.6  2.35 | 5.1  4-6.15 | 2.3-12 | 5.5  2.2 | 5.1  3.8-6.7 | 2.6-11.7 | 5.8  2.0 | 5.6  3.9-7.2 | 3-10.9 |
| Creatinine | 59.9  38.5 | 56  31-70.5 | 19-203 | 57.6  29.5 | 54  33-79 | 21-135 | 62.2  40.6 | 53  34-80 | 21-210 |
| eGFR | 114.2  (39.6) | 108.5  (90.8- 129.6) | N=25  35.3-208.3 | 111.9  (36.3) | 104.7  (88.2-124.5) | 46.6-198.4^a^ | 108.9  (37.0) | 101.9  (85.0-133.8) | N=25  33.9-198.4 |
| Total Protein | 68.7  6.9 | 67.5  66-72 | N=24  49-85 | 68.1  7.7 | 69  66-73 | N=24  48-80 | 67.9  7.6 | 68  65-72 | N=25  49-81 |
| Albumin | 42.1  4.3 | 43  40-44.5 | 29-49 | 42.1  4.9 | 43  40-44 | 27-49 | 42.2a  4.9 | 42.5  40-45 | 28-51 |
| Serum calcium | 2.39  0.13 | 2.42  2.36-2.45 | N=17  2.08-2.57 | 2.37  0.12 | 2.4  2.36-2.43 | N=17  2.03-2.5 | 2.40  0.11 | 2.40  2.36-2.45 | N=16  2.17-2.6 |
| Adjusted calcium | 2.40  0.10 | 2.42  2.31-2.47 | N=26  2.26-2.6 | 2.39a  0.08 | 2.4  2.33-2.43 | 2.23-2.54 | 2.40  0.08 | 2.39  2.36-2.42 | N=25  2.28-2.63 |
| Phosphate | 1.34  0.25 | 1.34  1.14-1.57 | N=26  0.86-1.8 | 1.39a  0.22 | 1.44  1.18-1.54 | 0.84-1.72 | 1.33  0.24 | 1.36  1.13-1.52 | N=25  0.89-1.77 |
| Total bilirubin | 8.15a  6.7 | 5  4-11 | 3-28 | 7.2a  5.2 | 5.5  4-9 | 2-26 | 6.9^b^  5.0 | 4  3-9 | N=25  3-21 |
| ALP^a^ | 177.0  98.1 | 160.5  80.5-265.5 | 49-388 | 179.8  99.0 | 157  77-263 | N=25  48-352 | 177.3a  93.9 | 171.5  83-248 | 49-359 |
| ALT^a^ | 17.4  9.5 | 14.5  11.5-20.5 | 5-48 | 17.1a  9.1 | 15  12-19 | 9-50 | 18.9a  13.2 | 14.5  12-21 | 7-67 |
| AST^a^ | 28.9  9.5 | 28  21-35 | N=15  16-47 | 28.5  10.7 | 27  22-35 | N=15  13-56 | 30.6  12.4 | 30  20-37 | N=17  10-56 |
| Lactate Dehydrogenase | 266.6  102.3 | 249.5  203-292 | N=26  152-616 | 261.7  111.8 | 226  193-308 | N=23  146-627 | 277.2  120.6 | 247  192-330 | N=23  141-649 |
| Urine PCR | Not recorded at these visits | | | | | | 4 | 5.4 | 76.2 |

^a^ Missing value from one participant

^b^ Total Bilirubin’ included an ‘actual lab value of < 3’ for one participant but the value entered was 3. Sensitivity analysis excluding this value had a minimal impact, with only the mean being slightly increased to 7.0, median to 4.5 and lower quartile (25^th^ percentile) increasing to 3.5, otherwise all other values remained the same

|  | Visit 20 (280 days)(n=26) | | | Visit 21 (308 days)(n=26) | | | Visit 22 (336 days)(n=25) | | |
| --- | --- | --- | --- | --- | --- | --- | --- | --- | --- |
| **Biochemistry** | Mean  (SD) | Median (IQR) | Range | Mean  (SD) | Median (IQR) | Range | Mean  (SD) | Median (IQR) | Range |
| Sodium | 138.4  1.9 | 139  138-139 | 132-141 | 138.3  2.1 | 139  138-140 | 133-141 | 138.6  2.2 | 139  138-140 | 131-142 |
| Potassium | 4.36  0.5 | 4.25  4-4.5 | 3.6-6 | 4.32  0.47 | 4.2  4-4.6 | 3.6-5.6 | 4.31a  0.3 | 4.25  4.2-4.45 | 3.6-5 |
| Urea | 5.5  1.9 | 5.15  4.3-6.5 | 2.9-10.7 | 5.5  2.0 | 5  3.8-7 | 2.8-10.7 | 5.4  2.1 | 5  4.1-6 | 2.3-10.6 |
| Creatinine | 57.5  28.4 | 56  37-79 | 21-145 | 55.6  28.4 | 48.5  37-71 | 22-144 | 56.8  27.7 | 54  37-66 | 22-158 |
| eGFR | 107.3  (29.9) | 101.0  (88.2-127.5) | N=22  52.6-176.7 | 112.3  (34.9) | 102.5  (87.4-140.3) | N=24  52.7-189.3 | 105.3  (26.6) | 106.2  (86.5-118.4) | N=22  47.1-161.7 |
| Total Protein | 66.8a  8.1 | 67  64-73 | 46-77 | 68.3a  6.0 | 68  65-71 | 48-77 | 66.4a  7.4 | 67  64-71 | 45-76 |
| Albumin | 41.7  5.1 | 43  40-45 | 28-48 | 42.2  3.8 | 43  40-45 | 29-47 | 41.6  5.3 | 42  40-44 | 27-49 |
| Serum calcium | 2.33  0.17 | 2.38  2.23-2.42 | N=17  1.99-2.6 | 2.38  0.09 | 2.41  2.37-2.42 | N=17  2.15-2.48 | 2.36  0.14 | 2.37  2.3-2.47 | N=15  2-2.53 |
| Adjusted calcium | 2.37  0.12 | 2.40  2.33-2.45 | 1.95-2.55 | 2.39  0.08 | 2.39  2.34-2.44 | 2.21-2.6 | 2.41  0.08 | 2.4  2.34-2.48 | N=23  2.26-2.55 |
| Phosphate | 1.34a  0.23 | 1.33  1.26-1.52 | 0.89-1.73 | 1.35  0.24 | 1.37  1.11-1.52 | 0.8-1.77 | 1.38  0.24 | 1.44  1.19-1.61 | N=22  0.86-1.64 |
| Total bilirubin | 7.4  4.7 | 6  3-11 | 2-20 | 7.6^a, b^  6.0 | 6  3-9 | 3-29 | 7.6  6.4 | 5  3-8 | 3-28 |
| ALP^a^ | 182.2  92.7 | 174.5  88-266 | 49-349 | 183.0  93.1 | 172  90-253 | 48-372 | 182a  101.8 | 182  75-253 | 49-373 |
| ALT^a^ | 17.1  7.9 | 14.5  11-20 | 9-44 | 19.1a  11.5 | 15  12-20 | 6-53 | 17.8a  11.4 | 15  11-19 | 7-56 |
| AST^a^ | 28.6  9.6 | 26.5  21-35 | N=14  17-48 | 29  11.1 | 27  19-36 | N=17  13-51 | 26.7  11.7 | 22.5  19-37 | N=18  10-52 |
| Lactate Dehydrogenase | 260.8  110.3 | 223  198-297 | N=23  133-555 | 253.2  102.9 | 232  184.5-292.5 | N=24  141-541 | 255.3a  118.0 | 225  178.5-276 | 150-660 |
| Urine PCR | Not recorded at these visits | | | | | | 34.8^a, d^  64.1 | 15.75  6.5-28.1 | 0.7-304 |

^a^ Missing value from one participant

^b^ Total Bilirubin’ included an ‘actual lab value of < 3’ for one participant but the value entered was 3. Sensitivity analysis excluding this value had a minimal impact, with only the mean being slightly increased to 7.8 (SD=6.1), median to 6.5 and IQR now 3.5-9.5, where range remained the same

^c^ Total Bilirubin’ included an ‘actual lab value of < 3’ for one participant but the value entered was 3. Sensitivity analysis excluding this value had a minimal impact, with only the mean being slightly increased to 7.8 (SD=6.5), median to 5.5 and IQR now 3.5-8.5, where range remained the same

^d^ Urine PCR was reported as 3 but is recorded as 3 but was not sent to lab for testing on same day as other tests.. Sensitivity analysis removing this value had minimal impact on the results; Mean=36.2 (SD=65.2); median=17.3; IQR= 6.9-33; range remains the same.

Table 5h: Summary of biochemistry variables at follow-up visits 23-25

|  | Visit 23 (364 days) | | | Visit 24 (392 days) | | | Visit 25 (420 days) | | |
| --- | --- | --- | --- | --- | --- | --- | --- | --- | --- |
| **Biochemistry** | Mean  (SD) | Median (IQR) | Range | Mean  (SD) | Median (IQR) | Range | Mean  (SD) | Median (IQR) | Range |
| Sodium | 138.52  2.12 | 139  138-140 | 133-141 | 138.04  2.68 | 139  137-140 | 129-141 | 137.8  1.89 | 138  137-139 | 134-141 |
| Potassium | 4.37  0.415 | 4.4  4.2-4.55 | 3.3-5.5 | 4.38  0.35 | 4.3  4.1-4.6 | 3.8-5.3 | 4.37  0.44 | 4.3  4.15-4.55 | 3.2-5.3 |
| Urea | 5.34  2.43 | 4.5  3.6-6.5 | 2.3-12.5 | 5.33  1.84 | 4.95  4-6 | 3.1-11.2 | 6.05  2.24 | 5.3  4.4-6.9 | 3-11.8 |
| Creatinine | 55.88  28.13 | 49  37-71 | 23-150 | 55.79  28.71 | 48  35-69.5 | 23-144 | 63.16  39.16 | 55  44-74 | 22-204 |
| eGFR | 113.1 (35.6) | 106.7  (89.5-134.0) | 50.2-206.6 | 112.0  (29.3) | 107.6  (94.8-134.0) | 52.7-169.6 | 104.0  (30.5) | 103.9  (82.4-122.0) | 34.8-164.5 |
| Total Protein | 67.695  5.78 | 68  65-73 | 49-75 | 66.05  6.81 | 67  63-71 | 47-76 | 67.74  6.843 | 69  64-73 | 50-78 |
| Albumin | 41.92  4.96 | 43  39-45 | 28-49 | 41.88  5.24 | 42.5  40.5-45 | 25-48 | 42.72  4.12 | 43  41-45 | 30-49 |
| Serum calcium | 2.34  0.15 | 2.39  2.27-2.425 | 2.02-2.58 | 2.35  0.14 | 2.395  2.29-2.425 | 2.02-2.56 | 2.37  0.13 | 2.39  2.32-2.45 | 1.99-2.6 |
| Adjusted calcium | 2.37  0.09 | 2.37  2.3-2.4 | 2.22-2.6 | 2.38  0.09 | 2.38  2.31-2.44 | 2.2-2.59 | 2.38  0.08 | 2.385  2.34-2.43 | 2.19-2.58 |
| Phosphate | 1.38  0.23 | 1.46  1.2-1.545 | 0.88-1.65 | 1.34  0.26 | 1.45  1.24-1.51 | 0.67-1.64 | 1.39  0.30 | 1.37  1.21-1.59 | 0.71-2.09 |
| Total bilirubin | 6.4^b^  3.19 | 6  5-8 | 3-17 | 6.79  4.98 | 5.5  4-7 | 3-25 | 7.04^d^  4.568 | 5  4-9 | 3-18 |
| ALP^a^ | 194.82  110.41 | 196  82-263 | 59-492 | 181.17  103.31 | 166  80-253 | 53-420 | 187.1  109.50 | 179  77-278 | 61-431 |
| ALT^a^ | 20.32^c^  11.345 | 17  13-24 | 9-63 | 17.25  10.37 | 15  12.5-19.5 | 7-60 | 17.71  7.91 | 15.5  13.5-19.5 | 7-45 |
| AST^a^ | 30.64  12.89 | 28.5  20-36 | 17-55 | 27.93  10.57 | 25  19-34 | 16-54 | 28.57  10.44 | 24.5  21-38 | 16-51 |
| Lactate Dehydrogenase | 252.26  124.67 | 224  176-262 | 4-509 | 266.09  118.314 | 235  202-272 | 139-575 | 235.35  86.14 | 226  183-259 | 132-536 |
| Urine PCR | Not recorded at these visits | | | | | | | | |

^a^ Missing value from one participant

^b^ Total Bilirubin’ included an ‘actual lab value of < 3’ for one participant but the value entered was 3. Sensitivity analysis excluding this value had a minimal impact, with only the mean being slightly increased to 6.5, otherwise all other values remained the same.

^c^ ALT is recorded as 32 but result may be overestimated due to haemolysis. Sensitivity analysis excluding this value had a minimal impact, with only the mean being slightly decreased to 19.8, otherwise all other values remained the same.

^d^ Total Bilirubin’ included an ‘actual lab value of < 3’ for one participant but the value entered was 3. Sensitivity analysis excluding this value had a minimal impact, with only the mean being slightly increased to 7.2, median to 5.5 and upper quartile range (75^th^ percentile) to 9.5, otherwise all other values remained the same.

|  | Visit 26 (448 days) | | | Visit 27 (476 days) | | | Visit 28 (504 days) | | |
| --- | --- | --- | --- | --- | --- | --- | --- | --- | --- |
| **Biochemistry** | Mean  (SD) | Median (IQR) | Range | Mean  (SD) | Median (IQR) | Range | Mean  (SD) | Median (IQR) | Range |
| Sodium | 138.67  1.66 | 139  137.5-140 | 135-141 | 138.85  2.053 | 138  138-140 | 133-144 | 138.33  2.27 | 138  137-140 | 133-143 |
| Potassium | 4.32  0.42 | 4.2  4-4.6 | 3.5-5.4 | 4.44  0.465 | 4.3  4.2-4.6 | 3.8-6.1 | 4.28  0.40 | 4.2  4-4.5 | 3.7-5.4 |
| Urea | 5.46  2.15 | 4.6  4-7.05 | 2.3-11.6 | 5.53  2.40 | 5.05  3.7-7.15 | 2.3-11.2 | 5.77  2.36 | 5.3  3.9-7 | 2.2-11.6 |
| Creatinine | 54.25  27.59 | 51  31.5-69 | 22-148 | 57.88  29.96 | 55  32-72 | 24-151 | 62.19  36.24 | 56  33-78 | 23-163 |
| eGFR | 114.7  (33.4) | 111.6  (95.3- 125.1) | 51.0-196.0 | 111.1  (38.0) | 102.6  (77.9-137.4)c | 49.8-210.9 | 107.9  (33.4) | 105.5  (84.1-127.9) | 45.4-172.5 |
| Total Protein | 67.96  7.689 | 69  65-71 | 52-84 | 68.67  6.565 | 69  65.5-72.5 | 52-86 | 68.807  6.876 | 69  65-75 | 48-79 |
| Albumin | 41.91  4.36 | 43  39-45 | 32-48 | 42.5  3.635 | 42.5  41-45 | 33-48 | 42.37  4.21 | 42  40-46 | 28-48 |
| Serum calcium | 2.34  0.17 | 2.39  2.31-2.44 | 1.85-2.57 | 2.375  0.109 | 2.4  2.33-2.45 | 2.07-2.48 | 2.37  0.12 | 2.38  2.36-2.44 | 2.05-2.55 |
| Adjusted calcium | 2.375  0.13 | 2.35  2.315-2.505 | 1.99-2.59 | 2.39  0.075 | 2.39  2.34-2.445 | 2.21-2.52 | 2.39  0.08 | 2.4  2.33-2.45 | 2.22-2.57 |
| Phosphate | 1.34  0.196 | 1.32  1.18-1.52 | 0.95-1.67 | 1.34  0.22 | 1.425  1.18-1.495 | 0.82-1.59 | 1.28  0.285 | 1.25  1.05-1.54 | 0.69-1.83 |
| Total bilirubin | 6.78^b^  4.81 | 5  4-8 | 3-23 | 8.24^d^  6.935 | 6  4-8 | 3-29 | 8.15^f^  5.51 | 7  5-9 | 3-24 |
| ALP^a^ | 194.70  109.42 | 174  79-293 | 60-438 | 178  95.16 | 169  79-256 | 58-339 | 179.23  107.71 | 150.5  82-277 | 43-435 |
| ALT^a^ | 27.25  56.62 | 15.5  12-20 | 8-292 | 18.56  9.07 | 16  13-23 | 8-54 | 17.85  7.64 | 16  13-22 | 10-42 |
| AST^a^ | 30.41  21.59 | 23  19-32 | 13-104 | 29.18  11.73 | 27  20-38 | 16-52 | 26.9  10.42 | 24.5  19.5-33.5 | 14-56 |
| Lactate Dehydrogenase | 240.22^c^  84.42 | 218  182-278 | 150-511 | 248.42^e^  101.25 | 224  172-288 | 129-557 | 238.36  72.30 | 214  190-270 | 132-468 |
| Urine PCR | Not recorded at these visits | | | | | | 32.9  (47.8) | 16.3  (7.7-35.5) | 1.1-198 |

^a^ Missing value from one participant

^b^ Total Bilirubin’ included an ‘actual lab value of < 3’ for two participants but the value entered was 3. Sensitivity analysis excluding these values increased the mean value to 7.0 (SD=4.9), otherwise all other values remained the same.

^c^ Lactate Dehydrogenase was recorded as 277 in one participant, but result should be interpreted with caution due to the haemolysis level which was above the normal acceptable level for this test. Sensitivity analysis excluding this value had a minimal impact, with only the mean being slightly decreased to 235.4 (SD=83.1), median to 215.5 (IQR=182-251), with the range unchanged.

^d^ Total Bilirubin’ included an ‘actual lab value of < 3’ for two participants but the value entered was 3. Sensitivity analysis excluding these values increased the mean value to 8.5 (SD=7.0), otherwise all other values remained the same.

^e^ Lactate Dehydrogenase was recorded as 404 in one participant, but result should be interpreted with caution due to the haemolysis level which was above the normal acceptable level for this test. Sensitivity analysis excluding this value had a minimal impact, with only the mean being slightly decreased to 241.7 (SD=97.8), median to 223 (IQR=166-279), with the range unchanged.

^f^ Total Bilirubin’ included an ‘actual lab value of < 3’ for one participant but the value entered was 3. Sensitivity analysis excluding this value increased the mean to 8.3, otherwise all other values remained the same.

|  | Visit 29 (532 days) | | | Visit 30 (560 days) | | | Visit 31 (588 days) | | |
| --- | --- | --- | --- | --- | --- | --- | --- | --- | --- |
| **Biochemistry** | Mean  (SD) | Median (IQR) | Range | Mean  (SD) | Median (IQR) | Range | Mean  (SD) | Median (IQR) | Range |
| Sodium | 138.28  1.514 | 138  137-140 | 136-141 | 137.78  3.25 | 138  137-140 | 126-141 | 138.48  2.00 | 138  138-140 | 133-142 |
| Potassium | 4.26  0.37 | 4.2  4.1-4.3 | 3.8-5.5 | 4.29  0.33 | 4.2  4-4.5 | 3.8-4.9 | 4.17^e^  0.38 | 4.2  4-4.4 | 3.3-4.9 |
| Urea | 5.664  2.122 | 5  4.3-6.8 | 2.9-11.1 | 5.27  1.80 | 5  4.2-6.2 | 2.8-11 | 5.00  1.84 | 4.35  4-6.05 | 2.4-11.1 |
| Creatinine | 58.28  28.47 | 52  36-77 | 23-141 | 54.48  24.66 | 44  34-79 | 24-101 | 53.24  21.74 | 52  34-69 | 20-99 |
| eGFR | 108.7  (29.9) | 105.5  (80.6- 130.5) | 54.1-161.1 | 111.6  (30.4) | 104.6  (86.6-132.0) | 75.4-168.7 | 117.7  (32.8) | 111.2  (90.2-134.9) | 77.6-180.9 |
| Total Protein | 67.58  6.95 | 68  65-72.5 | 52-79 | 68.26  7.19 | 71  65-73 | 48-78 | 67.33  8.63 | 68  65.5-72.5 | 45-79 |
| Albumin | 42.04  4.40 | 42.5  40-45 | 31-49 | 42.52  4.67 | 43  40-46 | 30-50 | 42.24  5.88 | 43  39-46 | 27-50 |
| Serum calcium | 2.37  0.11 | 2.4  2.37-2.44 | 2.14-2.51 | 2.36  0.11 | 2.38  2.34-2.41 | 2.04-2.5 | 2.35^i^  0.15 | 2.37  2.33-2.45 | 1.94-2.57 |
| Adjusted calcium | 2.39  0.08 | 2.38  2.315-2.41 | 2.27-2.54 | 2.38  0.08 | 2.38  2.31-2.43 | 2.24-2.51 | 2.38  0.08 | 2.4  2.36-2.43 | 2.2-2.54 |
| Phosphate | 1.33  0.26 | 1.355  1.145-1.505 | 0.79-1.79 | 1.33  0.20 | 1.31  1.23-1.47 | 0.9-1.67 | 1.28  0.19 | 1.33  1.14-1.45 | 0.87-1.55 |
| Total bilirubin | 8.29^b^  5.75 | 6  4.5-11 | 3-25 | 7.55  4.22 | 6  5-11 | 3-17 | 7.80^d^  6.00 | 7  3-8 | 2.99-28 |
| ALP^a^ | 188.21  103.41 | 167  85-291.5 | 54-350 | 196.68  110.03 | 196  82-291 | 51-415 | 194.08  108.75 | 200  83-253 | 52-425 |
| ALT^a^ | 18  7.84 | 16.5  13-21 | 5-40 | 18.77^b^  9.16 | 15  13-22 | 5-42 | 17^f^  8.82 | 15  12-21 | 8-52 |
| AST^a^ | 27.59  10.56 | 26  21-33 | 15-55 | 29  10.60 | 25  22-37 | 15-53 | 27.53^g^  10.13 | 24  20-35 | 14-51 |
| Lactate Dehydrogenase | 250.76  82.96 | 233  199-294 | 133-497 | 242^c^  85.93 | 230  195-247 | 139-510 | 219.35^h^  57.36 | 205  183-253 | 143-376 |
| Urine PCR | Not recorded at these visits | | | | | | | | |

^a^ Missing value from one participant

^b^ ALT is recorded as 13 but Sample taken as a repeat. Sensitivity analysis excluding this value increased the mean to 19.0 (SD=19.3), otherwise all other values remained the same.

^c^ Lactate Dehydrogenase was recorded as 346 in one participant, but result should be interpreted with caution due to the haemolysis level which was above the normal acceptable level for this test. Sensitivity analysis excluding this value had a minimal impact, with only the mean being slightly decreased to 236.8 (SD=84.7), median to 226.5 (IQR=185-244), with the range unchanged.

^d^ Total Bilirubin’ included an ‘actual lab value of < 3’ for two participants but the value entered was 3. Sensitivity analysis excluding these values increased the mean value to 8.0 (SD=6.0), lower quartile (25^th^ percentile) to 3.5, otherwise all other values remained the same.

^e^ Potassium value should be 4.8 but result unavailable due to haemolysis and any value should be used with caution. Sensitivity analysis excluding these values decreased the mean value to 4.1, otherwise all other values remained the same.

^f^ ALT value should be 13 but result unavailable due to haemolysis and any value should be used with caution. Sensitivity analysis excluding these values increased the mean value to 17.2 (SD=9.0), median to 15.5, otherwise all other values remained the same.

^g^ AST value should be 31 but result unavailable due to haemolysis and any value should be used with caution. Sensitivity analysis excluding these values increased the mean value to 27.3 (SD=10.4), median to 23.5 (IQR=19.5-35.5), while range remained the same.

^h^ Lactate Dehydrogenase was recorded as 376 in one participant, but result should be interpreted with caution due to the haemolysis level which was above the normal acceptable level for this test. Sensitivity analysis excluding this value had a minimal impact, with only the mean being slightly decreased to 212.2 (SD=47.2), median to 203 (IQR=183-239), with the range unchanged.

^i^ Serum calcium was recorded as 1.94and was derived from corrected calcium result using: serum Ca= cCa-0.02(40-albumin). Sensitivity analysis excluding this value had a minimal impact, with only the mean being slightly increased to 2.4 (SD=0.1), median to 2.39 (IQR=2.33-2.45), with the range unchanged.

|  | Visit 32 (616 days) | | | Visit 33 (644 days) | | | Visit 34 (672 days) | | |
| --- | --- | --- | --- | --- | --- | --- | --- | --- | --- |
| **Biochemistry** | Mean  (SD) | Median (IQR) | Range | Mean  (SD) | Median (IQR) | Range | Mean  (SD) | Median (IQR) | Range |
| Sodium | 138.71  1.85 | 139  138-139.5 | 135-143 | 138.79  2.08 | 139  137-140.5 | 136-143 | 138.41  3.28 | 139  136.5-141 | 129-144 |
| Potassium | 4.25  0.28 | 4.2  4-4.5 | 3.8-4.7 | 4.20  0.31 | 4.1  3.9-4.3 | 3.8-4.9 | 4.18  0.31 | 4.2  4-4.4 | 3.2-4.7 |
| Urea | 5.87  2.39 | 5.5  4.25-7.25 | 2.6-11.7 | 5.23  1.59 | 5.1  4-6.2 | 2.8-9.3 | 5.43  1.68 | 5.3  4.1-6.35 | 3.4-9.1 |
| Creatinine | 57.75  29.44 | 53  35-73.5 | 25-153 | 55.63  23.05 | 57  37.5-72.5 | 23-102 | 55.58  23.59 | 53  35.5-77 | 24-106 |
| eGFR | 109.1  (33.7) | 103.9  (82.0-133.7) | 49.3-191.6 | 112.6  (32.4) | 104.7  (86.0-147.4) | 61.5-171.5 | 109.8  (30.3) | 101.7  (85.6-123.1) | 73.1-188.8 |
| Total Protein | 68.43  7.81 | 69  65-73 | 48-81 | 69.64  7.86 | 69.5  66-76 | 51-85 | 68.43  8.83 | 69  65-73 | 43-82 |
| Albumin | 42.46  5.20 | 42.5  39.5-46 | 29-51 | 41.83  5.53 | 42.5  39-45.5 | 27-50 | 41.88  5.66 | 42  40-44.5 | 24-52 |
| Serum calcium | 2.39  0.10 | 2.395  2.35-2.475 | 2.16-2.53 | 2.36  0.14 | 2.36  2.31-2.45 | 2.03-2.59 | 2.36  0.15 | 2.375  2.29-2.46 | 1.94-2.62 |
| Adjusted calcium | 2.41  0.08 | 2.38  2.35-2.48 | 2.21-2.56 | 2.40  0.09 | 2.4  2.345-2.48 | 2.24-2.61 | 2.40  0.09 | 2.41  2.33-2.43 | 2.26-2.62 |
| Phosphate | 1.36  0.23 | 1.38  1.19-1.53 | 0.94-1.73 | 1.27  0.20 | 1.23  1.13-1.45 | 0.85-1.69 | 1.26  0.24 | 1.25  1.04-1.47 | 0.88-1.65 |
| Total bilirubin | 8.67^b^  8.47 | 5  4-9 | 3-38 | 8.57^c^  7.86 | 6  4-10 | 3-38 | 8.65  6.86 | 6  3-13 | 3-31 |
| ALP^a^ | 202.77  108.91 | 191.5  106-292 | 45-390 | 191.54  100.52 | 195.5  84-262 | 46-373 | 187.43  97.92 | 184  103-266 | 46-372 |
| ALT^a^ | 21  11.55 | 16  13-21 | 12-48 | 18.88  12.70 | 14.5  12-19.5 | 10-66 | 19.79  10.57 | 16.5  13-20.5 | 8-52 |
| AST^a^ | 30  10.38 | 29.5  22-39 | 16-49 | 27.69  9.60 | 25  20-34.5 | 16-48 | 27.72  8.92 | 25  22-34 | n=23  17-51 |
| Lactate Dehydrogenase | 227.85  49.72 | 234.5  189-269 | 134-307 | 223.41  44.37 | 228.5  196-253 | 140-295 | 247.43  66.90 | 246  215-268 | 137-444 |
| Urine PCR | Not recorded at these visits | | | | | | 30.6  (52.5) | 16.3  (7.5-24) | 0.1-234 |

^a^ Missing value from one participant

^b^ Total Bilirubin’ included an ‘actual lab value of < 3’ for two participants but the value entered was 3. Sensitivity analysis excluding these values increased the mean value to 8.9 (SD=8.6), upper quartile (75^th^ percentile) to 10, otherwise all other values remained the same.

^c^ Total Bilirubin’ included an ‘actual lab value of < 3’ for two participants but the value entered was 3. Sensitivity analysis excluding these values increased the mean value to 9.1 (SD=8.0), lower quartile (25^th^ percentile) to 5 and upper quartile (75% percentile) to 10, otherwise all other values remained the same.

^d^ Lactate Dehydrogenase was recorded as 295 in one participant, but this result was from biochemistry sample taken at a different time to the other results. Sensitivity analysis excluding this value had a minimal impact, with only the mean being slightly decreased to 220 (SD=42.4), median to 227 (IQR=196-249), with the range unchanged.

# Supplementary table 4. Summary of reported Serious Adverse Events (SAEs)

| **Event Term** | **Seriousness** | **Severity** | **Causality^1^** | **Outcome** | **Comments** |
| --- | --- | --- | --- | --- | --- |
| Presumed Viral URTI | Inpatient Hospitalisation | Mild | unrelated | resolved | N/A |
| aHUS Relapse | Inpatient Hospitalisation | severe | probable | resolved | Patient had eculizumab treatment restarted within the required 24hr time period stipulated in the protocol. |
| viral infection | Inpatient Hospitalisation | Mild | unrelated | resolved | Not reported until October. TM received updated SAE log from site which included this SAE and requested a report be sent immediately. |
| Line infection and removal | Inpatient Hospitalisation | Mild | unrelated | resolved | N/A |
| Streptococcus tonsillitis – group A | Inpatient Hospitalisation | Moderate | unrelated | resolved | National service contacted to discuss patients’ bloods results and potential relapse. PI reviewed patient the next day and intended to re-start Ecu if no improvement however patient had improved and so remains off drug. |
| Relapse of aHUS | Inpatient hospitalisation | Moderate | Definitely | resolved | Patient had recently made a full recovery from streptococcal tonsillitis (13/02/20) though with occasional proteinuria. Significant proteinuria emerged, bloods were suggestive of relapse (raised LDH and falling platelet count) and patient admitted for full assessment. Patient has restarted eculizumab as of 20/03/20 and has recovered |
| Native Renal Biopsy to investigate proteinuria-which pre-dated entry into the trial | Inpatient hospitalisation | Moderate | Unrelated | Condition unchanged | Site completed the SAE form on day they were made aware of event (09.03.2020) however this was not submitted to NCTU until 11 days later (20.03.2020). Updated deviation log has been requested. |
| Salmonella Gastroenteritis | Inpatient hospitalisation | Mild | Unrelated | Recovered | Participant was treated with IV antibiotics, GP informed that if symptoms reappear to treat with oral ciprofloxacin. |
| Hyperkalaemia | Inpatient hospitalisation | Moderate | Unrelated | Recovered | During trial visit potassium level was found to be 6.1. Participant received IV fluids and insulin at local A&E, stayed overnight and was then discharged. |
| Pulmonary Embolism | Inpatient hospitalisation | Severe | Unrelated | Recovered | Bilateral pulmonary embolism diagnosed by CTPA following cycling injury. Patient treated with oral Rivaroxaban |
| Uncontrolled hypertension | Inpatient hospitalisation/prolonged hospitalisation | Moderate | No | Recovered | Patient was admitted TO GNCH 30/12/21 with BP elevated to 160mmHg, patient had increased creatinine and lowered platelets. |
| Reduced Renal Function | Important Medical Event | Moderate | Yes | Recovered with sequalae | Patient admitted with reduced renal function, rising creatinine, hyperkalemia. Eculizumab restarted |
| Prolonged hospitalisation due to respiratory issues after an elective procedure (fundoplication and port-cath change) | Inpatient hospitalisation/prolonged hospitalisation | Moderate | No | Recovered | Prolonged hospitalisation due to respiratory issues after elective procedure (fundoplication and port-cath change) |
| Recurrent small bowel diaphragm disease | Persistent or significant disability/incapacity | Moderate | No | Recovered with sequelae | Weight loss and anaemia due to NSAID induced diaphragm disease. Treated with Iron Isomaltoside and a laxative to prevent obstruction. |
| Idiopathic Transient oedema | Inpatient hospitalisation/prolonged hospitalisation | Mild | No | Recovered | Hospitalised following sudden onset marked lower limb and scrotal oedema, resolved with no treatment. No nephrotic syndrome or heart failure |
| Lower respiratory tract infection, sepsis | Life threatening  Inpatient hospitalisation at trial site | Severe | No | ongoing | Admitted with hyponatremia and oedema, rapid deterioration and intubated on pICU. Diagnosed with LRTI and sepsis |
| Hyponatremia, possible infection | Inpatient hospitalisation | Severe | No | ongoing | Hospitalised with hyponatremia, treated with IV hypertonic saline. Infection treated with ciprofloxacin, piperacillin tazobactam |
| Hyponatremia, Upper respiratory tract infection – viral | Inpatient hospitalisation | Moderate | No | Condition improved | Hospitalised; viral respiratory infection and treated with Azithromycin, Hyponatremia treated with oral sodium chloride. |
| Hyponatremia | Inpatient hospitalisation | Moderate | No | Recovered | Hyponatremia - facial oedema and increased weight. |
| Low Sodium | Inpatient hospitalisation | Moderate | No | Recovered | Hospitalisation - admitted as an inpatient 28/12/2 |

# Supplementary figure 1. Biochemical and haematological parameters for patient 7

Eculizumab reintroduced 19 weeks after withdrawal

# Supplementary figure 2. Biochemical and haematological parameters for patient 8

Eculizumab reintroduced 74 weeks after withdrawal.
