# Supplementary material for: Eculizumab withdrawal and monitoring in atypical haemolytic uraemic syndrome (SETS aHUS): a multicentre, open label, prospective, single arm trial
Source: Lancet Reg Health Eur. 2025 Aug 7;56:101392. doi: 10.1016/j.lanepe.2025.101392 (PMC12811780; doi:10.1016/j.lanepe.2025.101392)
Supplement: Supplementary file 3 [file mmc3.zip › lanepe_101392_Investigator group_mmc3.odt]

| First name | Last name |
| --- | --- |
| Coralie | Bingham |
| Paramit | Chowdhury |
| Tina | Dutt |
| Eric | Finlay |
| Daniel | Gale |
| Amrit | Kaur |
| Michael | Kelly |
| Stephen | Marks |
| Mordi | Muorah |
| Ravindra | Rajakariar |
| Nicholas | Torpey |
| Mark | Uniacke |
| David | Walbaum |
| Pallavi | Yadav |

SETS aHUS Investigator group
